# Reactivation of low avidity tumor-specific CD8^+^ T cells associates with immunotherapeutic efficacy of anti-PD-1

**DOI:** 10.1136/jitc-2023-007114

**Published:** 2023-08-16

**Authors:** Gessa Sugiyarto, Doreen Lau, Samuel Luke Hill, David Arcia-Anaya, Denise S M Boulanger, Eileen E Parkes, Edward James, Tim Elliott

**Affiliations:** 1 Centre for Cancer Immunology, Faculty of Medicine, University of Southampton, Southampton, UK; 2 Centre for Immuno-Oncology, Nuffield Department of Medicine, University of Oxford, Oxford, UK; 3 Department of Oncology, University of Oxford, Oxford, UK

**Keywords:** Antigen Presentation, Antigens, Neoplasm, Immune Checkpoint Inhibitors, Immune Tolerance, Lymphocytes, Tumor-Infiltrating

## Abstract

**Background:**

CD8^+^ T cells are a highly diverse population of cells with distinct phenotypic functions that can influence immunotherapy outcomes. Further insights on the roles of CD8^+^ specificities and TCR avidity of naturally arising tumor-specific T cells, where both high and low avidity T cells recognizing the same peptide-major histocompatibility complex (pMHC) coexist in the same tumor, are crucial for understanding T cell exhaustion and resistance to PD-1 immunotherapy.

**Methods:**

CT26 models were treated with anti-PD-1 on days 3, 6 and 9 following subcutaneous tumor implantation generating variable responses during early tumor development. Tetramer staining was performed to determine the frequency and avidity of CD8^+^ T cells targeting the tumor-specific epitope GSW11 and confirmed with tetramer competition assays. Functional characterization of high and low avidity GSW11-specific CD8^+^ T cells was conducted using flow cytometry and bulk RNA-seq. In vitro cytotoxicity assays and in vivo adoptive transfer experiments were performed to determine the cytotoxicity of high and low avidity populations.

**Results:**

Treatment success with anti-PD-1 was associated with the preferential expansion of low avidity (Tet^lo^) GSW11-specific CD8^+^ T cells with Vβ TCR expressing clonotypes. High avidity T cells (Tet^hi^), if present, were only found in progressing PD-1 refractory tumors. Tet^lo^ demonstrated precursor exhausted or progenitor T cell phenotypes marked by higher expression of Tcf-1 and T-bet, and lower expression of the exhaustion markers CD39, PD-1 and Eomes compared with Tet^hi^, whereas Tet^hi^ cells were terminally exhausted. Transcriptomics analyses showed pathways related to TCR signaling, cytotoxicity and oxidative phosphorylation were significantly enriched in Tet^lo^ found in both regressing and progressing tumors compared with Tet^hi^, whereas genes related to DNA damage, apoptosis and autophagy were downregulated. In vitro studies showed that Tet^lo^ exhibits higher cytotoxicity than Tet^hi^. Adoptive transfer of Tet^lo^ showed more effective tumor control than Tet^hi^, and curative responses were achieved when Tet^lo^ was combined with two doses of anti-PD-1.

**Conclusions:**

Targeting subdominant T cell responses with lower avidity against pMHC affinity neoepitopes showed potential for improving PD-1 immunotherapy. Future interventions may consider expanding low avidity populations via vaccination or adoptive transfer.

**WHAT IS ALREADY KNOWN ON THIS TOPIC**

- T cell avidity plays a crucial role in antigen recognition and influences the quality of TCR signaling and T cell metabolic fitness.

**WHAT THIS STUDY ADDS**

- Low avidity, tumor-specific CD8^+^ T cells are preferentially expanded in responders to PD-1 immunotherapy in preclinical studies.
- Low avidity T cells exhibit ‘precursor exhausted’ or progenitor phenotypes, high cytotoxic function, and are significantly enriched for pathways associated with TCR signaling, cytotoxic function and oxidative phosphorylation.

**HOW THIS STUDY MIGHT AFFECT RESEARCH, PRACTICE OR POLICY**

- Boosting low avidity populations and subdominant T cell responses may enhance treatment efficacy of anti-PD-1.

## Background

Immune checkpoints are inhibitory pathways crucial for the maintenance of self-tolerance and protection of tissues from overt immune destruction during pathogenic infections and tumor eradication.^1^ In antitumor immunity, the amplitude and duration of cytotoxic CD8^+^ T cell response to cancer is initiated through T cell receptor (TCR) recognition of specific antigenic peptides presented on major histocompatibility complex (MHC) class I molecules and is regulated by a balance between costimulatory and coinhibitory signals at the immunological synapse.^2^ Cancer can evade immune destruction via the expression of immune checkpoint proteins and other immunosuppressive molecules.^1^ The immune checkpoint protein programmed cell death 1 (PD-1) is a transmembrane glycoprotein of the immunoglobulin B7-CD28 family, which is important for regulating CD8^+^ T cell functions in peripheral tissues, such as the tumor microenvironment during the effector phase. PD-1 is highly expressed on regulatory T cells (Tregs) and is induced only on CD8^+^ T cells on activation. Increased PD-1 expression in tumor-infiltrating CD8^+^ T cells (TIL) has been associated with T cell exhaustion and poor clinical outcomes.^3^ Concurrently, high expression of the ligand PD-L1 has been reported in various cancers, as well as in immunosuppressive cell types such as Tregs, myeloid-derived suppressor cells and cancer-associated fibroblasts. Upregulation of PD-L1 in tumors is thought to be mediated by the negative feedback mechanisms of interferon gamma (IFNγ) signaling on TCR activation, which indirectly contributes to adaptive immune resistance.^4^

In recent years, monoclonal antibodies targeting PD-1 and PD-L1 have emerged as a promising approach for cancer treatment.^5^ This class of immunomodulatory drugs designed to enhance and maintain the cancer-killing ability of CD8^+^ TILs is increasingly used in clinics for the treatment of advanced cancers such as metastatic melanoma and mismatch-repair deficient colorectal cancer.^6 7^ However, variable responses to anti-PD-1/PD-L1 therapy have been observed in immunotherapy trials, even in tumors with a high mutational burden, suggesting that additional mechanisms contribute to resistance.^8 9^ Factors such as loss of MHC class I expression, poor antigen presentation, T cell dysfunction, and local immunosuppression are known to facilitate immune escape in PD-1 refractory tumors.^4^ Furthermore, CD8^+^ T cell activation in response to tumors is a double-edged sword where strong antigenic stimulation can result in T cell anergy.^10^

CD8^+^ TILs are a highly diverse population with distinct phenotypic functions and specificities across patients and within individual tumors.^11^ Notably, CD8^+^ TILs include subpopulations displaying varying levels of functional exhaustion, including ‘precursor exhausted’ cells that can respond to PD-1 immune checkpoint blockade. Thus, melanoma patients with high levels of precursor exhausted TILs respond better to anti-PD-1 than patients with lower levels, or with high levels of terminally exhausted TILs.^12^ Approaches to expand the population of tumor-specific precursor exhausted T cells could be one way to improve the response to checkpoint blockade. The relationship between CD8^+^ specificities, tumor escape via antigen loss (immunoediting) and treatment resistance is unknown. Recent evidence has shown that T cell fine-tuning of specificity (even within the same tumor-associated antigen) may be crucial for determining clinical outcomes for naturally induced cytotoxic T cells and those elaborated after immune checkpoint blockade; and certain specificities are associated with functional T cell phenotypes that are protective in the settings of HBV-specific cytotoxic T cell response in hepatocellular carcinoma and OVA-specific cytotoxic T cell response in the KP mouse model.^13 14^ Further progress in this field of immunotherapy will provide greater insights on the relationship between T cell specificity, tumor immune escape and treatment resistance.

We have previously described the evolution of tumor-specific CD8^+^ T cell response in BALB/c mice 7–22 days after subcutaneous implantation of autologous CT26 colorectal tumors.^15^ CD8^+^ T cells of multiple specificities were primed in the tumor-draining lymph nodes (t-DLN), and as the tumor progressed, the ratio of effector to exhausted phenotypes detectable in the t-DLNs decreased. At the tumor site, the CD8^+^ T cell response largely focuses on two epitopes of the murine leukemia virus glycoprotein gp70 (AH1 and GSW11) which together account for 60%–90% of CD8^+^ TILs between days 14 and 22. A majority of these TILs coexpressed the exhaustion markers PD-1, Tim-3 and LAG-3 and were non-functional (IFNγ negative) to an extent that precluded the detection of GSW11-specific CD8^+^ T cells using intracellular cytokine staining, although some AH1-specific T cells were detectable. In this sense, therefore, the GSW11 response is cryptic. We found that the IFNγ response of GSW11-specific CD8^+^ T cells was revealed when CT26 was inoculated subcutaneously in Treg-depleted recipients, which correlated well with protection and involved the selective expansion of low avidity clonotypes.^15^ CT26 is a heavily utilized preclinical model in immuno-oncology studies and has been critical for the preclinical development of several PD-1 antibodies where numerous reports have shown it to be moderately responsive to anti-PD-1.^16 17^ Therefore, we set out to investigate the involvement of the GSW11-specific responses in this setting.

## Methods

### In vivo challenge and treatment strategy

Studies were compliant with the UK National Cancer Research Institute Guidelines for Animal Welfare in Cancer Research and the ARRIVE (Animal Research: Reporting of In Vivo Experiments) guidelines. We used the ARRIVE1 checklist when writing our report.^18^ CT26.WT is a murine colorectal carcinoma cell line induced by *N*-nitroso-*N*-methylrethane treatment in BALB/c mice and was commercially sourced from ATCC. Cells were maintained in RPMI-1640 (Sigma), supplemented with 10% fetal bovine serum (GlobePharm), 2 mM L-glutamine and 1X penicillin/streptomycin (Sigma-Aldrich) and confirmed as mycoplasma-free. BALB/c mice were inoculated subcutaneously at the right flank with 10^5^ CT26.WT cells in endotoxin-free phosphate-buffered saline (PBS). Mice were treated with either 200 µg PD-1 mAb (RMP1-14, rat IgG2a, Bio X Cell) or PBS intraperitoneally on days 3, 6 and 9 after tumor implantation. Tumor growth was monitored from day 3 using caliper measurements. Only mice with palpable tumors were included in the experiments and subsequent analyses. Tumor progression or regression of anti-PD-1 treatment was determined using the Response Evaluation in Early Tumors (REET) based on a Tumor Control Index criterion.^19^ Please see online supplemental materials for further details.

10.1136/jitc-2023-007114.supp5 Supplementary data

### Tissue processing and flow cytometry

Tumors from CT26.WT tumor-bearing mice were harvested between days 10 and 12. Single-cell suspensions were prepared from the tumors using a gentleMACS Tumor Dissociation Kit (Miltenyi Biotec) and 40 µm cell strainers (Falcon, Thermo Fisher Scientific, USA) according to the manufacturer’s instructions. CD8^+^ T cell responses to AH1 (SPSYVYHQF; GenScript) or GSW11 (GGPESFYCASW; GenScript) were assessed using AH1 MHC dextramers (Immudex) and GSW11-specific tetramers (in-house),^15^ respectively, and IFN-γ production following peptide stimulation. CD8^+^ T cells, APCs and peptides were cultured together in the presence of brefeldin A (BD Biosciences) for 4 hours at 37°C. For further functional characterization of TILs, cells were harvested and washed twice before being incubated with an FcγR block (2.4G2; BD Biosciences) for 10 min at RT and stained with GSW11-specific tetramers for 30 min at 37°C, washed thrice and stained for the cell surface markers: CD3 (17A2, BioLegend); CD8 (63-6.7; BD Biosciences), PD-1 (RMPI-30; eBioscience), CD39 (24DMS1; eBioscience), and fixable viability dye for dead cells discrimination (Invitrogen) for 30 min on ice. The cells were fixed and permeabilized using the Cytofix/Cytoperm kit (BD Biosciences) before intracellular staining for the expression of: IFNγ (XMG1.2; BD Biosciences), Granzyme B (REA226, Miltenyi Biotec), T-bet (4B10; BioLegend), Eomes (W17001A, BioLegend) and Tcf-1 (S33-966, BD Biosciences). Flow cytometry was performed on an LSRFortessa (BD Biosciences), with appropriate lasers and filters, unstained and single-stained controls for compensation. Data were analyzed using FlowJo software (Tree Star, BD Biosciences).

### T cell receptor clonality

To assess the TCR clonality of GSW11-specific T cells that were already primed in the t-dLNs, we used a panel of 15 Vβ-specific antibodies (BD Biosciences). First, t-DLNs were harvested from anti-PD-1 treated mice with progressing versus regressing tumors at the study endpoint. Total CD8^+^ T cells were purified using CD8 magnetic-activated cell sorting based on negative selection (Miltenyi Biotec) according to the manufacturer’s instructions. Purified CD8^+^ T cells were stained with anti-CD8, GSW11-specific tetramers, and a Vβ-specific antibodies kit, followed by flow cytometry.

### In vitro and in vivo T cell cytotoxicity

Tumors and t-DLNs were harvested from anti-PD-1 treated mice (progressors and regressors) between days 10 and 12 to obtain single cell suspensions. Cells were stained with anti-CD8 and GSW11-specific tetramers for FACS sorting of Tet^hi^ and Tet^lo^ GSW11-specific CD8^+^ T cells (BD FACS Aria II) for in vitro and in vivo T cell cytotoxicity evaluation. For the in vitro T cell cytotoxicity assay, CT26 cells were stained with 3 µM 5,6-carboxyfluorescein diacetate succinimidyl ester (CFSE; Sigma-Aldrich), and cocultured with Tet^hi^ (pooled from five progressors) or Tet^lo^ (pooled from five regressors) GSW11-specific T cells separately at a 10:1 ratio of CFSE-labeled target cells to effector T cells at 37°C for 48 hours in the presence of 10 µM GSW11 peptide and 20 U/mL recombinant IL-2 (PeproTech). The cells were then stained with annexin V and propidium iodide and analyzed using flow cytometry to evaluate early and late apoptosis. In a separate experiment to determine cell death caused by T cell cytotoxicity, CT26 cells were stained with PKH26 (Sigma-Aldrich) according to the manufacturer’s instructions and incubated with Tet^hi^ and Tet^lo^ GSW11-specific T cells separately at a 10:1 ratio at 37°C for 4 hours. The cells were then stained with ToPro-3 iodide (Molecular Probes, Invitrogen) and analyzed by flow cytometry. To investigate T cell cytotoxicity in vivo, Tet^hi^ or Tet^lo^ GSW11-specific CD8^+^ T cells were adoptively transferred into CT26 tumor-bearing mice on day 1 postimplantation, with additional anti-PD-1 treatment on days 3 and 9 in a separate study. The treatment efficacy was determined based on tumor growth and survival monitoring until the study endpoint.

### RNA-seq and bioinformatics analysis

Tet^hi^ and Tet^lo^ GSW11-specific CD8^+^ T cells were sorted by FACS from regressing and progressing tumors and harvested into TRIzol reagent for bulk RNA-seq. Full-length libraries were prepared using the Smart-seq2 protocol as described by Picelli *et al*.^20^ Please see online supplemental materials for further details.

### Tetramer generation and tetramer competition assay

GSW11-specific tetramers based on a class I peptide-MHC single-chain trimer (SCT) construct containing H2-D^d^, β2m, and GSW11 peptide were generated according to.^15^ t-DLNs were harvested between days 10 and 12 from anti-PD-1 treated mice for the tetramer competition assay. CD8^+^ T cells were purified from single-cell suspensions via magnetic isolation using negative selection (Miltenyi Biotec). Purified CD8^+^ T cells were incubated with 50 nM dasatinib (Selleck Chemicals) to prevent TCR internalization before staining with anti-CD8, anti-TCR β-chain (H57-597; Biolegend) and 5 µg of PE-labeled GSW11-specific tetramers. After two washes, the cells were incubated with bleached tetramers at varying ratios of initial PE-labeled tetramers: 2.5, 5, 10 or 20 µg per test. The bleached tetramers were tested for no/minimal PE-fluorescence prior to use. TCR β-chain staining was performed to ensure that the decreasing levels of PE staining were due to outcompetition of fluorescently labeled tetramers and not due to TCR internalization.

### Biophysical measurement of T cell avidity

T cell avidity based on acoustic force spectroscopy and microfluidic lab-on-chip image-based tracking of labeled T cells was used for direct biophysical measurement of the binding interactions or force between effector T cells and target CT26 cells (Z-Movi Cell Avidity Analyzer, LUMICKS). Avidity measurements were performed on CD8^+^ T cells isolated from the t-dLNs of individual mice (regressors or progressors) following anti-PD-1 treatment. Please see online supplemental materials for further details.

### Statistical analysis

Analyses were performed using Prism (GraphPad Software, USA). The p values were calculated using either two-way analysis of variance with Dunnett’s post hoc test or two-tailed unpaired t-test (*p≤0.05; ***p≤0.001; ****p≤0.0001).

## Results

### Therapeutic response to anti-PD-1 is associated with a subpopulation of CD8^+^ T cells recognizing the subdominant tumor-derived epitope GSW11

**Figure 1:**
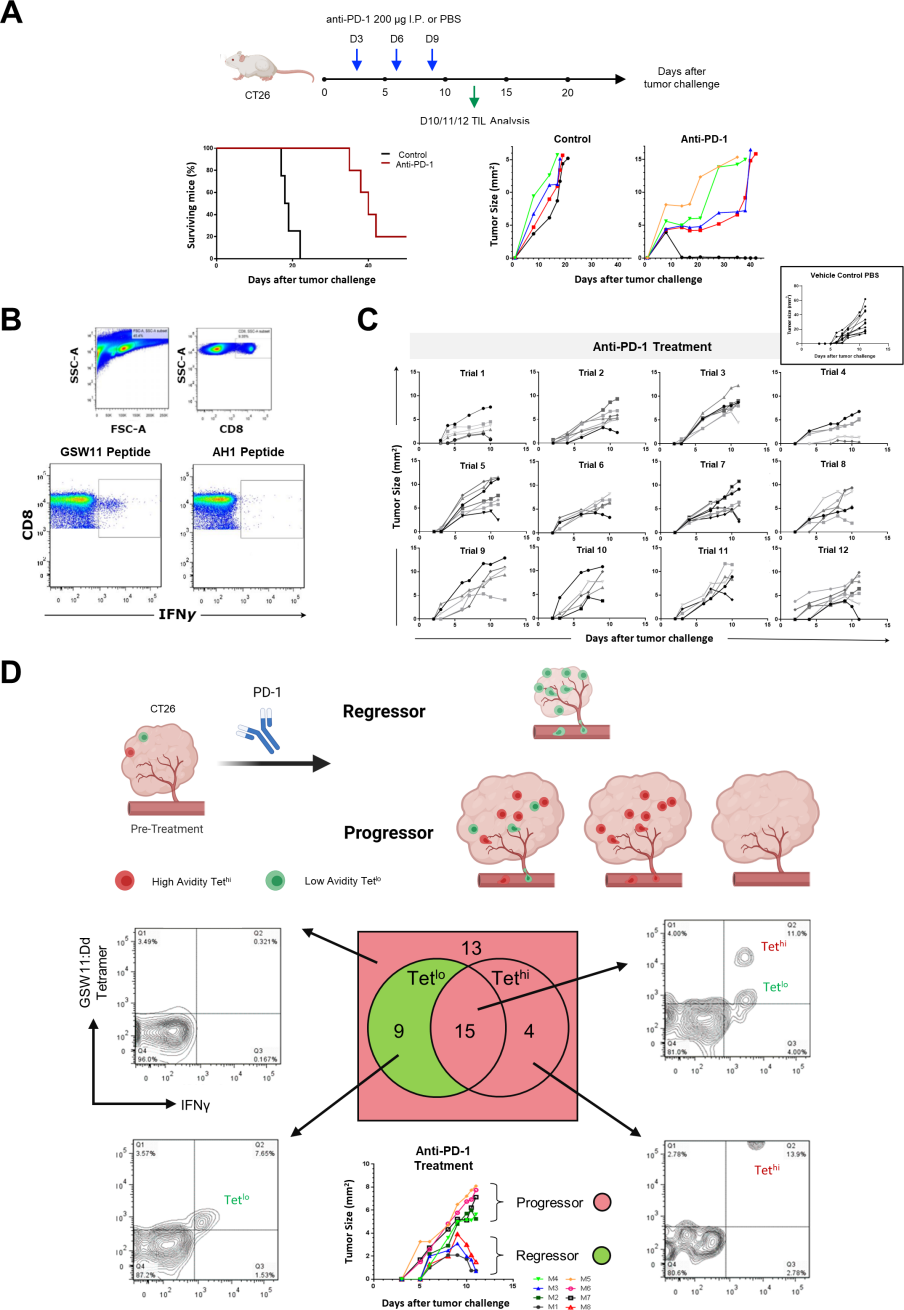
Therapeutic response to anti-PD-1 is associated with a subpopulation of GSW11-specific CD8^+^ T cells with low TCR avidity. (A) BALB/c CT26 mouse colorectal cancer models were treated with either 200 µg of anti-PD-1 or PBS control on days 3, 6 and 9 post-tumor implantations. This was followed by immuno-profiling of the tumor-infiltrating lymphocytes (TIL) between days 10 and 12. Kaplan-Meier plot and tumor growth curves representing the efficacy of anti-PD-1 based on the treatment regimen are shown. (B) Flow cytometry plots demonstrating the differential expression of interferon gamma (IFNγ) between GSW11-specific and AH1-specific CD8^+^ T cells isolated from t-dLNs on day 7 following rechallenge. (C) CT26 tumor growth curve from days 0 to 12 post-tumor implantation for 70 mice from 12 separate anti-PD-1 treatment trials performed over a period of 24 months. Each plot represents mice from each trial. A plot of tumor growth kinetics for 12 untreated mice (given PBS as vehicle control) is seen in the top right hand corner. (D) Graphical representation and Venn diagram on the distribution of low avidity (Tet^lo^) and high avidity (Tet^hi^) GSW11-specific CD8^+^ TILs in the regressors and progressors following anti-PD-1 treatment, the associated flow cytometry plots for GSW11:Dd tetramer and IFNγ staining and tumor growth curves of mice determined to be regressors or progressors based on REET score. REET, Response Evaluation in Early Tumors.

The schedule adopted in this study involved three intraperitoneal administrations of anti-PD-1 on days 3, 6, and 9 following the subcutaneous implantation of CT26 tumors, which resulted in a variable impact on the tumor growth rate during early tumor development and a 20%–30% curative response rate (figure 1A). When the surviving mice were rechallenged with a second inoculum of CT26 and t-dLNs were harvested on day 7, the IFNγ response of GSW11-specific CD8^+^ TILs were consistently stronger than that of AH1-specific CD8^+^ T cell response (figure 1B). This finding agrees with previous observations that the expansion of a functional GSW11-specific response correlates with treatment efficacy in other therapeutic settings.^15 21^

Next, we investigated the specificity and functional status of CD8^+^ TILs during anti-PD-1 therapy. To evaluate the treatment response in early tumors, we adopted an experimental endpoint based on the daily evaluation of response to treatment from days 0 to 12 post-tumor implantation. Tumors that progressed on treatment were assigned a REET score of 0, whereas tumors that regressed by less than or greater than 10% since the last measurement point were assigned REET scores of 1 and 2, respectively. The experimental endpoint was reached when the cumulative REET score for any individual mouse in the experimental group reached 3 (when the tumor size decreased by more than 10% per day for two consecutive days). We classified 70 tumors, from 12 separate anti-PD-1 immune checkpoint inhibition immunotherapy experiments performed over a period of 24 months (figure 1C and online supplemental table 1).

10.1136/jitc-2023-007114.supp1 Supplementary data

All tumors were analyzed for the frequency of tumor-infiltrating GSW11-specific CD8^+^ T cells by using in-house fluorescent tetramers of GSW11:Dd SCT.^15^ We found that tumors scoring positive for GSW11-specific CD8^+^ TILs often had more than one population present, distinguishable by the level of tetramer staining (figure 1D), and when tumors containing GSW11-specific CD8^+^ TILs with high tetramer staining (Tet^hi^) were omitted from the analysis—leaving those tumors with only a tetramer-low (Tet^lo^) response—the correlation with tumor regression was significantly improved. In fact, 9/9 regressing tumors, and 0/32 progressing tumors contained only the Tet^lo^ GSW11-specific CD8^+^ TILs (figure 1D). These data are consistent with the relative intensity of the IFNγ response following secondary challenge (figure 1B).

### GSW11-specific CD8^+^ T cells associated with therapeutic response to anti-PD-1 are low avidity and have a distinct TCR Vβ clonal distribution

**Figure 2:**
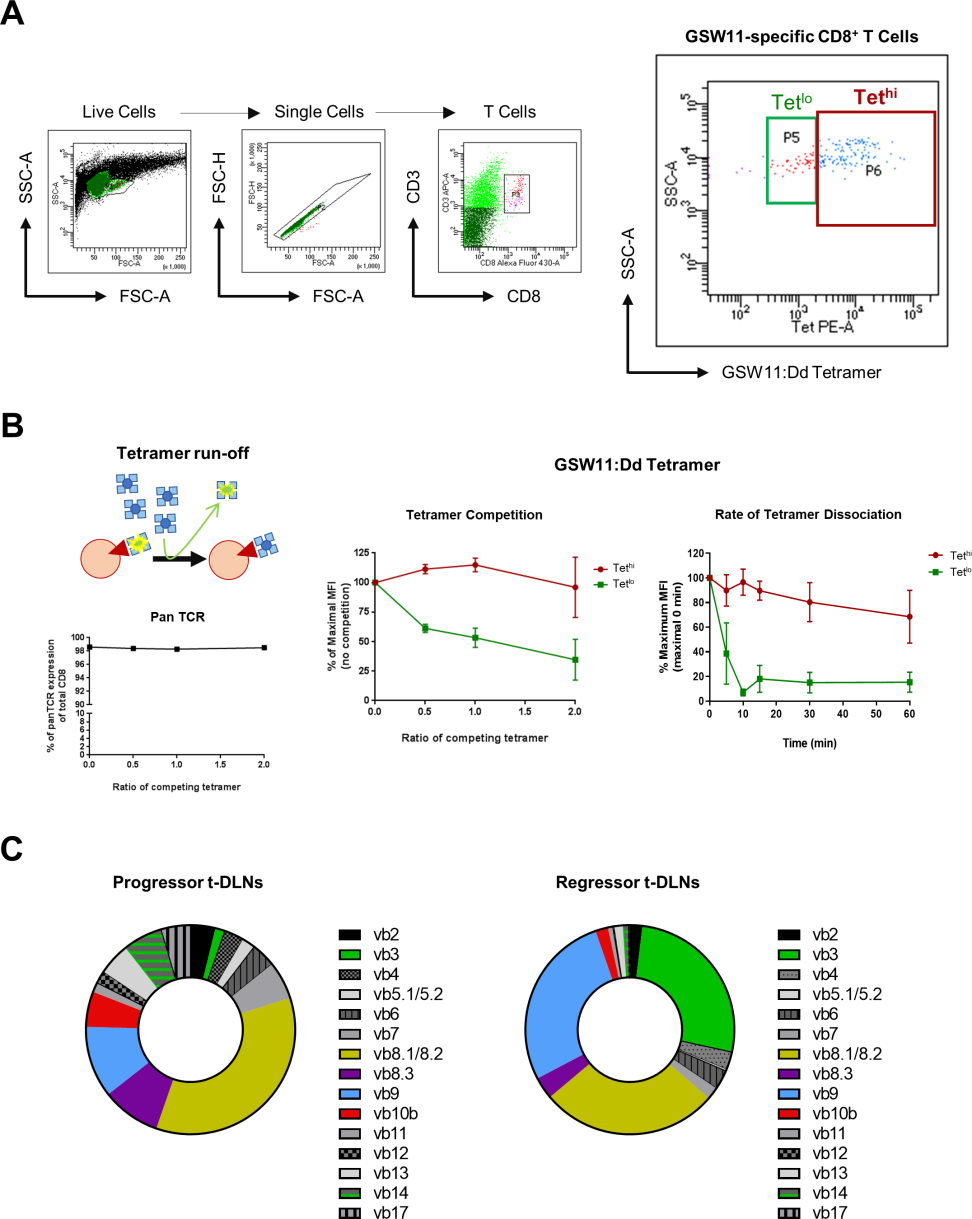
Low avidity GSW11-specific CD8^+^ T cells and oligoclonal expansion of TCR Vβ3 and Vβ9 clonotypes. (A) Representative flow cytometry plots on the gating strategy for tetramer-sorting of high avidity (Tet^hi^) and low avidity (Tet^lo^) GSW11-specific CD8^+^ TILs from t-dLNs. (B) Percentage pan-TCR expression of total CD8^+^ TILs with increasing concentration of competing GSW11:Dd tetramers, IC50 of Tet^hi^ and Tet^lo^ GSW11-specific CD8^+^ TILs and the rate of tetramer dissociation over 60 min. (C) TCR Vβ clonal distribution of GSW11-specific CD8^+^ TILs from the t-dLNs of progressors and regressors following anti-PD-1 treatment. t-dLNs, tumor-draining lymph nodes; TILs, tumor-infiltrating lymphocytes.

A good correlation has been described between the level of tetramer staining and TCR avidity in T cells with equal levels of TCR expression.^22^ Therefore, we isolated Tet^hi^ and Tet^lo^ GSW11-specific CD8^+^ T cells from t-DLNs to estimate the average relative TCR avidities of the sorted populations using a tetramer competition assay.^15^ Figure 2A shows the gating strategy for Tet^hi^ and Tet^lo^. Although both populations expressed the same level of TCR, the Tet^lo^ sorted population had a lower IC50 than Tet^hi^, indicating lower avidity. This was confirmed by measuring the rate of tetramer dissociation over 60 min (figure 2B). Accordingly, the t1/2 value for Tet^hi^ and Tet^lo^ GSW11-specific CD8^+^ T cells are >70 hours and 2.3 hours, respectively. Furthermore, the presence of lower avidity CD8^+^ T cells in the t-DLNs of regressors compared with progressors treated with anti-PD-1 was confirmed by direct biophysical measurement of cellular avidity based on acoustic force spectroscopy and microfluidic lab-on-chip fluorescent tracking of labeled T cell interactions with target CT26 cells (online supplemental figure 1). Next, we determined the diversity of TCR repertoires of GSW11-specific T cells from t-DLNs of treated mice with progressing or regressing tumors using a panel of TCR Vβ-specific mAbs. The anti-GSW11 response was distinct between progressors and regressors, with at least 15 different clonotypes observed. Two TCR clonotypes, Vβ3 and Vβ9, were expanded among GSW11-specific CD8^+^ TILs from the regressors (figure 2C), indicating an anti-PD-1 induced oligoclonal expansion correlating with the therapeutic response. Interestingly, we have previously observed preferential expansion of low avidity Vβ3 expressing clonotypes that correlate with curative responses in a Treg depletion model of CT26.^15^

### Low avidity GSW11-specific CD8^+^ T cells have a less exhausted phenotype compared with their high avidity counterparts

**Figure 3:**
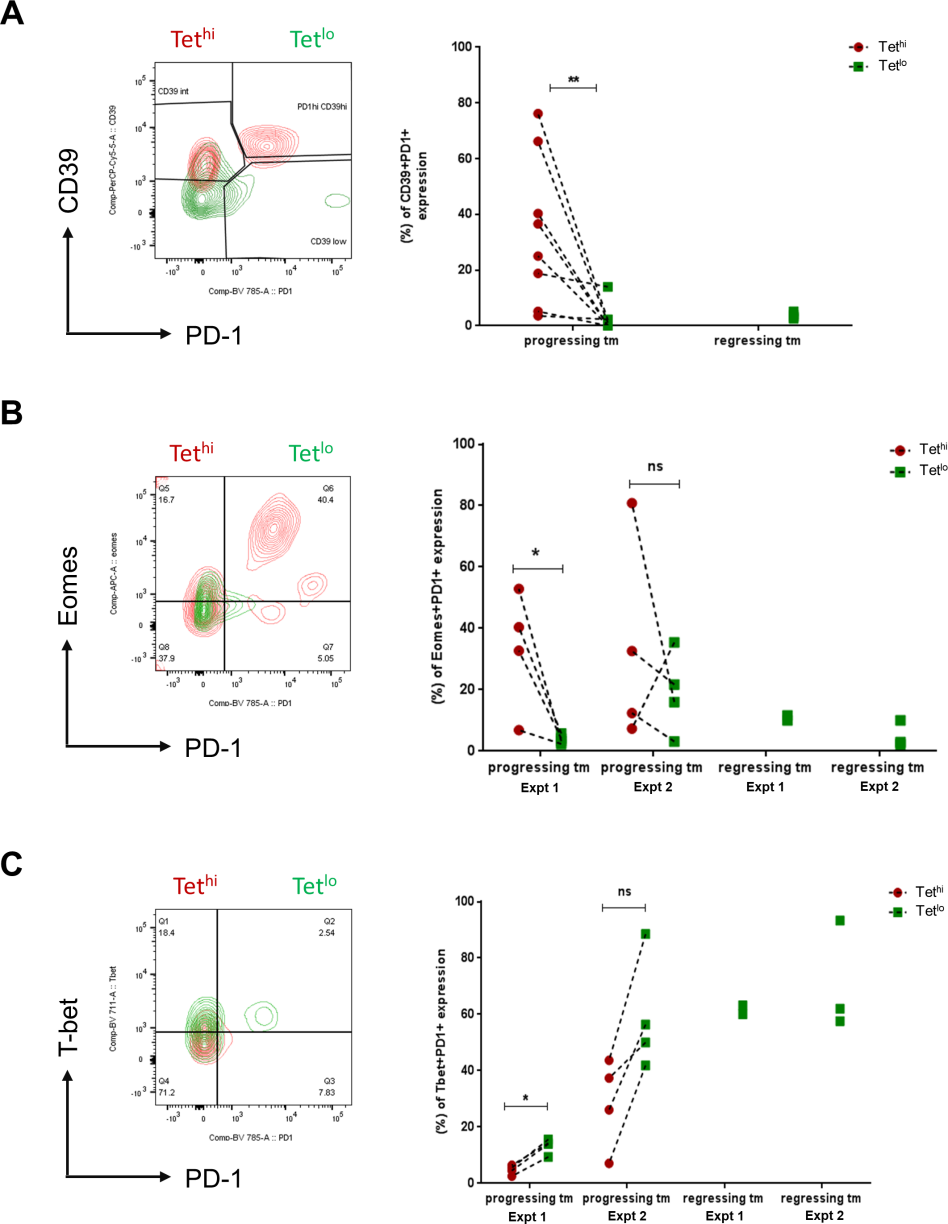
Immuno-profiling of T cell exhaustion markers showed a less exhausted phenotype in low avidity GSW11-specific CD8^+^ T cells compared with their high avidity counterparts. Contour plots and percentage coexpression of (A) CD39 and PD-1; (B) Eomes and PD-1; and (C) T-bet and PD-1 on Tet^hi^ and Tet^lo^ GSW11-specific CD8^+^ TILs isolated from the progressing and regressing tumors (tm) following anti-PD-1 therapy. Tet^hi^ and Tet^lo^ populations are color coded as red and green, respectively. TILs, tumor-infiltrating lymphocytes.

Our observation that the exclusive presence of tumor-infiltrating low avidity GSW11-specific CD8^+^ T cells was predictive of tumor regression suggests that there may be a difference in the functional phenotype between high and low avidity T cells recognizing the same epitope. Therefore, we sorted Tet^hi^ and Tet^lo^ cells from both progressing and regressing tumors and investigated their expression of cell-surface receptors associated with T cell differentiation from a progenitor to an exhausted cell state. We found that Tet^hi^CD8^+^CD44^+^ T cells coexpressed significantly higher levels of CD39 and PD-1 than Tet^lo^ (figure 3A), higher levels of both PD-1 and Eomes (figure 3B), and lower levels of T-bet (figure 3C). Taken together, these results suggest that GSW11-stimulated T cells bearing higher avidity TCR are more likely to be clonally exhausted than their low avidity counterparts. Tet^lo^ had a similar phenotype regardless of whether they were isolated from regressing tumors in which they were the only GSW11-specific response or coexisted with a Tet^hi^ response in progressing tumors.

**Figure 4:**
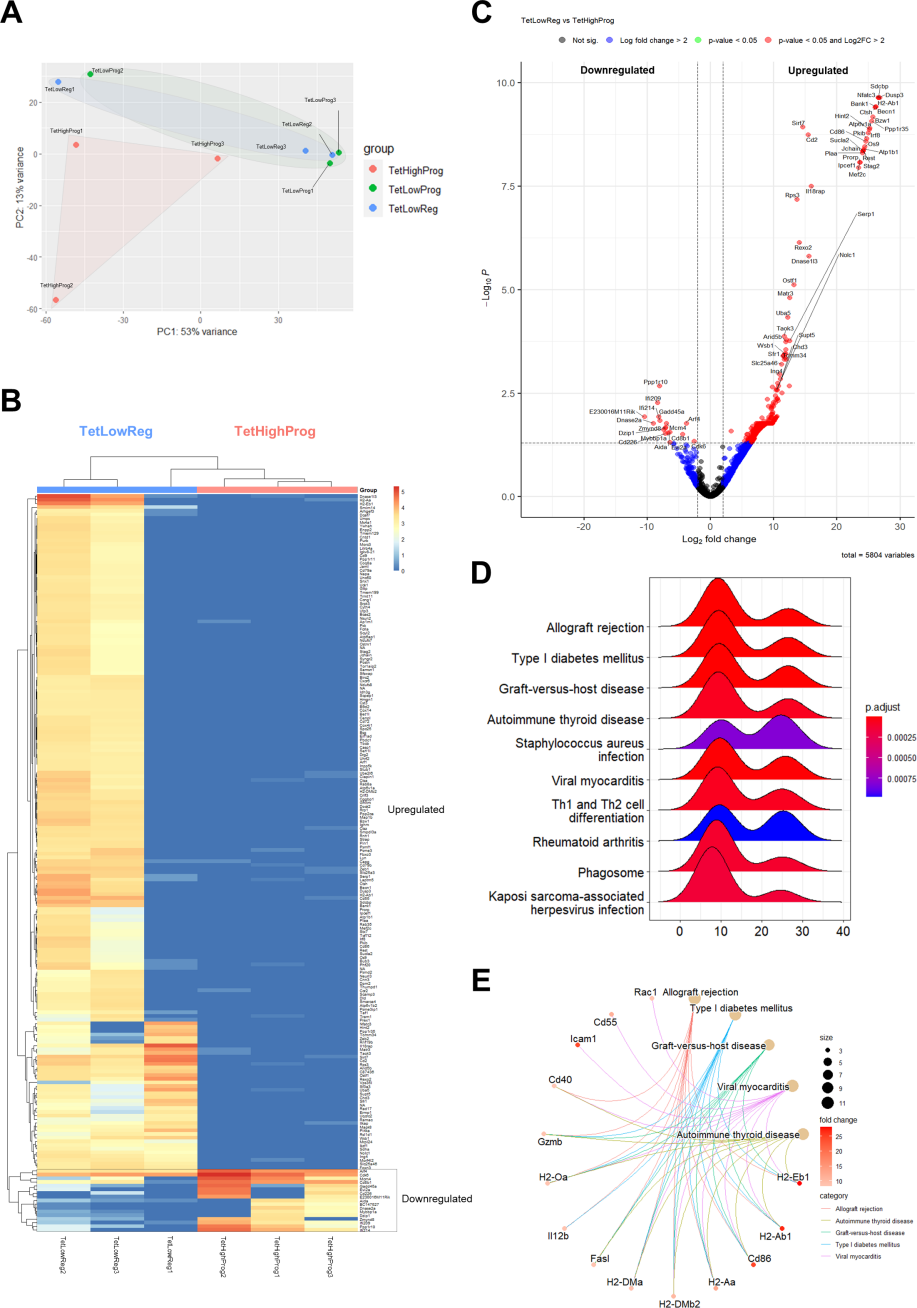
Transcriptomic analysis showed upregulated genes related to antigen presentation, TCR signaling, T cell cytotoxicity and oxidative phosphorylation and downregulated transcripts associated with DNA damage, apoptosis, and autophagy in low avidity GSW11-specific CD8^+^ T cells isolated from regressing tumors. (A) Principal component analysis (PCA) plot of GSW11-specific CD8^+^ T cell subsets isolated from the regressing and progressing tumors. The degree of similarity or variation in transcriptomic profiles between samples from the three T cell subsets are shown: Tet^hi^ GSW11-specific CD8^+^ T cells from progressing tumors (TetHighProg), Tet^lo^ GSW11-specific CD8^+^ T cells from progressing tumors (TetLowProg) and regressing tumors (TetLowReg). (B) Unsupervised hierarchical clustering and heatmap of the normalized gene counts of the top 200 differentially expressed genes in TetLowReg versus TetHighProg. (C) Volcano plot of fold change (FC) versus p value of all differentially expressed genes in TetLowReg versus TetHighProg. Intersecting lines indicate the log_2_FC cut-off. Colors as indicated; black, NS; blue, log_2_FC>2; green, p<0.05; red, p<0.05 and log_2_FC>2. (D) Ridgeplot of significantly enrichment KEGG pathways in TetLowReg versus TetHighProg (adjusted p<0.05). (E) Cnetplot showing the linkage of enriched KEGG pathways in TetLowReg and shared genes. The scalebars of the node size reflect the number of significantly enriched genes in the node and fold change indicates the level of gene expression.

To further investigate the phenotypic differences between high and low avidity tumor-specific CD8^+^ TILs, we performed bulk RNA-seq on tetramer sorted GSW11-specific T cells from three regressing and three progressing CT26 tumors: giving three groups of total Tet^hi^CD8^+^CD44^+^ TILs from the progressing tumors (TetHighProg), Tet^lo^CD8^+^CD44^+^ TILs from the progressing tumors (TetLowProg) and Tet^lo^CD8^+^CD44^+^ TILs from the regressing tumors (TetLowReg). The workflow for RNA-seq analysis is found in online supplemental figure 2. Principal component analysis showed heterogeneity in the transcriptional profiles of samples from all three groups, with TetLowReg clustering with TetLowProg, and TetHighProg (or Tet^hi^) exhibiting greater variation from the two Tet^lo^ groups (figure 4A). Comparison of the transcriptional profiles of TetLowReg versus TetHighProg using DESeq2 identified 1054 differentially expressed genes (DEGs) that were significantly upregulated and 17 genes that were significantly downregulated in TetLowReg (p<0.05, log_2_ fold change>2), represented by hierarchical clustering in the heatmap (figure 4B) and volcano plot (figure 4C). The numbers and full list of DEGs are shown in online supplemental table 2. Some of the upregulated transcripts with known functions in T cell biology include genes encoding the nuclear factor of activated T cell 3 (NFATc3) which has been shown to regulate IL-2 and COX-2 gene expression for T cell activation and proliferation^23^; interferon regulatory factor 8 (IRF8) which is important for integrating TCR and cytokine signaling pathways to drive effector CD8^+^ T cell differentiation^24^; CD2, a costimulatory receptor that binds to LFA-3 and plays crucial roles in antigen presentation and T cell activation, and is known to correlate negatively with exhaustion in human CD8^+^ TILs^25^; C-X-C motif chemokine receptor 6 (CXCR6), which is associated with the magnitude and outcome of T cell anti-tumor response, tissue retention of memory T cells, as well as the survival and local expansion of effector T cells in tumors^26 27^; and Sirtuin 7 (Sirt7), which belongs to a family of NAD^+^-dependent histone deacetylases involved in epigenetic modulation of cell cycle progression, metabolic homeostasis, stress resistance and T cell activation.^28^ The transcription factor 7-like 2 protein (Tcf7l2) which belongs to the T cell factor (TCF) family of high-mobility group box transcription factors and is a major effector of the canonical Wnt signaling pathway was also differentially upregulated in Tet^lo^. Tcf7l2 is known to play crucial roles in the regulation of cell proliferation and maintenance of stemness in embryonic tissues and adult stem cells and has been implicated in the regeneration of hematopoietic lineages.^29 30^ It has been shown to expand in response to checkpoint blockade, leading to a cytotoxic effector response. Downregulated transcripts include genes encoding protein phosphatase 1 regulatory subunit 10 (PPP1R10), which plays a role in many cellular processes including cell cycle progression, DNA repair, and apoptosis by regulating the activity of protein phosphatase 1^31^; growth arrest and DNA damage-inducible protein 45 (GADD45), which is a negative regulator of activation-induced T cell proliferation involved in autoimmunity^32^; and Dnase2a, which is a lysosomal DNA endonuclease important for the degradation and clearance of damaged nuclear DNA via autophagy.^33^

10.1136/jitc-2023-007114.supp2 Supplementary data

To explore this transcriptional heterogeneity in greater detail, we performed gene set enrichment analysis (GSEA) using KEGG, GO as well as predefined gene sets in MSigDB. GSEA revealed 29 significantly enriched KEGG pathways in Tet^lo^ GSW11-specific T cells isolated from regressing tumors. figure 4D shows the top 10 significantly enriched KEGG pathways in TetLowReg (compared with TetHighProg). Most of these pathways are related to tissue-destructive pathogenic conditions in infections and autoimmune disorders, such as allograft rejection, type I diabetes mellitus, graft-versus-host disease, viral myocarditis, and autoimmune thyroid disease. Further analysis on the shared transcripts between the enriched KEGG pathways (figure 4E) showed the upregulation of genes associated with the ‘immunologic constant of rejection’,^34^ such as those involved in T cell cytotoxicity, for example, granzyme B (Gzmb) and the tumor necrosis factor receptor superfamily member 6 (tnfrsf6 or Fasl),^35 36^ T cell cytoskeletal remodeling, polarization and migration such as the small Rho GTPase Rac1,^37^ endothelial transmigration and cellular interactions with antigen-presenting cells, for example, intercellular adhesion molecule 1 (Icam1), T cell activation, for example, interleukin 12b (il12b), T cell costimulation and effector memory, for example, cd86,^38^ well as several class II molecules that could be induced by IFNγ signaling^39 40^ and are a marker for T cell activation.^41^ These results were further confirmed by GSEA analysis using the MSigDB C5 (Molecular Function) ontology gene set, which showed an enrichment of pathways related to antigen-binding, immune receptor activity, and signaling receptor binding (online supplemental figure 3).

When we classified the same set of genes comparing TetLowReg to TetHighProg using REACTOME pathway analysis, we found a significant enrichment in biological processes related to cell cycle checkpoints (enrichment score: 0.67, p adj. value: 4.20E-09), cell survival such as the TNFR2 non-canonical NF-κB pathway (enrichment score: 0.55, p adj. value: 1.48E-06), TCR signaling (enrichment score: 0.52, p adj. value: 2.55E-06), signaling by interleukins (enrichment score: 0.32, p adj. value: 8.50E-05), interferon signaling (enrichment score: 0.38, p adj. value: 4.26E-04), regulation of apoptosis (enrichment score: 0.67, p adj. value: 2.08E-06), DNA double-strand break repair (enrichment score: 0.49, p adj. value: 6.63E-07), cellular response to hypoxia (enrichment score: 0.56, p adj. value: 2.37E-05), glycolysis (enrichment score: 0.56, p adj. value: 4.37E-05) as well as TCF-dependent signaling in response to Wnt (enrichment score: 0.32, p adj. value: 0.03). A full list of enriched REACTOME pathways is provided in online supplemental table 3. Therefore, pathways that were preferentially activated in TetLowReg suggest a population of T cells that were clonally expanding, and optimized for survival, response to and secretion of inflammatory cytokines.

10.1136/jitc-2023-007114.supp3 Supplementary data

### Low avidity T cells from progressing tumors have a similar phenotype to low avidity T cells from regressing tumors

DESeq2 analysis of TetLowProg compared with TetLowReg showed only minor differences between the two populations (online supplemental figure 4). In general, regulatory genes related to hypoxia and metabolic reprogramming such as the DNA-binding transcriptional adaptor 2A (Tada2a) and the HIF1α-regulated angiogenic growth factor canopy FGF signaling regulator 2 (Cnyp2) were upregulated in TetLowProg and branched chain keto acid dehydrogenase E1 subunit beta (Bckdhb) and immunoglobulin kappa variable 1–135 (Igkv1-135) were downregulated. GO analysis showed the enrichment of genes associated with non-coding RNA metabolic processes in TetLowProg. However, the Tet^lo^ population found in progressing tumors is still functional to some extent, as GSEA analysis using the MSigDB Hallmark gene set collection confirmed similarities between TetLowReg and TetLowProg which are both enriched for gene sets related to allograft rejection, oxidative phosphorylation, and DNA repair (online supplemental figure 5). Enrichment analysis using MSigDb C7 immunologic signatures showed that the TetLowProg population was enriched for gene sets associated with acute (Armstrong strain) versus chronic (clone 13) LCMV infection in mice, although less significantly than TetLowReg. Together with the PD-1, CD39, Eomes and T-bet expression data, as well as the higher expression of Tcf-1 in Tet^lo^ GSW11-specific TILs compared with Tet^hi^ population, the gene expression profiles of Tet^lo^CD8^+^CD44^+^ GSW11-specific T cells were suggestive of ‘precursor exhausted’ or ‘progenitor’ versus ‘terminally exhausted’ T cells that have recently been associated with differential control of tumors and response to anti-PD-1 checkpoint blockade.^12^

Our functional analyses based on flow cytometry and bulk RNA-seq analysis showed that Tet^lo^ from both regressing and progressing tumors was phenotypically similar (‘precursor exhausted’). To further understand the differences observed in distinct Tet^lo^ subsets of precursor exhausted T cells found in regressing versus progressing tumors treated with anti-PD-1, we performed a GO analysis comparing TetLowReg and TetLowProg to TetHighProg. Significant enrichment of gene sets associated with response to cytokines, immune response, lymphocyte activation, proliferation, differentiation, migration, leukocyte cell-cell adhesion and immune effector processes consistent with a more active T cell response was found in TetLowReg compared with TetHighProg (online supplemental figure 6). Several pathways related to carbohydrate, lipid, ribonucleotide, and nucleoside phosphate metabolism were upregulated in TetLowReg compared with TetHighProg. In contrast, the transcripts of TetLowProg (compared with TetHighProg) were enriched in GO terms associated with leukocyte migration, regulation of cellular localization, biological processes related to protein transport and degradation, regulation of ion transport, negative regulation of cell communication and signaling, and autophagy. ATP metabolism and catabolic pathways involved in generating alternative sources of energy currency were more upregulated in TetLowProg than in TetHighProg, suggesting Tet^lo^ metabolic reprogramming in progressing tumors. Despite similarities in the precursor exhausted phenotype, differences in the transcriptome suggest that metabolic pressure within the progressing tumors and the presence of dysfunctional Tet^hi^ GSW11-specific TILs with higher CD39 and PD-1 expression or competition for binding to anti-PD-1, may have contributed to the metabolic adaptation and immunosuppression of Tet^lo^.

### Low avidity GSW11-specific CD8^+^ T cells exhibit greater cytotoxic function in vitro and in vivo

**Figure 5:**
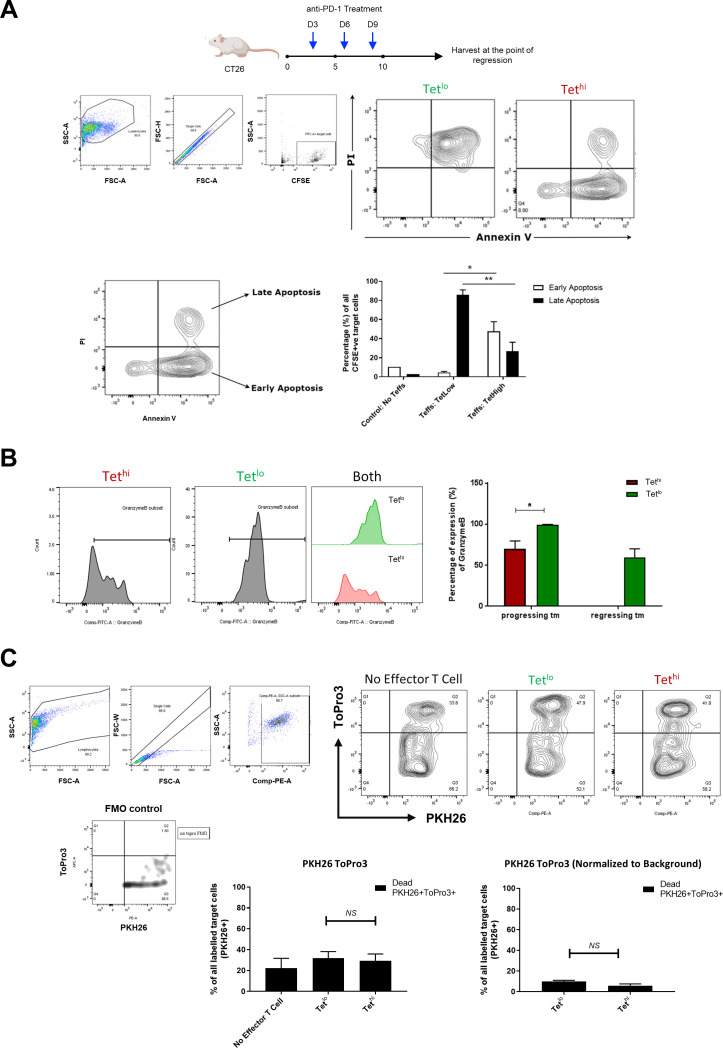
In vitro T cell cytotoxicity studies showed greater potency in killing of CT26 cells by low avidity GSW11-specific CD8^+^ T cells. (A) Tet^hi^ and Tet^lo^ GSW11-specific CD8^+^ T cells were FACS sorted from t-dLNs of anti-PD-1 treated mice at the point of regression. The T cell subpopulations were cocultured with CFSE-labeled CT26 cells in an effector to target ratio of 1:10. Killing was scored based on propidium iodide (PI) and annexin V staining, where CT26 cells which are positive for annexin V but negative for PI are in the phase of early apoptosis, while cells positive for both annexin V and PI are in late apoptosis. Contour plots and bar plots show differences in expression of early and late apoptosis markers between CT26 cells cocultured with Tet^hi^ and Tet^lo^ GSW11-specific CD8^+^ T cells. (B) Histograms and percentage Granzyme B expression on Tet^hi^ and Tet^lo^ GSW11-specific CD8^+^ T cells isolated from the progressing and regressing tumors (tm). (C) PKH26 and ToPro3 staining was conducted to determine very early apoptosis of CT26 following T cell coculture and cytotoxic killing. Contour plots and bar plots show the PKH26 and ToPro3 staining of CT26 cells cocultured with Tet^hi^ or Tet^lo^. CFSE, 5,6-carboxyfluorescein diacetate succinimidyl ester; t-dLNs, tumor-draining lymph nodes.

Next, we compared the cytotoxic potential of Tet^hi^ and Tet^lo^ GSW11-specific CD8^+^ T cells by coculturing tetramer-sorted T cells with CT26 cells before staining with annexin V and propidium iodide. Killing was scored as the fraction of all the targets that were positive for both markers. Figure 5A shows that Tet^lo^ cells were significantly more potent cytotoxic T cells than Tet^hi^, killing more than 80% of targets compared with 25%. Interestingly, over 40% of the recovered targets cocultured with Tet^hi^ cells were positive for annexin V staining but negative for propidium iodide staining, suggesting that they had entered a phase of early apoptosis. This phase of apoptosis is reversible and precedes commitment to cell death. Therefore, it is possible that Tet^hi^ has suboptimal cytotoxic function characterized by sublytic granule formation. To determine whether the observed differences in cytotoxic capacity was due to lower lytic granule expression, we performed Granzyme B (GzB) staining on Tet^hi^ and Tet^lo^ GSW11-specific CD8^+^ T cells isolated from progressing and regressing CT26 tumors (figure 5B). Both Tet^hi^ and Tet^lo^ were found to express GzB, consistent with both populations inducing early apoptosis. In progressors, more Tet^lo^ were found to be GzB-positive, and these are the subpopulations selected for the adoptive transfer experiments. Thus, these suggested that GzB is not the limiting factor for therapeutic efficacy. Consistent with this, when we measured very early apoptosis using ToPro-3 iodide influx, we found similar levels of activity between Tet^hi^ and Tet^lo^ (figure 5C).

**Figure 6:**
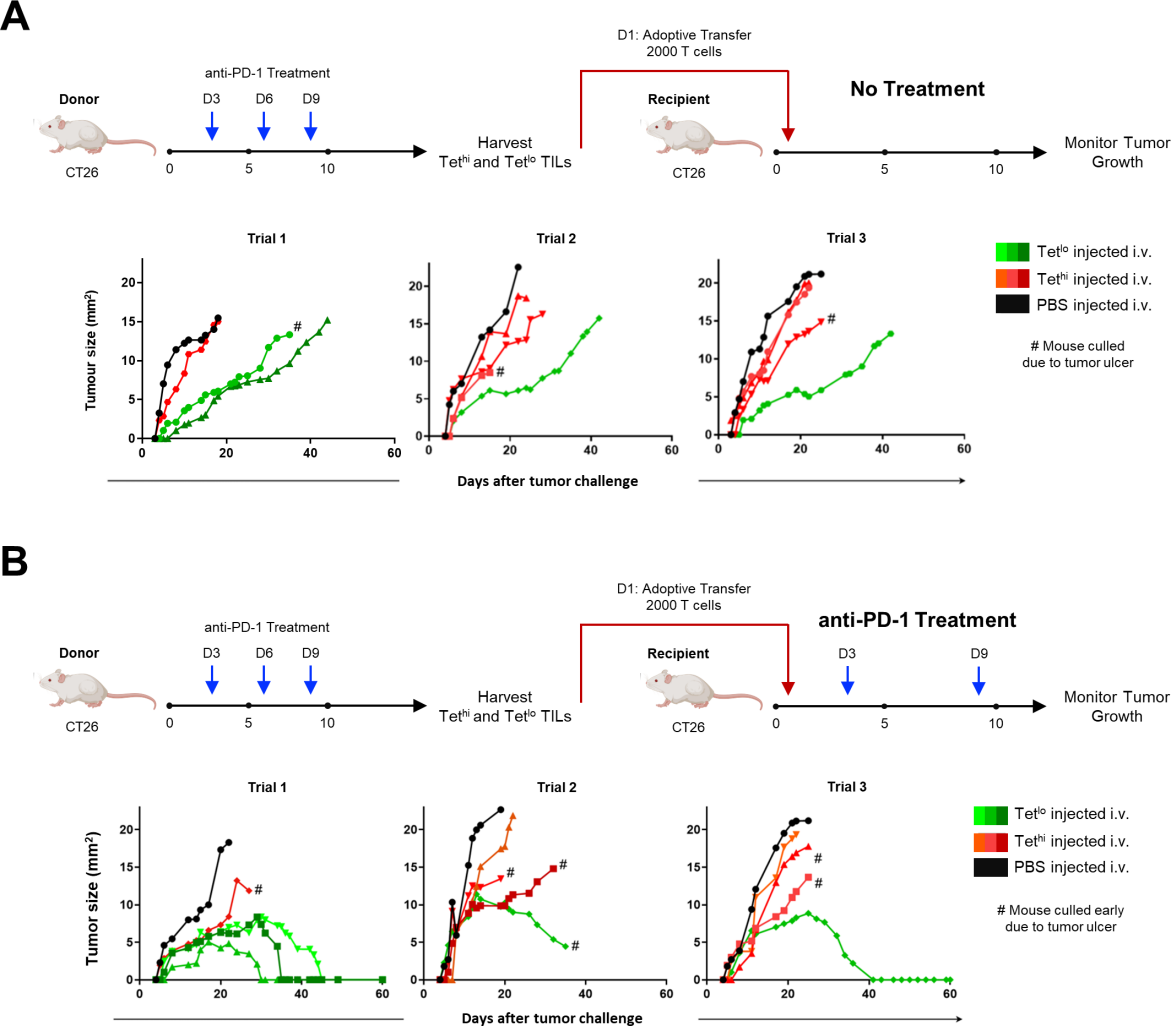
Low avidity GSW11-specific CD8^+^ T cells demonstrated better tumor control in adoptive transfer and curative response was detected in combination with anti-PD-1 therapy. (A) Treatment strategy for adoptive transfer of 2000 Tet^hi^ or Tet^lo^ GSW11-specific isolated from anti-PD-1 treated donor mice into recipient tumor mice. Tumor growth curves of mice adoptively transferred with tumor-specific T cell subsets or phosphate buffered saline (PBS) as control. (B) Treatment strategy for adoptive transfer of 2000 Tet^hi^ or Tet^lo^ GSW11-specific isolated from anti-PD-1 treated donor mice into recipient mice, with additional anti-PD-1 treatment on days 3 and 9. Tumor growth curves of mice adoptively transferred with tumor-specific T cell subsets or PBS as control and treated with anti-PD-1. The tumor growth curves of mice injected with Tet^lo^ GSW11-specific CD8^+^ T cells are color coded as shades of green, mice injected with Tet^hi^ are in shades of red to orange, while mice injected with PBS are color-coded as black. TIL, tumor-infiltrating lymphocyte.

We determined the in vivo cytotoxic function of Tet^hi^ and Tet^lo^ GSW11-specific CD8^+^ TILs isolated from t-DLNs in controlling tumor growth. TILs were adoptively transferred into recipient tumor mice 1 day following tumor inoculation. Figure 6A shows that Tet^lo^ GSW11-specific T cells were more effective at controlling tumor growth over a period of 40 days than their Tet^hi^ counterparts. By combining adoptive T cell transfer with two doses of anti-PD-1 administered on days 3 and 9 (2 and 8 days after transfer), we found that little improvement in therapeutic efficacy was achieved in mice transferred with Tet^hi^, whereas curative responses were achieved for Tet^lo^ as shown in figure 6B.

## Discussion

T cell avidity plays a crucial role in antigen presentation and influences the quality of TCR signaling and T cell metabolic fitness. This phenomenon is governed by the overall strength of multiple TCR/pMHC engagements and the effects of costimulatory and coinhibitory interactions.^42^ T cells with high functional avidity have been known to respond to very low antigen doses, while T cells with lower functional avidity require higher antigen doses to mount a similar level of immune response. In cancer, antigen persistence and the inflammatory microenvironment can induce a tolerant state in T cells, leading to hyporesponsiveness, loss of effector function and defective TCR signaling in response to chronic antigen stimulation.^43^ High avidity T cells are known to impose greater selective pressure for antigen loss leading to the outgrowth of tumor cell clones with reduced antigenicity that are more likely to selectively activate high avidity T cells and indirectly lead to exhaustion through chronic stimulation.^44^ Although the superiority of high avidity T cells in cancer and infections is often asserted,^45 46^ other studies have suggested the importance of low avidity T cells for controlling chronic viral infections and established tumors in the presence of persistent antigen engagements^47 48^ and response to self-antigens.^48 49^ A recent study also demonstrated the preferential expansion of T cells with low affinity for the tumor-specific antigen (ovalbumin) in PD-1 deficient mice inoculated with EG7 mouse thymoma.^50^ Therefore, a deeper understanding of the mechanisms and pathways leading to T cell exhaustion in PD-1 immunotherapy, including the role of CD8^+^ specificities and avidity, and a clear demonstration that the strength of individual TCR signals is the key determinant of anti-PD-1 sensitivity is needed.

In this study, we showed that the therapeutic effects of anti-PD-1 in the CT26 model correlate with the preferential expansion of low avidity tumor-specific CD8^+^ T cells (Tet^lo^) with a precursor exhausted phenotype and high cytotoxic function. This is consistent with recent studies which showed that ICIs acts to promote differentiation of a subset of T cell with stem-like properties, expressing the transcription factor Tcf-1 and lacking markers of terminal exhaustion such as Tim-3,^12 51–53^ and for the first-time links anti-PD-1 responsive phenotype to T cell avidity. By investigating the functional phenotype of naturally arising T cells to the identical pMHC, we were able to isolate the impact of T cell avidity on therapeutic rescue by anti-PD-1, and investigate how this treatment effect is ameliorated when both high and low avidity T cells recognizing the same pMHC coexist in the same tumor. In addition, we have previously demonstrated the protective role of low avidity GSW11-specific CD8^+^ T cells in a second immunotherapeutic setting—notably Treg-depletion.^15^ In this study, we showed that treatment with anti-CD25 induced the preferential expansion of low avidity CD8^+^ oligoclones which has a ‘less exhausted’ phenotype and correlated with protection, although this phenomenon cannot be generalized to other immunotherapeutics for example, anti-CTLA-4, anti-TIM-3 and anti-LAG3 as different interventions may have distinct mechanism of action.

Reduced functional avidity promotes central and effector memory CD4^+^ T cell responses to tumor-associated antigens.^54^ The avidity of TCRs has been found to be negatively correlated with tumor-antigen abundance in melanoma patients and TCRs with low avidity and strong tumor recognition have been found in tumors with high expression of tumor-associated antigens.^55^ In contrast, high avidity CD8^+^ T cells are known to undergo exhaustion and antigen-dependent apoptosis in the presence of persistent antigens during chronic viral infections.^56^ These phenomena were found in our experiments where the genes and pathways associated with antigen presentation, TCR signaling, T cell cytotoxic function, and oxidative phosphorylation were significantly upregulated or enriched in Tet^lo^ found in regressing tumors compared with Tet^hi^ in progressing tumors; whereas genes related to DNA damage, apoptosis and autophagy were downregulated. Furthermore, the low avidity population exhibited higher expression of TCF-1 and T-bet, and lower expression of the exhaustion markers CD39, PD-1 and Eomes similar to precursor exhausted or progenitor T cells known to display self-renewing capacity and maintain long-term persistent T cell responses.^57 58^ Although Tet^lo^ from both the regressing and progressing tumors exhibited similar precursor exhausted features and transcriptomics profiles and were both enriched for hallmarks pathways related to allograft rejection, DNA repair, and oxidative phosphorylation, tumor control was not achieved in the progressing tumor and in the presence of Tet^hi^. It is possible that oxygen tension and metabolic immunosuppression within the progressing tumor microenvironment could have suppressed Tet^lo^ function as genes related to hypoxia and metabolic reprogramming such as Cnyp2 and Bckdhb were differentially expressed in Tet^lo^ found in progressing tumors compared with those in the regressing tumors, and gene sets associated with metabolism adaptation, that is, non-coding RNA metabolic process pathway as well as regulation of ion transport and catabolic processes were significantly enriched. On the other hand, it is possible that exhausted Tet^hi^ in progressing tumors may have contributed to the suppressive effects on the Tet^lo^ population. It has been known that PANX1 which is a caspase-mediated channel that conducts ToPro-3 iodide into cells, can also release ATP from cells.^59^ One intriguing consequence of inducing repairable cell damage with sublytic granules, as opposed to commitment to full apoptosis, as suggested by in vitro killing assays of Tet^hi^, is that the former may lead to the release of ATP and potassium ions from target cells without target killing.^60 61^ The ectonucleoside triphosphate diphosphohydrolase-1 or CD39 is expressed on tumor-reactive T cell populations in cancers.^62^ Tet^hi^ expresses high levels of CD39 which, together with the ecto-5’ nucleotidase CD73, could potentially metabolize extracellular ATP to generate high local concentrations of immunosuppressive adenosine that acts on both Tet^hi^ and Tet^lo^ recognizing the same epitope. Furthermore, a recent study showed that terminally exhausted CD8^+^ T cells in hypoxic tumors are capable of suppressing tumor-specific T cell populations in vivo and are dependent on the high expression of CD39 for generating immunosuppressive adenosine.^63^

One of the limitations of our study is the use of only one mouse tumor model. We focused on CT26 as the model system for this study as it is the most extensively investigated syngeneic mouse tumor models in preclinical studies and has been used to validate most immune checkpoint blockade immunotherapeutics currently in the clinic or in clinical trials, with well over 500 studies in literature. Previously, we have mapped the tumor antigen landscape of the CT26 model where the novel tumor epitope GSW11 has been shown to be abundantly expressed in CT26 tumors and contributed to the immunodominant response among tumor-infiltrating CD8^+^ T cells.^21^ However, despite the presence of T cell infiltration in tumors, CT26 is not immunogenic and is only moderately responsive to anti-PD-1. One of the reasons may be cancer immunoediting,^64^ TCR avidity and chronic stimulation,^55^ thus we moved on to investigate the underlying mechanisms within the setting of the CT26 model. It is also important to note that other immune cells in the CT26 tumor microenvironment such as CD4^+^ T helper cells may have contributed to the expansion of low avidity tumor-specific CD8^+^ T cells in the presence of anti-PD-1. While an in-depth analysis of CD4^+^ T helper cells lies outside the scope of this study, Jin *et al* have previously shown that CD4^+^ T cell depletion prior to CT26 tumor installation has very little effect on tumor growth, and does not affect anti-PD-1 efficacy, in contrast to CD8^+^ T cell depletion.^16^ This suggests that the role of T helper cells in tumor control both naturally and in the context of anti-PD-1 may be difficult to define.

pMHC affinity is important for the identification of neoepitopes in cancer vaccines. Our results indicate that targeting subdominant T cell responses with lower avidity against pMHC affinity neoepitopes may be a viable therapeutic strategy: including vaccination approaches that enrich TCF-1^+^ T cells. Because TCF-1^+^ cells repopulate the cytotoxic T cell pool and respond to anti-PD-1, engaging subdominant T cell responses may result in more durable tumor control and a better response to ICIs. Future therapeutic approaches may consider interventions to expand the low avidity T cell population via vaccination or adoptive T cell transfer and in combination with anti-PD-1 or drugs targeting immunometabolism to boost treatment efficacy.

10.1136/jitc-2023-007114.supp4 Supplementary data

## Data Availability

Data are available on reasonable request. RNA sequencing data have been deposited in the Gene Expression Omnibus (GEO) under accession number GSE221590. All algorithms used for RNAseq analysis were publicly available R packages. Additional information required is available from the lead contact on request.
