# Correction: Reactivation of low avidity tumor-specific CD8+ T cells associates with immunotherapeutic efficacy of anti-PD-1

**DOI:** 10.1136/jitc-2023-007114corr1

**Published:** 2023-08-28

Sugiyarto G, Lau D, Hill SL, et al. Reactivation of low avidity tumor-specific CD8+ T cells associates with immunotherapeutic efficacy of anti-PD-1. Journal for ImmunoTherapy of Cancer 2023;11:e007114. doi: 10.1136/jitc-2023-007114

The author Tim Elliott has been made a corresponding author and Eileen E Parkes was incorrectly listed as Eileen Parkes. The third affiliation has been updated to Department of Oncology, University of Oxford, Oxford, UK.

