# Supplementary material for: Reactivation of low avidity tumor-specific CD8+ T cells associates with immunotherapeutic efficacy of anti-PD-1
Source: J Immunother Cancer. 2023 Aug 16;11(8):e007114. doi: 10.1136/jitc-2023-007114 (PMC10432680; doi:10.1136/jitc-2023-007114)
Supplement: Supplementary data [file jitc-2023-007114supp005.pdf]

Supplemental Materials

Supplemental Figure 1.

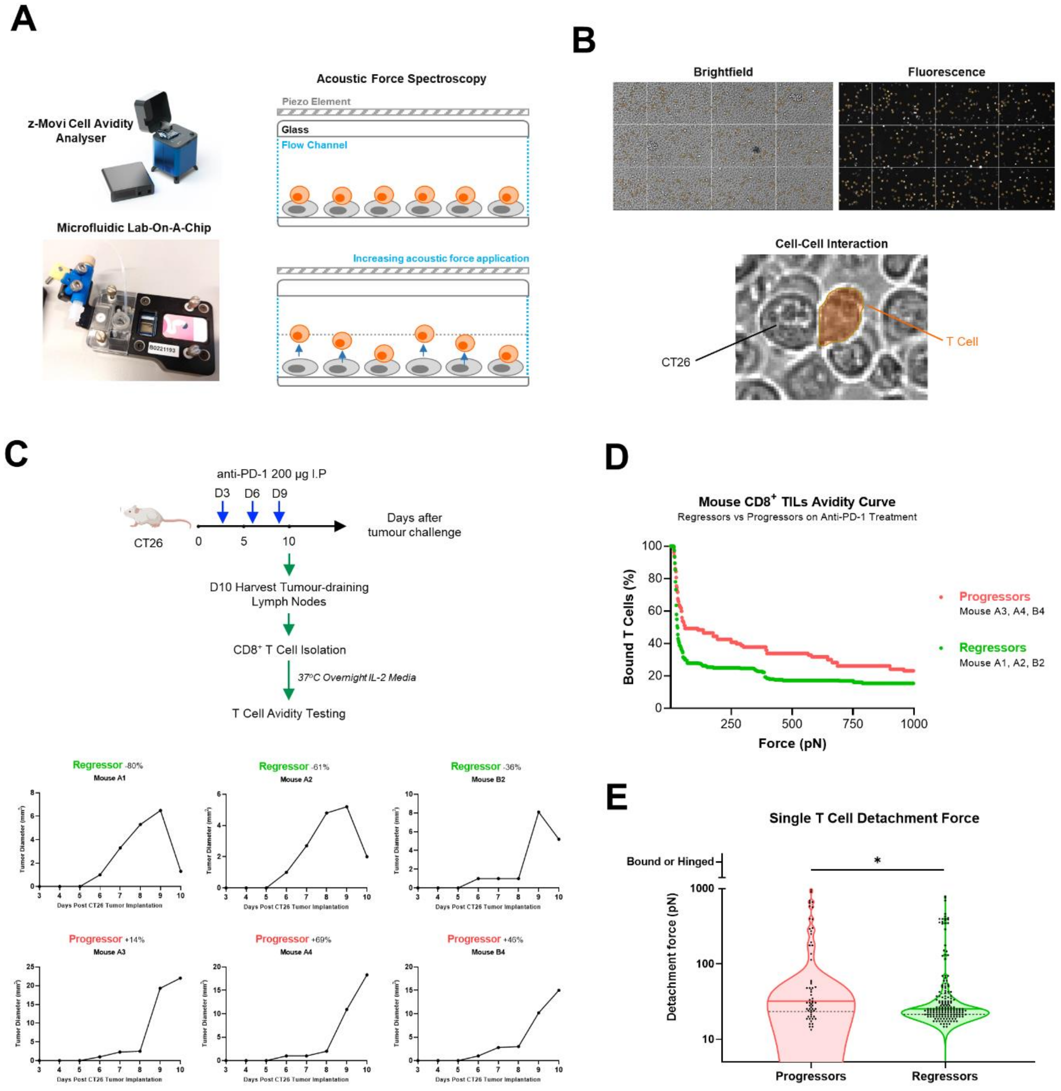

Biophysical measurement of T cell avidity of $CD8^+$ TILs isolated from t-dLNs of regressors and progressors following anti-PD-1 treatment. (A) Direct biophysical measurement of the amount of force (pN) required to detach the effector T cells from CT26 cells was performed using acoustic force spectroscopy with the LUMICKS Z-Movi cell avidity analyzer. (B) Image-based tracking of single fluorescently labelled $CD8^+$ T cells and their interactions with CT26 cells. (C) $CD8^+$ TILs were isolated from the t-dLNs of regressors and progressors treated with anti-PD-1, with the tumor growth and response curves from individual mice shown. (D) T cell avidity curve expressed in terms of percentage bound T cells and (E) single T cell detachment force comparing between the TCR avidity of $CD8^+$ TILs isolated from progressors versus regressors.

Supplemental Figure 2.

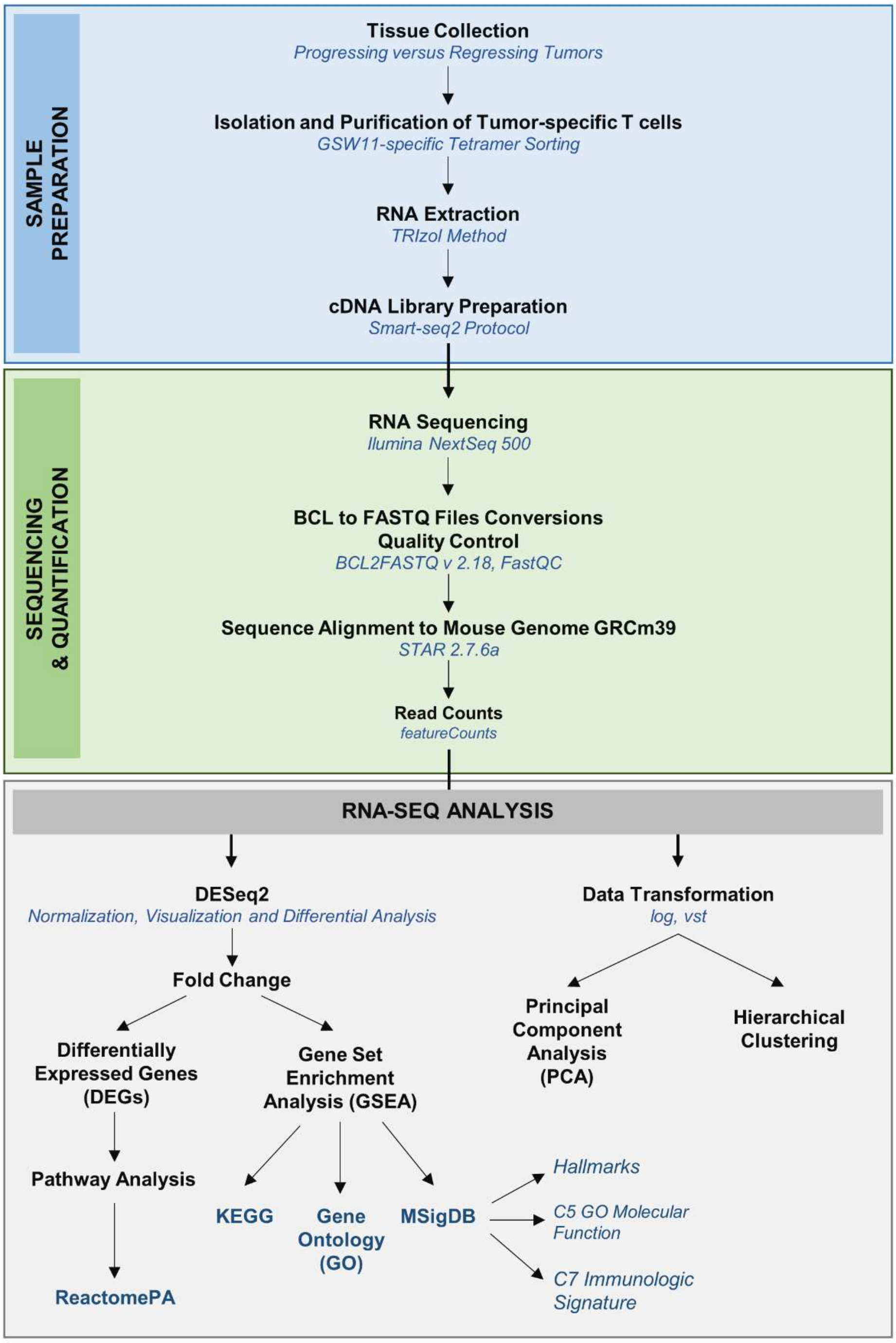

Pipeline for preparation of RNA samples, bulk RNA-seq and analysis.

Supplemental Figure 3.

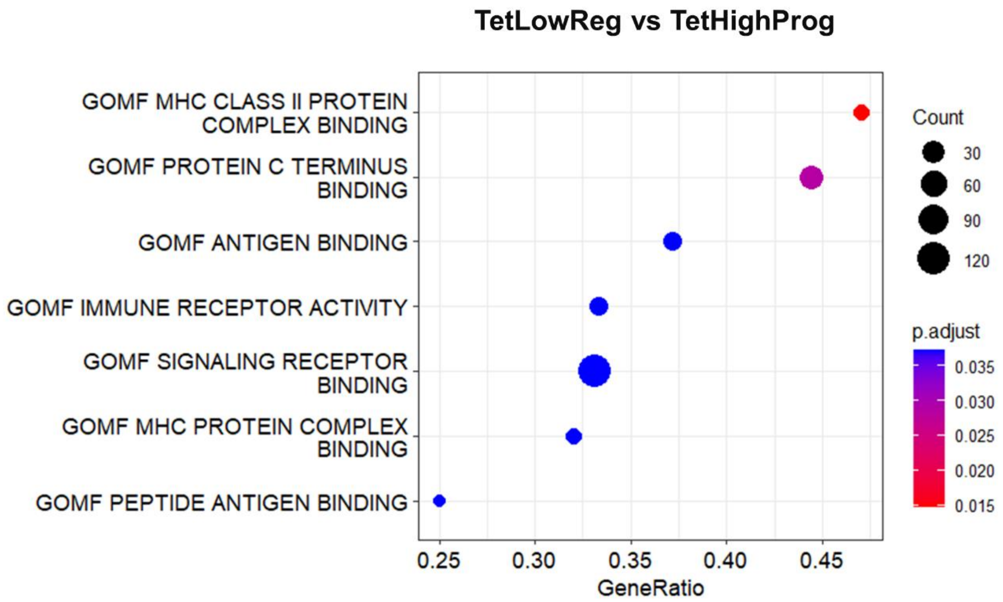

Gene Set Enrichment Analysis (GSEA) using MSigDb C5 Gene Ontology Molecular Function (GOMF). Dotplot showing significantly enriched cellular processes in TetLowReg *vs.* TetHighProg. Size of the count represents the gene counts of the significantly enriched GOMF biological processes and significance was determined by the adjusted *p*-values.

Supplemental Figure 4.

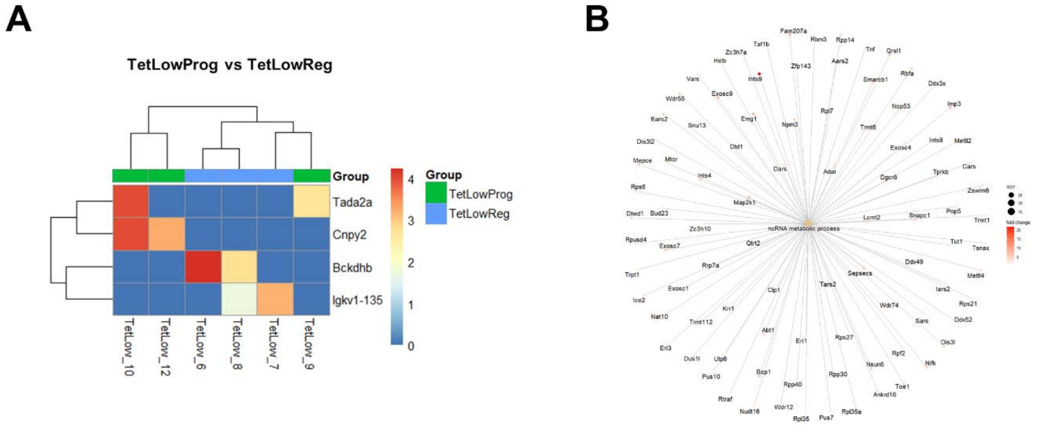

Differential gene expression and gene ontology enrichment analysis of TetLowProg *vs.* TetLowReg. (**A**) Heatmap of DESeq2 analysis of differentially expressed genes in TetLowProg *vs.* TetLowReg. (**B)** GO term analysis showed significant enrichment in metabolic processes involving non-coding RNA transcripts.

Supplemental Figure 5.

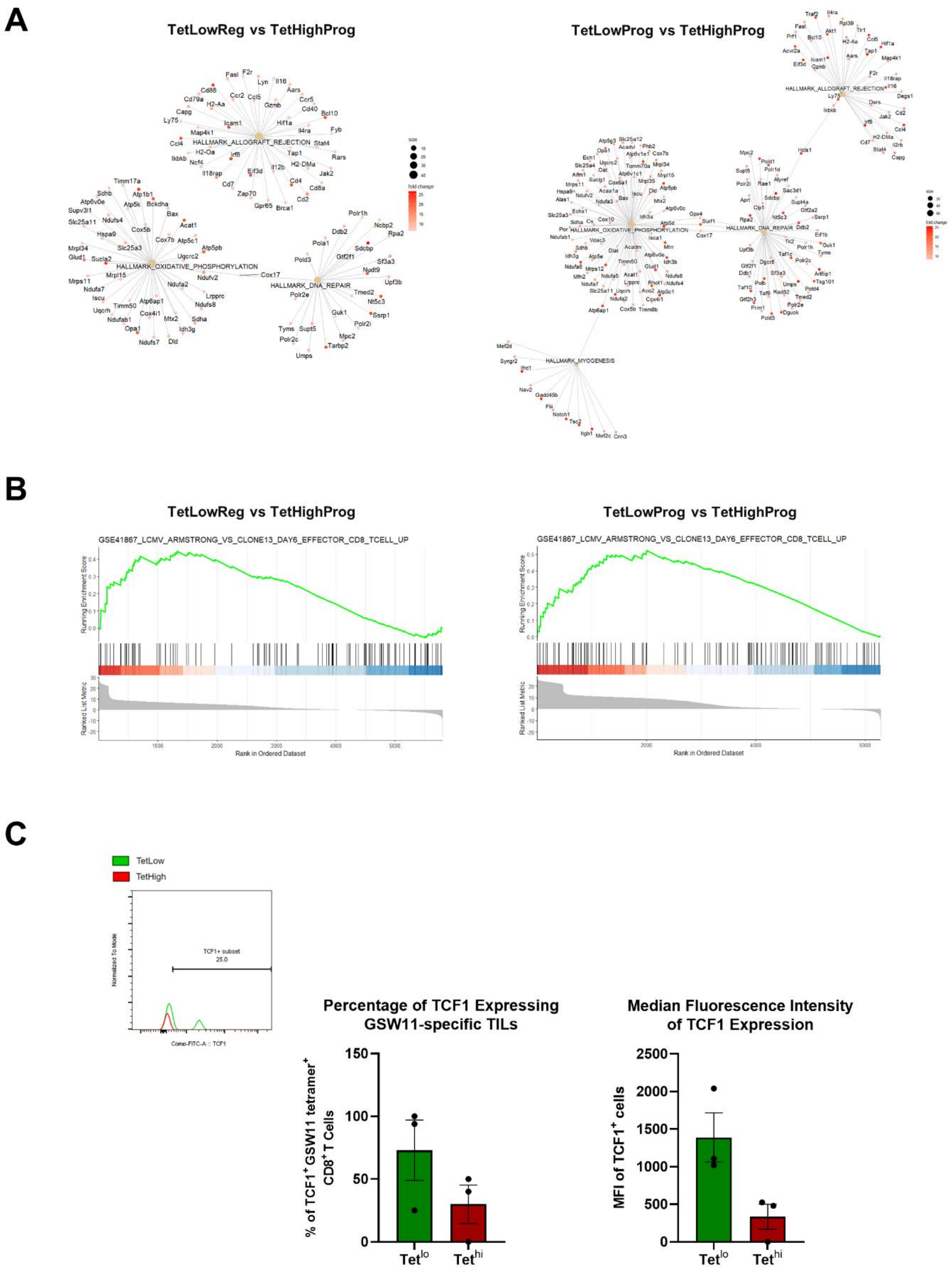

Comparison between the transcriptomic and functional profiles of Tetlo GSW11-specific CD8$^+$ T cells found in regressing and progressing tumors. (**A**) GSEA analysis using MSigDb Hallmark gene sets showing the shared pathways between TetLowReg and TetLowProg. The scalebars of the node size reflects the number of significantly enriched genes in the node and fold change indicates the level of gene expression. (**B**) Running enrichment score and preranker list plots of GSEA analysis using the MSigDb C7 Immunologic Signature (GSE41867) for TetLowReg and TetLowProg compared to TetHighProg. (**C**) Flow cytometric analysis of Tcf-1 expression on GSW11-specific CD8+ TILs in the form of a histogram plot, and percentage and median fluorescence intensity (MFI) bar plots.

Supplemental Figure 6.

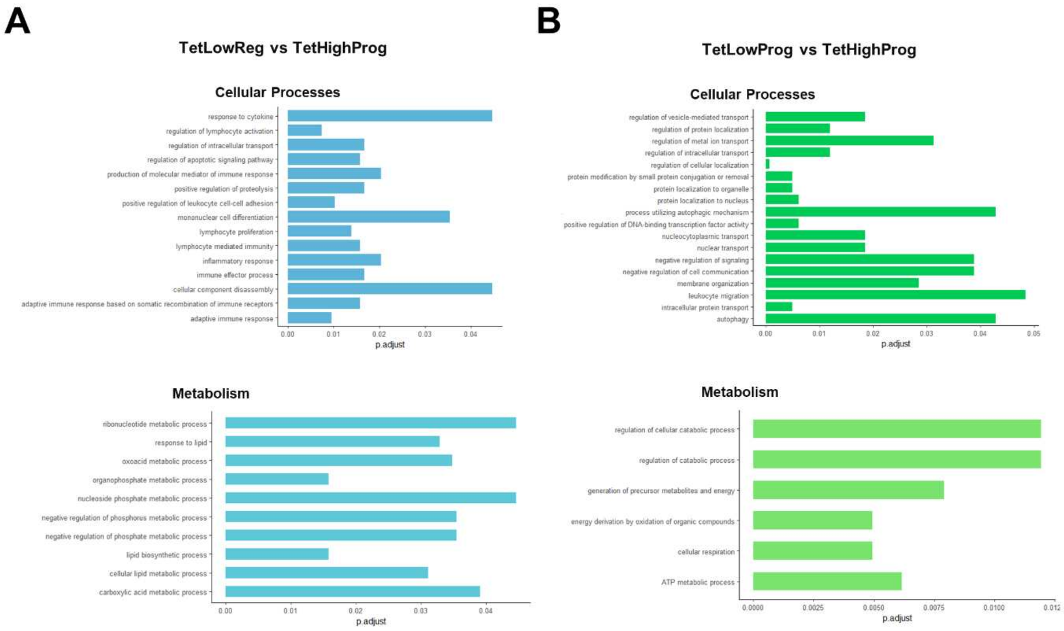

Gene Ontology (GO) term analysis of significantly enriched cellular and metabolic processes in (**A**) TetLowReg and (**B**) TetLowProg compared to TetHighProg.

Supplemental Table 1.

Tumor growth monitoring of individual CT26 tumor models on anti-PD-1 therapy. The tumor growth was measured based on tumor size or diameter (mm^2). Response was determined based on the REET score.

Supplemental Table 2.

List of differentially expressed genes in TetLowReg *vs.* TetHighProg. 1054 genes were upregulated, and 17 genes were downregulated in $Tet^{lo}CD8^{+}CD44^{+}$ GSW11-specific T cells isolated from the regressing tumors compared to their high avidity counterparts from the progressing tumors.

Supplemental Table 3.

List of enriched REACTOME pathways in TetLowReg *vs*. TetHighProg. Immune-related biological pathways are highlighted as green.

Supplemental Methods

Response Evaluation in Early Tumors

Tumor progression or regression of anti-PD-1 treatment was determined by a Tumor Control Index criterion [19] where a Response Evaluation in Early Tumors (REET) score of 0 indicates that the tumor is progressing; a score of 1 represents a tumor that has regressed by less than 10% per day since the last measurement timepoint; a score of 2 indicates that the tumor has regressed by more than 10% per day since the last measurement timepoint, and a score of 3 indicates a tumor that has regressed by more than 10% per day for two consecutive days. The study endpoint for each experimental mouse was reached when a REET score of 2 or more was met.

Bulk RNA-seq

Tet^{hi} and Tet^{lo} GSW11-specific $CD8^+$ T cells were sorted by FACS from regressing and progressing tumors and harvested into TRIzol reagent for bulk RNA-seq. Full-length libraries were prepared using the Smart-seq2 protocol as described by Picelli et al. [20]. Briefly, RNA extraction and purification were performed using a Zymo Quick RNA Miniprep Kit (Zymo, USA) prior to cDNA synthesis. The purified RNA was quantified using a Qubit 4 Fluorometer (Invitrogen) and the quality was determined using an Agilent 2100 Bioanalyzer with the Agilent RNA 6000 Nano Kit (Agilent Technologies). RNA was reverse transcribed using SuperScript II (Invitrogen) and cDNA pre-amplification was performed using KAPA HiFi HotStart ReadyMix (Roche Molecular Systems) for 22 cycles of amplification. cDNA libraries were purified using Agencourt AMPure XP beads (Beckman Coulter) according to the manufacturer's

instructions. The cDNA product was analyzed using an Agilent 2100 Bioanalyzer with an Agilent High Sensitivity DNA kit (Agilent Technologies). Tagmentation and library preparation were performed using an Illumina Nextera XT DNA Library Preparation Kit (Illumina) with 150 pg of purified cDNA from each sample. The tagmented and indexed PCR products were further purified using Agencourt AMPure XP beads, and the DNA concentration, average fragment size and molarity were confirmed using a Bioanalyzer and Qubit assessment. Paired-end sequencing of the pooled libraries was performed on the NextSeq 500 platform using a high output kit v2.5 for 75 cycles. A total of 400 million single end reads (Illumina) libraries from nine samples were multiplexed to give in excess of the targeted 30 million single end reads per library.

The Binary Base Call (BCL) files from the sequencing run were converted into FASTQ files using the BCL2FASTQ software package version 2.18 and transferred to the University of Southampton IRIDIS High Performance Computing Cluster. Following demultiplexing, the RNA reads were checked for sequence quality using FastQC (https://www.bioinformatics.babraham.ac.uk/projects/fastqc/). The paired-end reads were aligned to the mouse reference genome GRCm39 using STAR 2.7.6a and the mapped reads were assigned genomic features using *featureCounts*. Differential gene expression analysis was performed in the R software version 4.2.1 environment using the DESeq2 package 1.36.0 which involved the Wald test and Benjamini-Hochberg correction to obtain the log fold change in gene expression levels between conditions, normalized gene counts, and examination of sample-level variation using principal component analysis. Significance was defined as $P < 0.05$, $\log_2$ fold change > 2, and differentially expressed genes were visualized in plots

using *pheatmap* for hierarchical clustering and *EnhancedVolcano*. Gene set enrichment analysis (GSEA) was performed using the Kyoto Encyclopaedia of Genes and Genomes (KEGG), Gene Ontology (GO) and Molecular Signatures Database (MSigDB) databases in clusterProfile 4.0. A more detailed pathway analysis of the transcriptome based on the REACTOME database was performed using ReactomePA. The *P* values for pathway enrichment analyses were calculated using a hypergeometric test and significantly enriched pathways were identified using a threshold false discovery rate (FDR) of 0.05. Data visualization was conducted using *enrichplot*.

Biophysical measurement of T cell avidity

$CD8^+$ T cells were isolated from individual t-dLNs of regressors and progressors (anti-PD-1 treated mice) using magnetic-activated cell sorting by negative selection (Miltenyi Biotec). T cells were allowed to rest overnight in 10% FBS RPMI-1640 supplemented with 20 U/mL of recombinant IL-2 (Peprotech Inc.) and maintained at 37°C, 5% CO_2 in a humified atmosphere. For acoustic force spectroscopy using the Z-Movi cell avidity analyzer (LUMICKS, The Netherlands), microfluidic chips were first functionalized with 1M NaOH followed by coating with poly-L-lysine (Sigma-Aldrich) and rehydration with prewarmed media. Target cell monolayer seeding was then performed with CT26 cells (80% confluency) at a seeding density of 100 million cells/mL and frequent checking under the microscope to ensure no bubble formation and appropriate seeding and cellular density. Cells were incubated for 2 h at 37°C, with fresh change of media in between. $CD8^+$ T cells isolated from the tdLN of individual mouse were pulsed with 10 μM GSW11 peptide in the presence of IL-2 for 2 h at 37°C before staining with CellTrace Far Red Proliferation Kit (ThermoFisher

Scientific) according to manufacturer's instructions. The labelled cells were then seeded into the microfluid chip followed by incubation for 10 min with the CT26 monolayer in the presence of GSW11 peptide, prior to acoustic force application and image-based fluorescent T cell tracking. CD8^{+} T cells from all mice were evaluated on the same microfluidic chips and the order was randomized between chips on repeated runs. Automated detection of T cell detachment force was performed using the Oceon software (LUMICKS).
