# Supplementary material for: Reactivation of low avidity tumor-specific CD8+ T cells associates with immunotherapeutic efficacy of anti-PD-1
Source: J Immunother Cancer. 2023 Aug 16;11(8):e007114. doi: 10.1136/jitc-2023-007114 (PMC10432680; doi:10.1136/jitc-2023-007114)
Supplement: Supplementary data [file jitc-2023-007114supp001.pdf]

Response Evaluation in Early Treatment (REET) Score

Based on Tumor Control Index criterion[18]

CT26 mouse colorectal carcinoma, anti-PD-1 treatments on day 3, 6 and 9

REET Score

0 = Growing
1 = Shrinking but less that 10% per day since last measurement point
2 = Shrinking more than 10% per day since last measurement point
3 = Shrinking more than 10% per day for 2 consecutive days

*Each row represents the scoring for 1 mouse.

Days post tumor implantation	Trial 1						Trial 2					
0	0	0	0	0	0	0	0	0	0	0	0	0
2	0	0	0	0	0	0	0	0	0	0	0	0
3	0	0	0	0	0	0	0	0	0	0	0	0
4												
5												
6	0	0	0	0	0	0	0	0	0	0	0	0
7												
8	0	0	0	0	1							
9	1	0	0	0	0	0	0	0	0	0	0	0
10	2	0	0	0	0	0	0	0	0	1	1	0
11						0	0	0	2	0	0	0
12												

Days post tumor implantation	Trial 3					Trial 4				
0	0	0	0	0	0	0	0	0	0	0
2						0	0	0	0	0
3						0	0	0	0	0
4	0	0	0	0	0					
5										
6						0	0	0	0	0
7	0	0	0	0	0					
8	0	0	0	0	0	0	0	0	0	1
9	0	0	0	0	2	1	0	0	0	0
10	0	0	0	1	0	2	0	0	0	0
11	0	0	0	1	2					
12										

Days post tumor implantation	Trial 5							Trial 6					
0	0	0	0	0	0	0	0	0	0	0	0	0	0
2	0	0	0	0	0	0	0	0	0	0	0	0	0
3													
4	0	0	0	0	0	0	0	0	0	0	0	0	0
5													
6								0	0	0	0	0	0
7	0	0	0	0	0	0	0						
8	0	0	0	0	0	0	0	0	0	0	0	0	0
9	0	0	0	0	0	1	0	0	2	2	2	0	0
10	0	0	1	0	0	1	0	0	3	0	0	0	0
11	0	0	0	0	2	2	0						
12													

Days post tumor implantation	Trial 7					Trial 8				
0	0	0	0	0	0	0	0	0	0	0
2	0	0	0	0	0	0	0	0	0	0
3	0	0	0	0	0	0	0	0	0	0
4										
5										
6	0	0	0	0	0	0	0	0	0	0
7	0	0	0	0	0	0	0	0	0	0
8										
9	0	0	0	0	0	0	2	0	1	0
10	1	1	0	0	0					
11										
12	0	2	0	0	0					

Days post tumor implantation	Trial 9					Trial 10					
0	0	0	0	0	0	0	0	0	0	0	0
2	0	0	0	0	0						
3	0	0	0	0	0	0	0	0	0	0	0
4						0	0	0	0	0	0
5						0	0	0	0	0	0
6	0	0	0	0	0						
7	0	0	0	0	0	0	1	0	0	0	0
8											
9	0	0	0	0	2	0	0	0	0	0	0
10	0	1	1	0	3	0	0	0	0	2	2
11											
12											

Days post tumor implantation	Trial 11							Trial 12					
0	0	0	0	0	0	0	0	0	0	0	0	0	0
2	0	0	0	0	0	0	0	0	0	0	0	0	0
3													
4	0	0	0	0	0	0	0	0	0	0	0	0	0
5													
6								0	0	0	0	0	0
7	0	0	0	0	0	0	0						
8													
9	0	0	0	0	0	0	0	0	0	0	0	0	0
10	2	1	0	2	0	0	0	2	0	1	0	0	0
11	3	0	0	3	0	0	0	3	0	0	0	0	0
12													
