# Supplementary material for: Reactivation of low avidity tumor-specific CD8+ T cells associates with immunotherapeutic efficacy of anti-PD-1
Source: J Immunother Cancer. 2023 Aug 16;11(8):e007114. doi: 10.1136/jitc-2023-007114 (PMC10432680; doi:10.1136/jitc-2023-007114)
Supplement: Supplementary data [file jitc-2023-007114supp002.pdf]

Differentially Expressed Genes

TetLowReg vs TetHighProg

Upregulated Transcripts = 1054

	Ensembl	Symbol	baseMean	log2FoldChange	lfcSE	pvalue	padj
1	ENSMUSG00000060586	H2-Eb1	8239.258	28.01781781	3.818015	1.60E-14	6.28E-11
2	ENSMUSG00000073421	H2-Ab1	3236.9073	26.7801298	3.846313	1.99E-13	2.32E-10
3	ENSMUSG00000003518	Dusp3	2901.3361	26.64055804	3.850431	2.62E-13	2.32E-10
4	ENSMUSG00000028249	Sdcbp	2830.8791	26.60491133	3.851517	2.81E-13	2.32E-10
5	ENSMUSG00000031902	Nfatc3	2762.9157	26.57777681	3.852353	2.96E-13	2.32E-10
6	ENSMUSG00000035086	Becn1	2125.4333	26.2148594	3.864344	5.98E-13	3.83E-10
7	ENSMUSG00000037922	Bank1	2012.206	26.14503767	3.86683	6.84E-13	3.83E-10
8	ENSMUSG00000032359	Ctsh	1883.6203	26.05350273	3.870179	8.14E-13	3.99E-10
9	ENSMUSG00000051223	Bzw1	1484.5715	25.71935395	3.883305	1.53E-12	6.65E-10
10	ENSMUSG00000029725	Ppp1r35	1295.8784	25.53295799	3.891261	2.16E-12	8.46E-10
11	ENSMUSG00000052459	Atp6v1a	1036.2451	25.22509116	3.905432	3.80E-12	1.24E-09
12	ENSMUSG00000028470	Hint2	972.00766	25.13140981	3.910005	4.50E-12	1.36E-09
13	ENSMUSG00000041515	Irf8	868.80528	24.98150272	3.917578	5.89E-12	1.65E-09
14	ENSMUSG00000019876	Pkib	729.31674	24.73362733	3.9308	9.14E-12	2.24E-09
15	ENSMUSG00000022901	Cd86	725.73396	24.60611157	3.937936	1.14E-11	2.63E-09
16	ENSMUSG00000040462	Os9	577.94679	24.40299913	3.949797	1.63E-11	3.54E-09
17	ENSMUSG00000026576	Atp1b1	539.16515	24.29895476	3.956096	1.94E-11	4.01E-09
18	ENSMUSG00000029249	Rest	554.90702	24.16259241	3.96457	2.45E-11	4.41E-09
19	ENSMUSG00000025862	Stag2	757.9967	24.15614414	3.964953	2.48E-11	4.41E-09
20	ENSMUSG00000022110	Sucla2	558.498	24.1438748	3.965753	2.53E-11	4.41E-09
21	ENSMUSG00000067149	Jchain	742.60936	24.12978251	3.966625	2.59E-11	4.41E-09
22	ENSMUSG00000028577	Plaa	456.93416	24.03681595	3.972637	3.03E-11	4.95E-09
23	ENSMUSG00000005583	Mef2c	589.48212	23.67456645	3.996982	5.53E-11	8.35E-09
24	ENSMUSG00000021023	Prorp	335.5627	23.62549153	3.990916	5.55E-11	8.35E-09
25	ENSMUSG00000064065	Ipcef1	293.37338	23.42189702	4.006167	7.77E-11	1.13E-08
26	ENSMUSG00000026068	Il18rap	13096.696	15.96382209	3.029387	2.28E-10	3.18E-08
27	ENSMUSG00000025279	Dnase1l3	19897.583	15.57532071	3.423631	1.23E-08	1.55E-06
28	ENSMUSG00000027863	Cd2	7587.5533	15.46491448	2.636075	6.88E-12	1.80E-09
29	ENSMUSG00000025138	Sirt7	7787.6526	14.60354297	2.420342	3.33E-12	1.19E-09
30	ENSMUSG00000032026	Rexo2	4128.5554	14.07018023	2.925016	5.51E-09	7.19E-07
31	ENSMUSG00000030744	Rps3	4939.7898	13.67700567	2.582076	4.93E-10	6.65E-08
32	ENSMUSG00000024725	Ostf1	2710.1252	13.23587347	2.991841	6.28E-08	7.69E-06
33	ENSMUSG00000037236	Matr3	3279.535	12.55538884	2.877359	1.32E-07	1.57E-05
34	ENSMUSG00000027808	Serp1	2440.0874	12.55342447	3.309918	1.63E-06	0.000172
35	ENSMUSG00000036594	H2-Aa	16428.338	12.47181921	4.077956	2.78E-05	0.002131
36	ENSMUSG00000032557	Uba5	2814.1519	12.23471196	2.935746	4.06E-07	4.67E-05
37	ENSMUSG00000046679	C87436	1491.6064	12.04014082	3.088461	1.48E-06	0.000161
38	ENSMUSG00000018322	Tomm34	1872.137	11.98971471	3.358511	5.68E-06	0.000483
39	ENSMUSG00000019947	Arid5b	1405.1614	11.94414368	3.083699	1.75E-06	0.000181
40	ENSMUSG00000028902	Sf3a3	1617.4482	11.93916781	3.235883	3.68E-06	0.000352
41	ENSMUSG00000041720	Pi4ka	1502.7131	11.92307573	3.168084	2.78E-06	0.000279

42	ENSMUSG00000037822	Smim14	1550.5203	11.83072279	3.255668	4.92E-06	0.000438
43	ENSMUSG00000017677	Wsb1	1320.0513	11.73333842	3.146243	3.62E-06	0.000352
44	ENSMUSG00000061288	Taok3	2046.2032	11.69377281	2.916954	1.19E-06	0.000133
45	ENSMUSG00000025066	Sfr1	1370.7081	11.68104229	3.216313	5.49E-06	0.000478
46	ENSMUSG00000033713	Foxn3	1125.5677	11.53050021	3.103049	4.40E-06	0.000401
47	ENSMUSG00000028581	Laptm5	2091.7241	11.49333537	3.083886	4.32E-06	0.000401
48	ENSMUSG00000026399	Cd55	2920.3694	11.31285259	3.646055	4.59E-05	0.003102
49	ENSMUSG00000024259	Slc25a46	968.67821	11.26311251	3.110353	7.63E-06	0.000635
50	ENSMUSG00000091095	NA	1021.6251	11.11024501	3.252025	1.79E-05	0.001433
51	ENSMUSG00000030330	Ing4	839.26941	10.99156773	3.128424	1.37E-05	0.00112
52	ENSMUSG00000018474	Chd3	823.83816	10.78864884	3.227685	2.88E-05	0.002131
53	ENSMUSG00000024238	Zeb1	1759.6979	10.69585302	3.496434	7.77E-05	0.004755
54	ENSMUSG00000056737	Capg	1365.0151	10.68685794	3.120779	2.33E-05	0.001822
55	ENSMUSG00000076614	NA	911.7756	10.62807009	3.410701	6.78E-05	0.004352
56	ENSMUSG00000017760	Ctsa	1253.6118	10.617784	3.393133	6.54E-05	0.004272
57	ENSMUSG00000003435	Supt5	710.91063	10.58427301	3.2046	3.79E-05	0.002648
58	ENSMUSG00000027180	Fbxo3	1420.4405	10.52147761	3.854959	0.000229	0.011505
59	ENSMUSG00000021936	Mapk8	623.30225	10.4874172	3.136665	3.52E-05	0.002509
60	ENSMUSG00000076617	Ighm	1344.3249	10.44433006	3.844214	0.000248	0.011881
61	ENSMUSG00000052727	Map1b	1341.943	10.44185302	3.84387	0.000249	0.011881
62	ENSMUSG00000015176	Nolc1	607.83261	10.44158044	3.139637	3.86E-05	0.002652
63	ENSMUSG00000061904	Slc25a3	1408.104	10.39961534	3.312157	7.29E-05	0.004532
64	ENSMUSG00000020349	Ppp2ca	1234.2688	10.32552872	3.827775	0.000281	0.012356
65	ENSMUSG00000030884	Uqcrc2	574.51351	10.29619805	3.170981	5.53E-05	0.003669
66	ENSMUSG00000040592	Cd79b	1311.1752	10.29040067	3.324832	9.01E-05	0.005347
67	ENSMUSG00000041028	Ghitm	1189.6497	10.27462777	3.820777	0.000296	0.012867
68	ENSMUSG00000037548	H2-DMb2	1138.4871	10.21410113	3.812493	0.000315	0.013548
69	ENSMUSG00000020143	Dock2	1060.1708	10.11652169	3.799234	0.000348	0.014436
70	ENSMUSG00000061032	Rrp1	1059.0936	10.11513485	3.799046	0.000349	0.014436
71	ENSMUSG00000054604	Cggbp1	1039.5626	10.08976435	3.79562	0.000358	0.014436
72	ENSMUSG00000042228	Lyn	1019.2554	10.06292424	3.792005	0.000368	0.014562
73	ENSMUSG00000003037	Rab8a	1009.5314	10.04990168	3.790254	0.000373	0.014612
74	ENSMUSG00000017561	Crlf3	976.46751	10.00475444	3.784205	0.000391	0.015011
75	ENSMUSG00000005846	Rsl1d1	459.67314	9.981433831	3.148291	8.70E-05	0.005244
76	ENSMUSG00000046324	Ermp1	502.20696	9.965621434	3.236593	0.000117	0.006535
77	ENSMUSG00000038650	Rnh1	896.33863	9.889322647	3.768878	0.000441	0.016753
78	ENSMUSG00000027078	Ube2l6	1038.0466	9.874960222	3.328143	0.000173	0.009156
79	ENSMUSG00000038116	Phf20	808.10889	9.846463777	3.140057	0.000106	0.006102
80	ENSMUSG00000031781	Ciapin1	1009.643	9.819483983	3.334304	0.000191	0.009954
81	ENSMUSG00000019872	Smpdl3a	849.17134	9.816924103	3.759375	0.000475	0.016863
82	ENSMUSG00000022425	Enpp2	839.43059	9.801520052	3.757365	0.000483	0.016863
83	ENSMUSG00000016256	Ctsz	975.04924	9.789113784	3.320742	0.000193	0.009954
84	ENSMUSG00000018965	Ywhah	831.23601	9.788434587	3.75566	0.000489	0.016863
85	ENSMUSG00000031422	Morf4l2	820.46207	9.786642407	2.782866	3.12E-05	0.002263
86	ENSMUSG00000032171	Pin1	827.01227	9.781643582	3.754777	0.000493	0.016863
87	ENSMUSG00000019710	Mrpl24	385.2215	9.715666465	3.143455	0.000132	0.007201
88	ENSMUSG00000006273	Atp6v1b2	781.17329	9.705850235	3.744972	0.000533	0.016863

89	ENSMUSG00000018654	Ikzf1	365.18627	9.70139762	3.104222	0.00012	0.006631
90	ENSMUSG00000032869	Psmf1	767.96873	9.683267532	3.742071	0.000545	0.016863
91	ENSMUSG00000066979	Bub3	491.96014	9.677986707	3.376734	0.000261	0.012043
92	ENSMUSG00000019295	Tmem129	763.26749	9.675142574	3.74103	0.00055	0.016863
93	ENSMUSG00000028049	Scamp3	750.65551	9.653116444	3.738213	0.000563	0.016863
94	ENSMUSG00000078652	Psme3	1249.1389	9.651139531	3.25431	0.0002	0.010178
95	ENSMUSG00000028793	Rnf19b	480.90993	9.650054337	3.372474	0.000269	0.01227
96	ENSMUSG00000039456	Morc3	736.28542	9.627607267	3.734963	0.000578	0.016863
97	ENSMUSG00000026872	Zeb2	453.65176	9.627487644	3.335081	0.000255	0.012009
98	ENSMUSG00000021577	Sdha	351.72396	9.620927615	3.117813	0.000143	0.007661
99	ENSMUSG00000094483	Purb	725.68889	9.608503966	3.732537	0.000589	0.016863
100	ENSMUSG00000022814	Umps	724.67519	9.606664138	3.732303	0.00059	0.016863
101	ENSMUSG00000023175	Bsg	724.59684	9.606520685	3.732285	0.000591	0.016863
102	ENSMUSG00000078653	Cntd1	724.40423	9.606170817	3.732241	0.000591	0.016863
103	ENSMUSG00000032187	Smarca4	712.44236	9.584262079	3.729468	0.000604	0.016863
104	ENSMUSG00000024673	Ms4a1	700.07785	9.561262999	3.726568	0.000619	0.016863
105	ENSMUSG00000005233	Spc25	699.47916	9.560139678	3.726427	0.00062	0.016863
106	ENSMUSG00000021895	Arhgef3	430.78111	9.553890865	3.331275	0.000281	0.012356
107	ENSMUSG00000030224	Strap	688.00994	9.538454039	3.723702	0.000634	0.016863
108	ENSMUSG00000093467	NA	686.78156	9.536112905	3.723409	0.000635	0.016863
109	ENSMUSG00000026708	Cenpl	683.68873	9.530198805	3.722668	0.000639	0.016863
110	ENSMUSG00000020664	Dld	680.70558	9.524470541	3.72195	0.000643	0.016863
111	ENSMUSG00000076586	Igkv8-21	678.64162	9.520495876	3.721453	0.000646	0.016863
112	ENSMUSG00000019792	Trmt11	664.6618	9.49327149	3.718056	0.000664	0.016863
113	ENSMUSG00000028459	Cd72	657.0679	9.478263147	3.716191	0.000674	0.016863
114	ENSMUSG00000031226	Pbdc1	654.60913	9.473370226	3.715583	0.000678	0.016863
115	ENSMUSG00000031818	Cox4i1	651.36922	9.466896246	3.71478	0.000682	0.016863
116	ENSMUSG00000031774	Psme3ip1	649.75343	9.463655917	3.714379	0.000685	0.016863
117	ENSMUSG00000050565	Tor1aip2	647.79697	9.459725213	3.713892	0.000687	0.016863
118	ENSMUSG00000048521	Cxcr6	647.49457	9.459115183	3.713817	0.000688	0.016863
119	ENSMUSG00000057367	Birc2	647.37474	9.458873963	3.713787	0.000688	0.016863
120	ENSMUSG00000024841	Eif1ad	646.93575	9.457990267	3.713677	0.000689	0.016863
121	ENSMUSG00000062593	Lilrb4a	644.96772	9.454019385	3.713186	0.000692	0.016863
122	ENSMUSG00000048534	Jaml	641.49031	9.446974948	3.712316	0.000697	0.016863
123	ENSMUSG00000026489	Coq8a	638.35986	9.440603643	3.711529	0.000701	0.016863
124	ENSMUSG00000091423	NA	628.90056	9.421175342	3.709136	0.000715	0.016863
125	ENSMUSG00000027750	Postn	624.41002	9.411860519	3.707992	0.000722	0.016863
126	ENSMUSG00000020326	Ccng1	611.93077	9.385644939	3.704781	0.000742	0.016863
127	ENSMUSG00000059734	Ndufs8	609.77714	9.381070593	3.704222	0.000746	0.016863
128	ENSMUSG00000049354	Dcaf7	383.47064	9.376967034	3.33	0.000361	0.014436
129	ENSMUSG00000019998	Stx7	607.4487	9.376110689	3.703617	0.00075	0.016863
130	ENSMUSG00000029439	Sfswap	606.8895	9.374916442	3.703471	0.00075	0.016863
131	ENSMUSG00000006024	Napa	602.20928	9.364882628	3.702249	0.000758	0.016863
132	ENSMUSG00000005687	Bcas2	596.28952	9.352088216	3.700693	0.000768	0.016863
133	ENSMUSG00000036398	Ppp1r11	592.94574	9.34481031	3.69981	0.000774	0.016863
134	ENSMUSG00000024985	Tcf7l2	591.36333	9.341353211	3.69939	0.000777	0.016863
135	ENSMUSG00000003379	Cd79a	589.29064	9.33681132	3.69884	0.000781	0.016863

136	ENSMUSG00000021595	Nsun2	588.47753	9.335025678	3.698624	0.000782	0.016863
137	ENSMUSG00000022876	Samsn1	584.0121	9.325179528	3.697433	0.00079	0.016863
138	ENSMUSG00000048277	Syngr2	583.78734	9.324682052	3.697373	0.00079	0.016863
139	ENSMUSG00000018008	Cyth4	568.91475	9.291370033	3.69336	0.000818	0.016863
140	ENSMUSG00000030342	Cd9	568.75107	9.290999677	3.693315	0.000818	0.016863
141	ENSMUSG00000038646	Ramac	287.93749	9.280071077	3.14146	0.000261	0.012043
142	ENSMUSG00000063439	B9d2	561.10625	9.273559334	3.691224	0.000833	0.016863
143	ENSMUSG00000070697	Utp3	560.27669	9.271654624	3.690996	0.000835	0.016863
144	ENSMUSG00000027447	Cst3	560.26852	9.271635161	3.690994	0.000835	0.016863
145	ENSMUSG00000002007	Srpk3	553.98427	9.257116492	3.68926	0.000848	0.016863
146	ENSMUSG00000030982	Vps35l	317.22954	9.24274232	3.245325	0.000359	0.014436
147	ENSMUSG00000051232	Tmem199	544.09415	9.233958027	3.686503	0.000868	0.016863
148	ENSMUSG00000000278	Scpep1	541.43356	9.227660714	3.685755	0.000874	0.016863
149	ENSMUSG00000011884	Gltp	539.29106	9.222570971	3.685152	0.000878	0.016863
150	ENSMUSG00000002550	Uck1	538.93309	9.221718502	3.685051	0.000879	0.016863
151	ENSMUSG00000040681	Hmgn1	536.98132	9.217061166	3.684499	0.000883	0.016863
152	ENSMUSG00000026309	Ilkap	695.53695	9.215129094	2.845296	0.000115	0.006517
153	ENSMUSG00000025935	Tram1	517.13906	9.198418314	3.395437	0.000534	0.016863
154	ENSMUSG00000038421	Fcrla	525.69833	9.189835239	3.681285	0.000909	0.016863
155	ENSMUSG00000021635	Rad17	272.82208	9.164989087	3.162052	0.000328	0.013941
156	ENSMUSG00000020537	Drg2	515.40992	9.164535139	3.678313	0.000933	0.016863
157	ENSMUSG00000006998	Psmd2	513.57023	9.15996115	3.677778	0.000937	0.016863
158	ENSMUSG00000048076	Arf1	513.55483	9.159924419	3.677774	0.000937	0.016863
159	ENSMUSG00000039615	Stub1	512.08709	9.156265406	3.677345	0.000941	0.016863
160	ENSMUSG00000003033	Ap1m1	669.30468	9.156218446	3.349151	0.000514	0.016863
161	ENSMUSG00000069539	Scyl2	511.89122	9.155777251	3.677288	0.000941	0.016863
162	ENSMUSG00000006127	Inpp5k	511.57214	9.154979406	3.677195	0.000942	0.016863
163	ENSMUSG00000006095	Tbcb	507.51673	9.144810016	3.676007	0.000952	0.016863
164	ENSMUSG00000025484	Bet1l	501.18504	9.128781942	3.67414	0.000968	0.016863
165	ENSMUSG00000024817	Uhrf2	494.57024	9.111836582	3.672173	0.000985	0.016863
166	ENSMUSG00000023020	Cox14	489.39213	9.098425838	3.670621	0.000998	0.016863
167	ENSMUSG00000032382	Snx1	489.24636	9.098047097	3.670578	0.000999	0.016863
168	ENSMUSG00000038280	Ostm1	488.56235	9.096265668	3.670372	0.001001	0.016863
169	ENSMUSG00000031314	Taf1	297.37435	9.08786405	3.278273	0.000484	0.016863
170	ENSMUSG00000026810	Dpm2	481.77406	9.078456258	3.668319	0.001019	0.016863
171	ENSMUSG00000025888	Casp1	479.56894	9.072622587	3.667648	0.001025	0.016863
172	ENSMUSG00000019087	Atp6ap1	479.3081	9.071931467	3.667568	0.001026	0.016863
173	ENSMUSG00000079614	Seh1l	478.89984	9.0708474	3.667444	0.001027	0.016863
174	ENSMUSG00000020153	Ndufs7	475.18403	9.060947707	3.666308	0.001038	0.016863
175	ENSMUSG00000033885	Pxk	474.52486	9.059184057	3.666105	0.00104	0.016863
176	ENSMUSG00000002010	Idh3g	472.06313	9.052575791	3.665349	0.001047	0.016863
177	ENSMUSG00000029518	Rab35	471.2193	9.05030567	3.665089	0.001049	0.016863
178	ENSMUSG00000047180	Neurl3	470.49028	9.048336855	3.664864	0.001051	0.016863
179	ENSMUSG00000026111	Unc50	465.26739	9.034172217	3.663247	0.001067	0.016863
180	ENSMUSG00000039621	Prex1	311.47095	9.032528268	3.346879	0.000605	0.016863
181	ENSMUSG00000030942	Thumpd1	461.44846	9.023719155	3.662057	0.001078	0.016863
182	ENSMUSG00000049103	Ccr2	602.25063	9.017950985	3.338501	0.000607	0.016863

183	ENSMUSG00000053931	Cnn3	459.10068	9.017253902	3.661322	0.001086	0.016863
184	ENSMUSG00000037649	H2-DMa	456.85041	9.011031159	3.660616	0.001093	0.016863
185	ENSMUSG00000001098	Kctd10	453.67465	9.002196337	3.659615	0.001103	0.016863
186	ENSMUSG00000033411	Ctdspl2	452.44463	8.998759381	3.659226	0.001106	0.016863
187	ENSMUSG00000076577	Igkv8-30	452.38606	8.998597185	3.659207	0.001107	0.016863
188	ENSMUSG00000022037	Clu	451.82514	8.997026624	3.65903	0.001108	0.016863
189	ENSMUSG00000017144	Rnd3	286.03454	8.99659828	3.297147	0.000573	0.016863
190	ENSMUSG00000028437	Ubap1	451.22128	8.995333174	3.658838	0.00111	0.016863
191	ENSMUSG00000036572	Upf3b	451.02178	8.99477362	3.658775	0.001111	0.016863
192	ENSMUSG00000024104	Washc2	449.72783	8.99113872	3.658364	0.001115	0.016863
193	ENSMUSG00000054720	Lrrc8c	449.41734	8.990265503	3.658266	0.001116	0.016863
194	ENSMUSG00000036672	Cenpt	449.27877	8.989874176	3.658222	0.001117	0.016863
195	ENSMUSG00000020130	Tbc1d15	1520.5655	8.986677112	2.618081	6.98E-05	0.004409
196	ENSMUSG00000057133	Chd6	403.90156	8.984820503	3.578566	0.000989	0.016863
197	ENSMUSG00000029038	Ssu72	446.75847	8.98275936	3.657419	0.001125	0.016863
198	ENSMUSG00000049550	Clip1	403.90849	8.982299717	3.579857	0.000994	0.016863
199	ENSMUSG00000031007	Atp6ap2	404.29363	8.974295092	3.584651	0.001012	0.016863
200	ENSMUSG00000050043	Tmx2	443.32446	8.973005166	3.656321	0.001136	0.016863
201	ENSMUSG00000026581	Sell	442.79549	8.968951297	3.394048	0.000725	0.016863
202	ENSMUSG00000031662	Snx20	439.64901	8.962489141	3.65514	0.001148	0.016863
203	ENSMUSG00000031479	Vps36	439.56717	8.962254311	3.655113	0.001149	0.016863
204	ENSMUSG00000025981	Coq10b	439.28025	8.961429167	3.655021	0.00115	0.016863
205	ENSMUSG00000030095	Tmem43	273.56701	8.95006918	3.286587	0.000597	0.016863
206	ENSMUSG00000019132	BC005537	391.19365	8.939835631	3.576585	0.001042	0.016863
207	ENSMUSG00000030879	Mrpl17	403.14001	8.938878225	3.600314	0.001084	0.016863
208	ENSMUSG00000037062	Sh3glb1	389.4886	8.932771048	3.576789	0.001052	0.016863
209	ENSMUSG00000027671	Actl6a	392.55966	8.927420627	3.585572	0.001074	0.016863
210	ENSMUSG00000004934	Pias4	396.0742	8.920845789	3.595769	0.0011	0.016863
211	ENSMUSG00000004552	Ctse	394.37717	8.920432337	3.592668	0.001095	0.016863
212	ENSMUSG00000027613	Eif6	541.4271	8.916621103	3.323844	0.000676	0.016863
213	ENSMUSG00000030263	Irag2	284.90531	8.906954023	3.343472	0.000712	0.016863
214	ENSMUSG00000059811	Atl2	238.78067	8.906017111	3.199415	0.000523	0.016863
215	ENSMUSG00000031021	Tmem9b	418.56303	8.900534375	3.648237	0.001224	0.016881
216	ENSMUSG00000016541	Atxn10	418.33351	8.899844506	3.648161	0.001225	0.016881
217	ENSMUSG00000033916	Chmp2a	378.6455	8.898164246	3.572537	0.00109	0.016863
218	ENSMUSG00000029687	Ezh2	378.52761	8.897919479	3.572421	0.00109	0.016863
219	ENSMUSG00000022270	Retreg1	377.96841	8.894424444	3.573056	0.001096	0.016863
220	ENSMUSG00000020817	Rabep1	384.76753	8.893614868	3.587212	0.001122	0.016863
221	ENSMUSG00000070319	Eif3g	379.09003	8.893147954	3.575992	0.001103	0.016863
222	ENSMUSG00000014294	Ndufa2	379.55142	8.892998232	3.577006	0.001105	0.016863
223	ENSMUSG00000037845	Fdxacb1	415.82033	8.892265534	3.647323	0.001234	0.016881
224	ENSMUSG00000042474	Fcmr	415.12013	8.890146802	3.647089	0.001237	0.016881
225	ENSMUSG00000036768	Kif15	413.25428	8.884482484	3.646465	0.001244	0.01692
226	ENSMUSG00000024359	Hspa9	393.68225	8.879196577	3.612057	0.001188	0.016881
227	ENSMUSG00000038880	Mrps34	380.64261	8.879097603	3.586262	0.00114	0.016863
228	ENSMUSG00000038241	Cep250	269.23708	8.876758054	3.31441	0.000699	0.016863
229	ENSMUSG00000036599	Chst12	410.24944	8.875314165	3.645455	0.001256	0.016998

230	ENSMUSG00000025162	Csnk1d	409.5879	8.873286695	3.645232	0.001259	0.016998
231	ENSMUSG00000029759	Pon3	372.44222	8.872798476	3.57269	0.001124	0.016863
232	ENSMUSG00000020457	Drg1	371.16667	8.861786471	3.575643	0.001145	0.016863
233	ENSMUSG00000055943	Emc7	368.86656	8.85694437	3.573321	0.001148	0.016863
234	ENSMUSG00000025287	Acot9	366.76666	8.854930143	3.569951	0.001144	0.016863
235	ENSMUSG00000020397	Med7	364.4042	8.845213594	3.569924	0.001158	0.016881
236	ENSMUSG00000070056	Mfhas1	368.11476	8.84364911	3.578506	0.001176	0.016881
237	ENSMUSG00000029204	Rhoh	372.31459	8.829825666	3.594197	0.001226	0.016881
238	ENSMUSG00000025212	Sfxn3	360.1482	8.829744868	3.568768	0.001178	0.016881
239	ENSMUSG00000027018	Hat1	361.05742	8.824049761	3.573601	0.001195	0.016881
240	ENSMUSG00000021814	Anxa7	361.37713	8.822062189	3.575291	0.001201	0.016881
241	ENSMUSG00000036940	Kdm1a	369.55339	8.821371172	3.592763	0.001236	0.016881
242	ENSMUSG00000003814	Calr	525.53954	8.819375781	3.337468	0.000791	0.016863
243	ENSMUSG00000002109	Ddb2	356.77594	8.819284311	3.56687	0.001189	0.016881
244	ENSMUSG00000000817	Fasl	356.06235	8.816027898	3.566992	0.001194	0.016881
245	ENSMUSG00000006740	Kif5b	232.48167	8.814593491	3.229729	0.000636	0.016863
246	ENSMUSG00000025616	Usp16	355.45587	8.813915888	3.56676	0.001197	0.016881
247	ENSMUSG00000043421	Hilpda	358.52045	8.808396266	3.576152	0.001223	0.016881
248	ENSMUSG00000024056	Ndc80	372.82305	8.805593006	3.607443	0.001289	0.017101
249	ENSMUSG00000042625	Safb2	369.43393	8.792267733	3.078591	0.00046	0.016863
250	ENSMUSG00000015314	Slamf6	349.52359	8.790338312	3.565871	0.00123	0.016881
251	ENSMUSG00000021500	Ddx46	356.56474	8.785923641	3.583372	0.001271	0.017066
252	ENSMUSG00000032220	Myo1e	487.00379	8.781898497	3.312917	0.000792	0.016863
253	ENSMUSG00000037486	Asxl2	366.06327	8.766390286	3.61322	0.001362	0.01758
254	ENSMUSG00000024335	Brd2	374.51543	8.761426176	3.092481	0.000499	0.016863
255	ENSMUSG00000056941	Commd7	485.02863	8.758873624	3.306686	0.000806	0.016863
256	ENSMUSG00000072704	Smim10l1	344.63259	8.758483926	3.571323	0.001289	0.017101
257	ENSMUSG00000025736	Jmjd8	338.53082	8.748252952	3.562896	0.001288	0.017101
258	ENSMUSG00000004296	Il12b	338.6921	8.746877168	3.563962	0.001293	0.017101
259	ENSMUSG00000027665	Pik3ca	345.50245	8.722131225	3.064001	0.000492	0.016863
260	ENSMUSG00000079036	Alkbh1	331.08437	8.721513152	3.559579	0.001324	0.017385
261	ENSMUSG00000001151	Pcnt	330.66637	8.719394031	3.559699	0.001327	0.017385
262	ENSMUSG00000025795	Rassf3	338.03162	8.718550928	3.576903	0.001365	0.01758
263	ENSMUSG00000022234	Cct5	475.7434	8.713094665	3.332322	0.000901	0.016863
264	ENSMUSG00000002058	Unc119	325.60247	8.699595915	3.558064	0.001356	0.01758
265	ENSMUSG00000027981	Rnpc3	210.02663	8.696137868	3.214377	0.000725	0.016863
266	ENSMUSG00000025159	Mms19	365.48996	8.691470559	3.129672	0.000603	0.016863
267	ENSMUSG00000040820	Hlcs	341.8426	8.682163095	3.603798	0.001485	0.017687
268	ENSMUSG00000026117	Zap70	320.64155	8.680373528	3.556217	0.001384	0.01764
269	ENSMUSG00000024334	H2-Oa	323.52022	8.676890079	3.564791	0.001408	0.01764
270	ENSMUSG00000030612	Mrpl46	319.95603	8.676763991	3.556438	0.00139	0.01764
271	ENSMUSG00000038046	Mrm3	319.31155	8.674935197	3.555841	0.001392	0.01764
272	ENSMUSG00000022663	Atg3	336.05808	8.674165308	3.594955	0.001479	0.017687
273	ENSMUSG00000019189	Rnf145	319.55422	8.671142615	3.558361	0.001404	0.01764
274	ENSMUSG00000030619	Eed	317.93449	8.669497155	3.555341	0.0014	0.01764
275	ENSMUSG00000015806	Qdpr	316.98364	8.66506571	3.555335	0.001408	0.01764
276	ENSMUSG00000050199	Lgr4	336.91848	8.6595024	3.604276	0.001526	0.017905

277	ENSMUSG00000097971	NA	314.9908	8.657516268	3.554412	0.001418	0.017687
278	ENSMUSG00000004896	Rrnad1	173.25832	8.650231253	3.081869	0.000571	0.016863
279	ENSMUSG00000005506	Celf1	311.70727	8.644181036	3.553293	0.001439	0.017687
280	ENSMUSG00000028906	Epb41	314.24416	8.641981522	3.560566	0.001459	0.017687
281	ENSMUSG00000056692	Ilrun	310.71647	8.639423854	3.553316	0.001447	0.017687
282	ENSMUSG00000021886	Gpr65	309.36703	8.632637373	3.553497	0.001459	0.017687
283	ENSMUSG00000097052	Snora43	308.98425	8.628591161	3.554632	0.001469	0.017687
284	ENSMUSG00000061132	Blnk	308.12277	8.628536667	3.552544	0.001464	0.017687
285	ENSMUSG00000015437	Gzmb	311.28174	8.627152139	3.560977	0.001486	0.017687
286	ENSMUSG00000062797	Hikeshi	306.45443	8.622504639	3.551525	0.001473	0.017687
287	ENSMUSG00000071715	Ncf4	325.91225	8.621705926	3.598367	0.001582	0.017905
288	ENSMUSG00000028447	Dctn3	305.88767	8.618269121	3.552296	0.001482	0.017687
289	ENSMUSG00000038773	Kdm3b	305.91813	8.617958759	3.55253	0.001483	0.017687
290	ENSMUSG00000059994	Fcrl1	305.49148	8.616278532	3.552336	0.001486	0.017687
291	ENSMUSG00000022772	Senp5	300.8799	8.599411706	3.549481	0.001509	0.017857
292	ENSMUSG00000001157	Gmcl1	317.91718	8.595039922	3.5932	0.00162	0.018126
293	ENSMUSG00000025894	Aasdhppt	299.67836	8.590213362	3.551179	0.00153	0.017905
294	ENSMUSG00000041459	Tardbp	303.89682	8.589806487	3.561939	0.001556	0.017905
295	ENSMUSG00000001029	Icam2	307.93229	8.589134561	3.572223	0.001581	0.017905
296	ENSMUSG00000029840	Mtpn	307.2986	8.587545019	3.571483	0.001583	0.017905
297	ENSMUSG00000003623	Crot	314.54671	8.584291526	3.590644	0.001635	0.018149
298	ENSMUSG00000015575	Atp6v0e	298.89591	8.583686534	3.552554	0.001545	0.017905
299	ENSMUSG00000020755	Sap30bp	303.38678	8.578870398	3.566265	0.001587	0.017905
300	ENSMUSG00000012848	Rps5	301.08412	8.57444849	3.562788	0.001587	0.017905
301	ENSMUSG00000048897	Zfp710	201.91953	8.568132575	3.255742	0.000939	0.016863
302	ENSMUSG00000030707	Coro1a	296.12822	8.565495981	3.554865	0.001585	0.017905
303	ENSMUSG00000052949	Rnf157	294.16082	8.562228745	3.551518	0.001583	0.017905
304	ENSMUSG00000025041	Nt5c2	291.76231	8.561595061	3.545667	0.00157	0.017905
305	ENSMUSG00000056342	Usp34	291.48745	8.556293909	3.547683	0.001585	0.017905
306	ENSMUSG00000097457	NA	289.14249	8.55170723	3.543948	0.001584	0.017905
307	ENSMUSG00000003380	Rabac1	306.63959	8.541756394	3.05666	0.000628	0.016863
308	ENSMUSG00000032575	Manf	294.90936	8.540685773	3.564467	0.001656	0.018149
309	ENSMUSG00000027882	Stxbp3	290.95134	8.53405276	3.557714	0.001652	0.018149
310	ENSMUSG00000085526	NA	284.31219	8.53071696	3.542058	0.00162	0.018126
311	ENSMUSG00000020366	Mapk9	293.13315	8.522104793	3.569425	0.001705	0.018408
312	ENSMUSG00000060591	Ifitm2	283.35789	8.522072952	3.543979	0.001641	0.018149
313	ENSMUSG00000030748	Il4ra	285.52471	8.521819426	3.549841	0.001656	0.018149
314	ENSMUSG00000024910	Ctsw	290.79437	8.516638089	3.56622	0.001708	0.018408
315	ENSMUSG00000021823	Vcl	152.35593	8.514646472	3.055867	0.000651	0.016863
316	ENSMUSG00000032264	Zw10	290.88138	8.513419362	3.568088	0.001719	0.01844
317	ENSMUSG00000001847	Rac1	410.55758	8.508177707	3.313426	0.001135	0.016863
318	ENSMUSG00000056267	Cep70	298.40491	8.503855123	3.592012	0.0018	0.018718
319	ENSMUSG00000032601	Prkar2a	279.93942	8.494378154	3.549109	0.001709	0.018408
320	ENSMUSG00000097125	NA	275.81922	8.491826564	3.539291	0.001689	0.018377
321	ENSMUSG00000032434	Cmtm6	273.73311	8.480956802	3.539197	0.001711	0.018408
322	ENSMUSG00000014763	Fam120b	961.66967	8.478962505	2.845887	0.00039	0.015011
323	ENSMUSG00000024446	Rpp21	274.7211	8.47844999	3.543194	0.001726	0.018471

324	ENSMUSG00000021188	Trip11	291.33996	8.474089001	3.589271	0.001856	0.018762
325	ENSMUSG00000023088	Abcc1	288.68286	8.472012618	3.583511	0.001845	0.018718
326	ENSMUSG00000097643	NA	278.74691	8.470044624	3.558407	0.001783	0.018718
327	ENSMUSG00000041215	Yeats2	274.3921	8.466493634	3.548451	0.001765	0.018718
328	ENSMUSG00000002103	Acp2	272.39836	8.462390193	3.545105	0.001764	0.018718
329	ENSMUSG00000089875	NA	278.65413	8.460492296	3.563048	0.001816	0.018718
330	ENSMUSG00000098973	Mir6236	410.77169	8.458681664	3.34147	0.001273	0.017066
331	ENSMUSG00000030105	Arl8b	277.0549	8.457464126	3.560304	0.001815	0.018718
332	ENSMUSG00000035021	Baz1a	279.00465	8.451682725	3.048039	0.0007	0.016863
333	ENSMUSG00000025024	Smndc1	274.87762	8.449532881	3.558484	0.001827	0.018718
334	ENSMUSG00000024659	Anxa1	265.24037	8.444160428	3.534564	0.001775	0.018718
335	ENSMUSG00000011114	Tbrg1	278.12488	8.443044115	3.570552	0.001873	0.018762
336	ENSMUSG00000076937	Iglc2	269.00492	8.436993666	3.548804	0.001828	0.018718
337	ENSMUSG00000040774	Cept1	268.05666	8.433869626	3.547775	0.001832	0.018718
338	ENSMUSG00000024353	Mzb1	263.55671	8.432757415	3.53569	0.001802	0.018718
339	ENSMUSG00000032596	Uba7	371.82287	8.43057876	3.294395	0.001211	0.016881
340	ENSMUSG00000033307	Mif	265.71233	8.430433644	3.542983	0.001826	0.018718
341	ENSMUSG00000060904	Arl1	265.42582	8.426378898	3.544267	0.001839	0.018718
342	ENSMUSG00000086429	Gt(ROSA)2(	262.4677	8.422394346	3.537947	0.00183	0.018718
343	ENSMUSG00000038671	Arfrp1	258.9961	8.41594576	3.531325	0.001826	0.018718
344	ENSMUSG00000034424	Gcsh	264.55268	8.411394661	3.549525	0.001886	0.018836
345	ENSMUSG00000022895	Ets2	261.62653	8.409045533	3.54244	0.001871	0.018762
346	ENSMUSG00000022043	Trim35	277.17521	8.408056715	3.585825	0.001994	0.019326
347	ENSMUSG00000037971	1110032A0	257.71208	8.407963506	3.531742	0.001844	0.018718
348	ENSMUSG00000014606	Slc25a11	260.42064	8.405875063	3.540631	0.001873	0.018762
349	ENSMUSG00000086324	NA	264.37222	8.401173802	3.033756	0.000726	0.016863
350	ENSMUSG00000043411	Usp48	457.0776	8.399684203	3.058058	0.000771	0.016863
351	ENSMUSG00000051355	Commd1	257.83038	8.375260543	3.548969	0.001965	0.019214
352	ENSMUSG00000038400	Pmepa1	250.56638	8.372666964	3.529031	0.001915	0.019024
353	ENSMUSG00000026074	Map4k4	250.21553	8.370808962	3.52895	0.001919	0.019024
354	ENSMUSG00000006678	Pola1	257.11054	8.369249504	3.549986	0.001982	0.019259
355	ENSMUSG00000022749	Tbc1d23	255.67675	8.368637549	3.546144	0.001972	0.019214
356	ENSMUSG00000057858	Fam204a	138.25383	8.368055264	3.063989	0.000817	0.016863
357	ENSMUSG00000086718	NA	249.78007	8.364719759	3.530806	0.001938	0.019116
358	ENSMUSG00000039568	Ubald1	356.14099	8.361629613	3.299308	0.001334	0.017416
359	ENSMUSG00000000001	Gnai3	247.58923	8.359730414	3.526828	0.001938	0.019116
360	ENSMUSG00000021109	Hif1a	181.19665	8.350150626	3.293268	0.001339	0.017422
361	ENSMUSG00000040029	Ipo8	250.74865	8.332818555	3.550155	0.002068	0.019534
362	ENSMUSG00000023846	NA	151.78463	8.320837809	3.169031	0.001095	0.016863
363	ENSMUSG00000096336	Igkv1-135	260.81552	8.31898582	3.586242	0.00221	0.019646
364	ENSMUSG00000027952	Pmvk	241.25105	8.317672496	3.529351	0.002043	0.019534
365	ENSMUSG00000025104	Hdgfl3	540.41149	8.313398102	2.838716	0.000494	0.016863
366	ENSMUSG00000052477	C130026I2	237.53266	8.312370465	3.520562	0.00203	0.019534
367	ENSMUSG00000079227	Ccr5	239.07359	8.311938331	3.525594	0.002046	0.019534
368	ENSMUSG00000025781	Atp5c1	237.0583	8.303773173	3.523549	0.002059	0.019534
369	ENSMUSG00000029815	Malsu1	236.22144	8.294270813	3.525862	0.002089	0.019534
370	ENSMUSG00000022677	Cep20	235.00761	8.292618781	3.522897	0.002084	0.019534

371	ENSMUSG00000021116	Eif2s1	234.00493	8.287424501	3.522426	0.002095	0.019534
372	ENSMUSG00000037071	Scd1	234.58507	8.286353095	3.52482	0.002105	0.019534
373	ENSMUSG00000031960	Aars	232.99232	8.284205147	3.520883	0.002099	0.019534
374	ENSMUSG00000061207	Stk19	231.36806	8.281585848	3.517053	0.002093	0.019534
375	ENSMUSG00000028953	Abcf2	238.96994	8.280713382	3.541448	0.00217	0.019534
376	ENSMUSG00000028195	Ccn1	249.39268	8.27849326	3.574004	0.002276	0.019684
377	ENSMUSG00000054676	1600014C1	234.47715	8.277939153	3.528846	0.002138	0.019534
378	ENSMUSG00000027668	Mfn1	336.14502	8.27734347	3.286177	0.00145	0.017687
379	ENSMUSG00000029209	Gnpda2	246.05107	8.276612658	3.565065	0.002253	0.019684
380	ENSMUSG00000001674	Ddx18	229.55744	8.271277595	3.516596	0.002117	0.019534
381	ENSMUSG00000054065	Pkp3	237.74423	8.270786068	3.542779	0.002199	0.019646
382	ENSMUSG00000027072	Prg3	231.94689	8.269782612	3.525051	0.002147	0.019534
383	ENSMUSG00000033781	Asb13	228.4479	8.266736605	3.515369	0.002125	0.019534
384	ENSMUSG00000039801	Cplane1	347.2887	8.266617426	3.31742	0.001553	0.017905
385	ENSMUSG00000031429	Psmd10	231.92054	8.26497352	3.527465	0.002166	0.019534
386	ENSMUSG00000028521	Slc35d1	228.22205	8.264368133	3.515871	0.002132	0.019534
387	ENSMUSG00000090112	Shprh	229.01335	8.263831698	3.518717	0.002142	0.019534
388	ENSMUSG00000031158	Timm17b	243.43174	8.262095261	3.564638	0.00229	0.019728
389	ENSMUSG00000045427	Hnrnph2	230.54802	8.260868817	3.525206	0.00217	0.019534
390	ENSMUSG00000029177	Cenpa	235.72527	8.258201407	3.542988	0.002232	0.019646
391	ENSMUSG00000021830	Txndc16	226.85061	8.257978574	3.514733	0.002145	0.019534
392	ENSMUSG00000032463	Faim	226.73014	8.255202138	3.515788	0.002155	0.019534
393	ENSMUSG00000023883	Phf10	226.18582	8.252440063	3.515448	0.002161	0.019534
394	ENSMUSG00000028964	Park7	230.41286	8.251635846	3.529568	0.002207	0.019646
395	ENSMUSG00000050668	Gpatch11	237.32623	8.251281769	3.551538	0.002277	0.019684
396	ENSMUSG00000045038	Prkce	231.481	8.251142711	3.533236	0.002219	0.019646
397	ENSMUSG00000097290	1300002E1	225.16782	8.249007656	3.513896	0.002164	0.019534
398	ENSMUSG00000002625	Akap8l	224.93399	8.248494314	3.513393	0.002164	0.019534
399	ENSMUSG00000032370	Lactb	474.49432	8.248286349	3.036186	0.000906	0.016863
400	ENSMUSG00000036613	Eipr1	235.85357	8.244099354	3.550656	0.002293	0.019728
401	ENSMUSG00000076431	Sox4	240.50914	8.235020967	3.569649	0.002378	0.020028
402	ENSMUSG00000046006	Gapt	221.9333	8.232539539	3.511776	0.0022	0.019646
403	ENSMUSG00000020053	Igf1	219.43092	8.217279473	3.511358	0.002238	0.019646
404	ENSMUSG00000039067	Psmd7	225.33711	8.216687711	3.531268	0.002303	0.019735
405	ENSMUSG00000021738	Atxn7	218.80199	8.215844761	3.509984	0.002237	0.019646
406	ENSMUSG00000030079	Ruvbl1	218.80629	8.214236174	3.51084	0.002244	0.019657
407	ENSMUSG00000022686	B3gnt5	220.19134	8.211722881	3.516817	0.00227	0.019684
408	ENSMUSG00000017652	Cd40	216.94453	8.203080867	3.510353	0.002271	0.019684
409	ENSMUSG00000097289	NA	217.88047	8.199485375	3.515413	0.002297	0.019728
410	ENSMUSG00000026568	Mpc2	220.26564	8.198153815	3.524132	0.002329	0.01987
411	ENSMUSG00000027303	Ptpra	227.86427	8.192310573	3.552055	0.002437	0.020223
412	ENSMUSG00000025245	Lztfl1	224.54668	8.189428437	3.542798	0.002415	0.020223
413	ENSMUSG00000021178	Psmc1	224.52266	8.188294048	3.543309	0.002419	0.020223
414	ENSMUSG00000025001	Hells	216.37278	8.187275052	3.516663	0.002334	0.01987
415	ENSMUSG00000033624	Pdpr	227.34026	8.186279208	3.553476	0.002459	0.020359
416	ENSMUSG00000000078	Klf6	1642.8043	8.185481547	2.598757	0.000276	0.012356
417	ENSMUSG00000097461	NA	232.08613	8.184017245	3.569712	0.00252	0.02039

418	ENSMUSG00000041890	Git2	397.29305	8.179248862	3.076755	0.001091	0.016863
419	ENSMUSG00000063808	Gpatch1	220.07776	8.177025132	3.534479	0.002421	0.020223
420	ENSMUSG00000062646	Ganc	147.70577	8.169589635	3.235629	0.001517	0.017889
421	ENSMUSG00000001627	Ifrd1	211.37281	8.167243189	3.509893	0.002366	0.02001
422	ENSMUSG00000042606	Hirip3	223.1972	8.160745711	3.553185	0.002531	0.02039
423	ENSMUSG00000050957	Insl6	218.93452	8.15929284	3.539845	0.002489	0.02039
424	ENSMUSG00000020484	Xbp1	211.15083	8.150994994	3.517611	0.002437	0.020223
425	ENSMUSG00000062580	Timm17a	116.56148	8.146541805	3.058306	0.001096	0.016863
426	ENSMUSG00000031207	Msn	536.0166	8.143878593	3.051739	0.001084	0.016863
427	ENSMUSG00000028063	Lmna	304.3634	8.143228706	3.300084	0.001757	0.018718
428	ENSMUSG00000057132	Rpgrip1	214.44282	8.136859789	3.536295	0.002541	0.020412
429	ENSMUSG00000026848	Tor1b	204.00925	8.135021342	3.500652	0.002424	0.020223
430	ENSMUSG00000025747	Tyms	203.62126	8.132855458	3.500386	0.002429	0.020223
431	ENSMUSG00000031231	Cox7b	211.536	8.132254993	3.528705	0.002528	0.02039
432	ENSMUSG00000031668	Eif2ak3	234.54325	8.12994348	3.084762	0.001186	0.016881
433	ENSMUSG00000060733	Ipmk	203.31272	8.122194723	3.504866	0.002474	0.02039
434	ENSMUSG00000022914	Brwd1	313.23865	8.119809928	3.320187	0.001871	0.018762
435	ENSMUSG00000018740	Slc25a35	208.89268	8.118816028	3.526487	0.002559	0.020444
436	ENSMUSG00000045409	Trim39	210.91985	8.116930665	3.534524	0.002593	0.020598
437	ENSMUSG00000030447	Cyfip1	201.20222	8.11090195	3.503117	0.0025	0.02039
438	ENSMUSG00000048442	Smim5	204.37412	8.106153346	3.517081	0.002563	0.020444
439	ENSMUSG00000046727	Cystm1	199.08006	8.105075603	3.498381	0.002501	0.02039
440	ENSMUSG00000014453	Blk	211.86591	8.101421357	3.545814	0.00268	0.020848
441	ENSMUSG00000024120	Lrpprc	197.57087	8.094648144	3.498267	0.00253	0.02039
442	ENSMUSG00000040511	Pvr	215.80493	8.094260187	3.562869	0.002763	0.020851
443	ENSMUSG00000054280	Prr14l	203.81582	8.092686736	3.522099	0.002621	0.020693
444	ENSMUSG00000021715	Cwc27	212.43612	8.091663599	3.552799	0.002735	0.020851
445	ENSMUSG00000031431	Tsc22d3	210.01104	8.084739032	3.548035	0.002739	0.020851
446	ENSMUSG00000028955	Vamp3	205.58735	8.082785259	3.533561	0.002692	0.020851
447	ENSMUSG00000029462	Vps29	195.03956	8.081493011	3.495713	0.00256	0.020444
448	ENSMUSG00000024999	Noc3l	207.57236	8.075082294	3.544541	0.002756	0.020851
449	ENSMUSG00000038546	Ranbp9	119.80198	8.073756357	3.125166	0.001389	0.01764
450	ENSMUSG00000030611	Mrps11	199.6345	8.072848503	3.517305	0.002663	0.020848
451	ENSMUSG00000052656	Rnf103	193.4114	8.07180108	3.494658	0.002584	0.02057
452	ENSMUSG00000028124	Gclm	192.36143	8.064458736	3.494529	0.002606	0.020647
453	ENSMUSG00000070427	Il18bp	192.31913	8.063499894	3.494872	0.00261	0.020647
454	ENSMUSG00000029655	N4bp2l2	195.22587	8.057366301	3.509083	0.00268	0.020848
455	ENSMUSG00000032913	Lrig2	193.75082	8.056553457	3.503958	0.002664	0.020848
456	ENSMUSG00000026806	Ddx31	190.55385	8.054889681	3.492642	0.002628	0.020704
457	ENSMUSG00000026355	Mcm6	197.29841	8.053184029	3.518976	0.00273	0.020851
458	ENSMUSG00000025163	Cd7	204.41384	8.052058188	3.545315	0.002831	0.021022
459	ENSMUSG00000031783	Polr2c	190.96968	8.051700047	3.495922	0.002649	0.020832
460	ENSMUSG00000030538	Cib1	193.76266	8.049660428	3.507622	0.002699	0.020851
461	ENSMUSG00000015597	Zfp318	193.61307	8.048655939	3.50758	0.002701	0.020851
462	ENSMUSG00000058427	Cxcl2	200.57996	8.048081806	3.533639	0.0028	0.021007
463	ENSMUSG00000017146	Brca1	200.37959	8.042388833	3.535867	0.002827	0.021022
464	ENSMUSG00000039599	Fam149b	190.64862	8.038040991	3.501875	0.002713	0.020851

465	ENSMUSG00000016520	Lnx2	192.93318	8.037344344	3.510937	0.002749	0.020851
466	ENSMUSG00000034311	Kif4	204.97045	8.036441236	3.555335	0.00292	0.021331
467	ENSMUSG00000066456	Hmgn3	191.83529	8.03523046	3.50788	0.002744	0.020851
468	ENSMUSG00000003746	Man1a	186.97647	8.033437894	3.490058	0.002683	0.020848
469	ENSMUSG00000032253	Phip	188.8609	8.032948971	3.497661	0.002713	0.020851
470	ENSMUSG00000024613	Tcof1	278.47062	8.026507954	3.297586	0.00202	0.019529
471	ENSMUSG00000022637	Cblb	188.28788	8.022191987	3.501097	0.002759	0.020851
472	ENSMUSG00000021880	Rnase6	191.49886	8.02140625	3.513842	0.00281	0.021022
473	ENSMUSG00000024789	Jak2	188.04218	8.017698253	3.502504	0.002779	0.020887
474	ENSMUSG00000027884	Clcc1	271.7392	8.01695376	3.268928	0.001947	0.01916
475	ENSMUSG00000064493	Snora28	183.77973	8.011730245	3.488886	0.002746	0.020851
476	ENSMUSG00000032612	Usp4	183.59211	8.011643356	3.488184	0.002744	0.020851
477	ENSMUSG00000031004	Mki67	197.49938	8.010449416	3.541926	0.002954	0.021445
478	ENSMUSG00000028563	Tm2d1	188.29414	8.007705662	3.508724	0.002834	0.021022
479	ENSMUSG00000027162	Lin7c	278.06359	8.005555785	3.292922	0.002056	0.019534
480	ENSMUSG00000048410	Zfp407	184.65155	7.993164154	3.502125	0.002856	0.021062
481	ENSMUSG00000066621	Tecpr1	273.32745	7.99241532	3.287183	0.002069	0.019534
482	ENSMUSG00000022139	Mbnl2	183.28619	7.990220622	3.498259	0.00285	0.021061
483	ENSMUSG00000026436	Elk4	284.39145	7.98517622	3.321862	0.002209	0.019646
484	ENSMUSG00000017188	Coa3	177.63499	7.974537712	3.483675	0.002844	0.021056
485	ENSMUSG00000002833	Hdgfl2	181.35161	7.972693634	3.499754	0.002913	0.021331
486	ENSMUSG00000063972	Nr6a1	185.83904	7.968057765	3.519931	0.003009	0.02166
487	ENSMUSG00000010097	Nxf1	177.89647	7.967547432	3.488444	0.002886	0.021206
488	ENSMUSG00000028851	Nudc	177.99101	7.962139669	3.49169	0.002916	0.021331
489	ENSMUSG00000002658	Gtf2f1	185.26969	7.95524871	3.524392	0.00307	0.021901
490	ENSMUSG00000039828	Wdr70	100.36248	7.953211874	3.054671	0.00141	0.01764
491	ENSMUSG00000079179	Rab10os	254.4258	7.95053913	3.271357	0.002121	0.019534
492	ENSMUSG00000038957	Edc3	254.27618	7.947433566	3.272687	0.002134	0.019534
493	ENSMUSG00000033918	Parl	172.31976	7.941168539	3.479166	0.002936	0.021412
494	ENSMUSG00000062210	Tnfaip8	188.5183	7.940500203	3.544568	0.003204	0.022339
495	ENSMUSG00000026790	Odf2	186.76969	7.939857402	3.01515	0.00132	0.017385
496	ENSMUSG00000030189	Ybx3	172.72847	7.938596659	3.482259	0.002957	0.021445
497	ENSMUSG00000072235	Tuba1a	171.84735	7.936261811	3.479766	0.002955	0.021445
498	ENSMUSG00000020694	Tlk2	174.82428	7.934604298	3.493152	0.003015	0.021661
499	ENSMUSG00000034430	Zxdc	174.27116	7.931859221	3.492293	0.003021	0.021664
500	ENSMUSG00000058655	Eif4b	181.62797	7.929235267	3.523618	0.003158	0.022202
501	ENSMUSG00000031756	Cenpn	184.65047	7.927555765	3.536378	0.003217	0.022339
502	ENSMUSG00000029723	Spacdr	106.28449	7.926171823	3.117985	0.001659	0.018149
503	ENSMUSG00000039206	Daglb	169.64811	7.923920895	3.476918	0.002985	0.021567
504	ENSMUSG00000031264	Btk	169.51771	7.92029807	3.478279	0.003003	0.021656
505	ENSMUSG00000027099	Mtx2	181.74219	7.919153947	3.529322	0.003217	0.022339
506	ENSMUSG00000056536	Pign	174.69819	7.917489947	3.501644	0.003108	0.022052
507	ENSMUSG00000029389	Ddx55	172.52964	7.915876866	3.49343	0.00308	0.021931
508	ENSMUSG00000024384	Iws1	116.83077	7.915740398	3.199475	0.001956	0.019198
509	ENSMUSG00000031928	Mre11a	169.89685	7.910239827	3.485236	0.003066	0.021901
510	ENSMUSG00000027652	Ralgapb	173.23498	7.909411873	3.4998	0.003129	0.022118
511	ENSMUSG00000015149	Sirt2	940.00183	7.90877891	2.928343	0.001131	0.016863

512	ENSMUSG00000025825	Iscu	167.16581	7.906412824	3.475471	0.003039	0.021755
513	ENSMUSG00000020709	Adap2	178.00438	7.905452722	3.521497	0.003234	0.022339
514	ENSMUSG00000025873	Faf2	181.42247	7.901006286	3.537479	0.003318	0.02271
515	ENSMUSG00000033721	Vav3	166.50707	7.894348196	3.478988	0.003095	0.021998
516	ENSMUSG00000002228	Ppm1j	172.20881	7.889489365	3.505971	0.003226	0.022339
517	ENSMUSG00000066952	Myo1h	162.72092	7.877322989	3.471313	0.003123	0.022113
518	ENSMUSG00000057098	Ebf1	1627.6319	7.876723411	2.610415	0.000481	0.016863
519	ENSMUSG00000024218	Taf11	163.80498	7.876701644	3.476475	0.003147	0.022202
520	ENSMUSG00000059820	Nkapd1	166.7094	7.87471842	3.490276	0.003212	0.022339
521	ENSMUSG00000022711	Pmm2	171.64567	7.872567254	3.512489	0.003316	0.02271
522	ENSMUSG00000028382	Ptbp3	246.09511	7.871629526	3.275856	0.002352	0.019959
523	ENSMUSG00000006299	Aamp	108.43346	7.867402355	3.169385	0.001969	0.019214
524	ENSMUSG00000031146	Plp2	164.71503	7.865810573	3.486272	0.003228	0.022339
525	ENSMUSG00000002052	Supt6	167.3694	7.862778747	3.499436	0.003295	0.022639
526	ENSMUSG00000038811	Gngt2	162.68161	7.857113105	3.481876	0.00324	0.022339
527	ENSMUSG00000035623	Rsf1	424.47114	7.852245052	2.867971	0.001058	0.016863
528	ENSMUSG00000035171	1110059E2	173.32851	7.851890141	3.530285	0.003471	0.023314
529	ENSMUSG00000031529	Tnks	160.37355	7.850360348	3.475096	0.003235	0.022339
530	ENSMUSG00000030417	Pdcd5	159.59889	7.849383051	3.472097	0.003226	0.022339
531	ENSMUSG00000025326	Ube3a	88.480871	7.848088597	3.016674	0.001497	0.017764
532	ENSMUSG00000026655	Fam107b	297.29153	7.844236111	3.045599	0.001598	0.017981
533	ENSMUSG00000021699	Pde4d	102.5211	7.844015835	3.138924	0.001917	0.019024
534	ENSMUSG00000061518	Cox5b	162.12219	7.84145013	3.487679	0.003323	0.02271
535	ENSMUSG00000022312	Eif3h	244.09688	7.837761434	3.288987	0.002502	0.02039
536	ENSMUSG00000079477	Rab7	244.39332	7.83693795	3.29038	0.00251	0.02039
537	ENSMUSG00000056216	Cebpg	346.33154	7.830691425	3.246553	0.002355	0.019959
538	ENSMUSG00000062939	Stat4	158.68431	7.827057366	3.479797	0.003342	0.022751
539	ENSMUSG00000049516	Spty2d1	157.55014	7.822552636	3.476997	0.003346	0.022751
540	ENSMUSG00000030256	Bhlhe41	155.0225	7.815080895	3.469255	0.00334	0.022751
541	ENSMUSG00000097392	Thoc2l	163.06764	7.811291628	3.507764	0.003527	0.023375
542	ENSMUSG00000068882	Ssb	2052.5273	7.805160234	2.616658	0.000552	0.016863
543	ENSMUSG00000030313	Dennd5b	166.38658	7.803500743	3.526264	0.003643	0.023545
544	ENSMUSG00000095007	Igkv12-41	160.80212	7.801278285	3.502982	0.003545	0.023375
545	ENSMUSG00000031578	Mak16	531.98455	7.799759061	2.967348	0.001436	0.017687
546	ENSMUSG00000036698	Ago2	157.84853	7.799301658	3.490687	0.003497	0.023375
547	ENSMUSG00000030287	Itpr2	150.8628	7.792472308	3.461602	0.003391	0.023015
548	ENSMUSG00000034330	Plcg2	151.14737	7.789471867	3.464573	0.003416	0.023104
549	ENSMUSG00000003873	Bax	158.98806	7.787966168	3.501843	0.003592	0.023485
550	ENSMUSG00000024921	Smarca2	148.98182	7.778963727	3.45973	0.003434	0.023124
551	ENSMUSG00000004667	Polr2e	148.95915	7.778952249	3.459626	0.003434	0.023124
552	ENSMUSG00000034361	Cpne2	83.779535	7.774943659	3.017839	0.001653	0.018149
553	ENSMUSG00000028149	Rap1gds1	151.65215	7.770477793	3.477106	0.003547	0.023375
554	ENSMUSG00000035203	Epn1	150.63115	7.76775405	3.473677	0.003542	0.023375
555	ENSMUSG00000022565	Plec	149.12107	7.764555956	3.468108	0.003529	0.023375
556	ENSMUSG00000053470	Kdm3a	385.75119	7.763849684	3.237766	0.002516	0.02039
557	ENSMUSG00000026239	Pde6d	150.46725	7.761968893	3.475974	0.003576	0.023455
558	ENSMUSG00000039294	Cybc1	149.39184	7.761692359	3.470941	0.003553	0.023375

559	ENSMUSG00000028884	Rpa2	151.03293	7.759750181	3.479858	0.003603	0.023514
560	ENSMUSG00000071653	1810009A1	148.41483	7.757609578	3.468384	0.003558	0.023375
561	ENSMUSG00000079427	Mthfsl	146.81301	7.756897416	3.460918	0.003526	0.023375
562	ENSMUSG00000021764	Ndufs4	149.40004	7.752748891	3.475743	0.003612	0.023532
563	ENSMUSG00000028744	Slc66a1	158.85402	7.75176028	3.520224	0.003826	0.023898
564	ENSMUSG00000024515	Smad4	156.25833	7.750793255	3.508984	0.003777	0.023816
565	ENSMUSG00000022617	Chkb	144.39355	7.745659713	3.454907	0.003542	0.023375
566	ENSMUSG00000029729	Zkscan1	152.57947	7.743260071	3.495906	0.003746	0.023794
567	ENSMUSG00000040054	Baz2a	153.19049	7.739997704	3.500487	0.003781	0.023816
568	ENSMUSG00000040584	Abcb1a	222.85116	7.739409614	3.291032	0.002821	0.021022
569	ENSMUSG00000028745	Capzb	146.77932	7.738648524	3.4705	0.003644	0.023545
570	ENSMUSG00000027692	Tnik	150.36473	7.737445051	3.488491	0.003734	0.023794
571	ENSMUSG00000052031	Tagap1	96.791096	7.735466487	3.15884	0.002277	0.019684
572	ENSMUSG00000016344	Ppdpf	146.70764	7.724457618	3.477705	0.003736	0.023794
573	ENSMUSG00000039148	Sart1	141.76639	7.723488379	3.453513	0.003624	0.023538
574	ENSMUSG00000041629	Fam104a	142.1088	7.723135826	3.455448	0.003635	0.023545
575	ENSMUSG00000018848	Rars	148.89245	7.721477846	3.489888	0.003807	0.023894
576	ENSMUSG00000030203	Dusp16	100.65816	7.719626684	3.198999	0.002489	0.02039
577	ENSMUSG00000027650	Tti1	150.53785	7.71649634	3.500384	0.00388	0.024153
578	ENSMUSG00000085335	NA	88.280455	7.704924494	3.104238	0.002143	0.019534
579	ENSMUSG00000036246	Gmip	139.73577	7.698536261	3.456476	0.003741	0.023794
580	ENSMUSG00000040848	Sft2d2	139.37541	7.696115883	3.455914	0.003748	0.023794
581	ENSMUSG00000037960	Card19	148.05382	7.694264139	3.500227	0.003975	0.024191
582	ENSMUSG00000029505	Ep400	137.98238	7.690704612	3.451566	0.003749	0.023794
583	ENSMUSG00000046432	Bex3	140.44029	7.688855607	3.465282	0.003825	0.023898
584	ENSMUSG00000089824	Rbm12	136.02409	7.679140078	3.447462	0.003777	0.023816
585	ENSMUSG00000040812	Agbl2	137.26082	7.677980633	3.454616	0.003818	0.023898
586	ENSMUSG00000034484	Snx2	142.82197	7.677926465	3.483166	0.00396	0.024191
587	ENSMUSG00000014856	Tmem208	140.83562	7.677686179	3.473261	0.003912	0.024156
588	ENSMUSG00000040842	Szrd1	135.42473	7.675436269	3.446267	0.003787	0.023816
589	ENSMUSG00000041958	Pigs	356.22987	7.672540309	3.229854	0.002772	0.020874
590	ENSMUSG00000026696	Vamp4	147.59186	7.664715343	2.994716	0.001821	0.018718
591	ENSMUSG00000022701	Ccdc191	134.26725	7.658220723	3.449338	0.003876	0.024153
592	ENSMUSG00000086049	NA	134.207	7.653648366	3.451476	0.003906	0.024156
593	ENSMUSG00000042275	Pelo	133.12098	7.651109599	3.446986	0.003894	0.024156
594	ENSMUSG00000076523	Igkv15-103	133.71517	7.650500358	3.450521	0.003915	0.024156
595	ENSMUSG00000041202	Pla2g2d	132.70665	7.645828719	3.447583	0.00392	0.024156
596	ENSMUSG00000016496	Cd274	143.52281	7.643458165	3.504867	0.004225	0.024819
597	ENSMUSG00000096472	Cdkn2d	135.1622	7.641365011	3.463159	0.004019	0.02429
598	ENSMUSG00000001767	Crnkl1	133.25378	7.638929722	3.454251	0.003984	0.024191
599	ENSMUSG00000097048	1600020E0	130.80213	7.637907032	3.441427	0.003923	0.024156
600	ENSMUSG00000033684	Qsox1	130.85006	7.636026056	3.442708	0.003938	0.02416
601	ENSMUSG00000032116	Stt3a	130.81401	7.635290738	3.442904	0.003942	0.02416
602	ENSMUSG00000041560	Nop53	131.23891	7.634295296	3.445781	0.003961	0.024191
603	ENSMUSG00000019054	Fis1	131.4356	7.632245792	3.447964	0.003982	0.024191
604	ENSMUSG00000075703	Selenoi	139.41235	7.63037097	3.491128	0.004214	0.024815
605	ENSMUSG00000033767	Tmem131l	141.4222	7.627932249	3.502589	0.004285	0.024997

606	ENSMUSG00000026721	Rabgap1l	133.58496	7.624770772	3.463628	0.004096	0.024526
607	ENSMUSG00000037913	Tmem156	135.69632	7.62340864	3.475573	0.004164	0.024709
608	ENSMUSG00000021982	Cdadc1	130.62467	7.622085703	3.448976	0.004032	0.024327
609	ENSMUSG00000031584	Gsr	138.76959	7.617860185	3.494459	0.004289	0.024997
610	ENSMUSG00000032897	Nfyc	133.1933	7.616126535	3.466149	0.004148	0.02465
611	ENSMUSG00000021709	Erbin	139.44405	7.610163806	3.501965	0.004365	0.025214
612	ENSMUSG00000025995	Wdr75	131.12881	7.608793042	3.458882	0.004143	0.02465
613	ENSMUSG00000004709	Cd244a	132.51427	7.608583669	3.466524	0.004185	0.024709
614	ENSMUSG00000047731	Wbp1l	137.03258	7.607271033	3.491119	0.004321	0.02507
615	ENSMUSG00000002897	Il17ra	130.78636	7.601972544	3.46067	0.004184	0.024709
616	ENSMUSG00000053317	Sec61b	126.45245	7.598917436	3.438109	0.004079	0.024526
617	ENSMUSG00000035614	Togaram1	126.47122	7.59501639	3.440327	0.004108	0.02456
618	ENSMUSG00000049760	Micos13	125.78594	7.594220525	3.436844	0.004093	0.024526
619	ENSMUSG00000066306	Numa1	125.41976	7.592521508	3.435663	0.004095	0.024526
620	ENSMUSG00000057375	Yipf1	126.44166	7.592279553	3.441634	0.004127	0.024601
621	ENSMUSG00000031667	Aktip	243.88282	7.585905628	3.046229	0.002232	0.019646
622	ENSMUSG00000068391	Chrac1	127.44021	7.58232877	3.452648	0.004232	0.024819
623	ENSMUSG00000021665	Hexb	134.09598	7.579674388	3.490345	0.004449	0.025543
624	ENSMUSG00000033931	Rbm34	126.46261	7.578163971	3.449364	0.004234	0.024819
625	ENSMUSG00000001348	Acp5	126.53969	7.574175881	3.451946	0.004266	0.024972
626	ENSMUSG00000002064	Sdf2	123.32544	7.571097853	3.435137	0.00419	0.024709
627	ENSMUSG00000028608	Czib	182.04843	7.570299605	3.235045	0.003155	0.022202
628	ENSMUSG00000038543	BC028528	88.595376	7.563276081	3.192938	0.002975	0.021532
629	ENSMUSG00000076881	Traj50	125.34957	7.555450273	3.455226	0.004373	0.025218
630	ENSMUSG00000067847	Romo1	2025.3272	7.550549207	2.675551	0.000981	0.016863
631	ENSMUSG00000027472	Pdrg1	117.611	7.54829061	2.879216	0.001656	0.018149
632	ENSMUSG00000060548	Tnfrsf19	121.14779	7.54798956	3.434841	0.004296	0.024997
633	ENSMUSG00000033845	Mrpl15	120.40378	7.543992897	3.432577	0.004302	0.024997
634	ENSMUSG00000073155	1810058I2	119.78628	7.542967259	3.429436	0.00429	0.024997
635	ENSMUSG00000029599	Ddx54	124.74309	7.539473819	3.460366	0.004478	0.025595
636	ENSMUSG00000029625	Cpsf4	123.19287	7.535970498	3.453323	0.004456	0.025549
637	ENSMUSG00000019977	Hbs1l	124.50192	7.529042359	3.464575	0.004553	0.025878
638	ENSMUSG00000049866	Arl4c	119.64509	7.52417538	3.438771	0.004432	0.025522
639	ENSMUSG00000017999	Ddx27	190.78076	7.517480161	3.285885	0.003624	0.023538
640	ENSMUSG00000040048	Ndufb10	504.60477	7.516218274	3.244817	0.003413	0.023104
641	ENSMUSG00000039826	Trub2	126.17995	7.515959702	3.481056	0.004712	0.02625
642	ENSMUSG00000021987	Mtmr6	121.35228	7.513426052	3.454693	0.004575	0.025963
643	ENSMUSG00000015289	Lage3	127.35755	7.512046071	3.489691	0.004782	0.02636
644	ENSMUSG00000064127	Med14	115.83365	7.505788274	3.425514	0.004446	0.025543
645	ENSMUSG00000097390	NA	117.4439	7.505288472	3.435689	0.004507	0.025689
646	ENSMUSG00000003438	Timm50	115.65487	7.501201653	3.4269	0.004477	0.025595
647	ENSMUSG00000022973	Synj1	115.75549	7.500770395	3.427759	0.004484	0.025595
648	ENSMUSG00000051682	Treml4	125.17274	7.500528418	3.483589	0.004806	0.02636
649	ENSMUSG00000009090	Ap1b1	120.56357	7.50033922	3.457079	0.004654	0.026223
650	ENSMUSG00000089911	Mfsd14a	172.58992	7.498993233	3.236201	0.003437	0.023124
651	ENSMUSG00000038024	Dennd4c	121.12385	7.497493073	3.461903	0.004696	0.026243
652	ENSMUSG00000024456	Diaph1	116.28575	7.496120559	3.433562	0.00454	0.025841

653	ENSMUSG00000002297	Dbf4	118.22482	7.489075356	3.44919	0.004665	0.026223
654	ENSMUSG00000059554	Ccdc28a	118.13935	7.486714782	3.449939	0.004682	0.02623
655	ENSMUSG00000031245	Hmgn5	119.89379	7.486041611	3.460796	0.004748	0.026301
656	ENSMUSG00000043140	Tmem186	116.84323	7.485515586	3.442723	0.004646	0.026215
657	ENSMUSG00000042747	Krtcap2	173.84102	7.482468806	3.236396	0.003505	0.023375
658	ENSMUSG00000020015	Cdk17	121.40129	7.480111767	3.47282	0.00485	0.026439
659	ENSMUSG00000000751	Rpa1	121.76121	7.476788138	3.476685	0.00489	0.026535
660	ENSMUSG00000063511	Snrnp70	172.0397	7.475570802	3.232527	0.003512	0.023375
661	ENSMUSG00000021870	Slmap	117.90204	7.464974688	3.460202	0.004854	0.026439
662	ENSMUSG00000014226	Cacybp	112.08506	7.461833117	3.42582	0.004667	0.026223
663	ENSMUSG00000051518	Rps19bp1	112.39424	7.459268416	3.429187	0.0047	0.026243
664	ENSMUSG00000056851	Pcbp2	284.92473	7.452059906	2.857245	0.001794	0.018718
665	ENSMUSG00000061950	Ppp4r1	118.9082	7.448236507	3.475158	0.005033	0.026813
666	ENSMUSG00000078763	Slfn1	110.09027	7.446149759	3.421497	0.004722	0.026267
667	ENSMUSG00000069633	Pex11g	114.3646	7.444864876	3.449382	0.004896	0.026535
668	ENSMUSG00000056629	Fkbp2	109.49739	7.442036074	3.419869	0.004734	0.026293
669	ENSMUSG00000031232	Magt1	110.13411	7.441006193	3.424581	0.004767	0.026354
670	ENSMUSG00000039395	Mreg	164.48688	7.439629441	3.234519	0.003673	0.023656
671	ENSMUSG00000028524	Sgip1	108.68515	7.437152424	3.417196	0.004743	0.026301
672	ENSMUSG00000058407	Txndc9	108.02425	7.430667451	3.416364	0.004772	0.026354
673	ENSMUSG00000024357	Sil1	111.21428	7.430463489	3.437282	0.004899	0.026535
674	ENSMUSG00000021578	Ccdc127	115.33082	7.427644387	3.464589	0.005081	0.026813
675	ENSMUSG00000040990	Sh3kbp1	1383.6119	7.426724639	3.401835	0.004704	0.026243
676	ENSMUSG00000028060	Khdc4	107.59179	7.424900202	3.416639	0.004803	0.02636
677	ENSMUSG00000030726	Pold3	107.77424	7.423734942	3.418487	0.004821	0.026402
678	ENSMUSG00000076609	Igkc	7669.1225	7.420636488	3.073871	0.002886	0.021206
679	ENSMUSG00000072109	A530040E1	159.71208	7.419591841	3.223912	0.003699	0.023748
680	ENSMUSG00000038683	Pak1ip1	107.20856	7.415123266	3.419419	0.004872	0.026497
681	ENSMUSG00000039879	Heca	109.66279	7.414513663	3.435909	0.004976	0.026766
682	ENSMUSG00000078348	Sf3b5	159.12546	7.410250123	3.226635	0.003755	0.023794
683	ENSMUSG00000028044	Cks1b	106.76399	7.407301394	3.420718	0.004921	0.026582
684	ENSMUSG00000002635	Pdcd2l	108.43382	7.405229401	3.432923	0.005008	0.026813
685	ENSMUSG00000038179	Slamf7	111.17434	7.4047527	3.450915	0.005122	0.026922
686	ENSMUSG00000022338	Eny2	104.93116	7.39872678	3.413005	0.00492	0.026582
687	ENSMUSG00000028003	Lrat	108.65772	7.396896886	3.438908	0.00509	0.026829
688	ENSMUSG00000038774	Ascc3	111.07894	7.395697007	3.455174	0.005199	0.027217
689	ENSMUSG00000048351	Coa7	106.3093	7.393691974	3.425081	0.005022	0.026813
690	ENSMUSG00000010067	Rassf1	118.4194	7.393531042	2.987513	0.002544	0.020412
691	ENSMUSG00000020634	Ubxn2a	442.91537	7.39247718	3.218983	0.003789	0.023816
692	ENSMUSG00000068522	Aard	105.79391	7.39048755	3.423364	0.005028	0.026813
693	ENSMUSG00000060679	Mrps9	103.6927	7.388355139	3.410179	0.004958	0.026705
694	ENSMUSG00000046567	4930430F0	106.23136	7.386741406	3.42834	0.00508	0.026813
695	ENSMUSG00000021474	Sfxn1	160.44252	7.386656617	3.24678	0.003976	0.024191
696	ENSMUSG00000010392	Gosr1	104.49479	7.378530554	3.421064	0.005079	0.026813
697	ENSMUSG00000034998	Foxn2	102.6937	7.376718912	3.40962	0.005017	0.026813
698	ENSMUSG00000086083	NA	103.77863	7.376602219	3.417205	0.005066	0.026813
699	ENSMUSG00000098197	BC051537	103.46405	7.374045856	3.416433	0.005075	0.026813

700	ENSMUSG00000033454	Zbtb1	273.26705	7.371003626	2.604504	0.001043	0.016863
701	ENSMUSG00000015217	Hmgb3	107.09022	7.369554743	3.443387	0.005271	0.027351
702	ENSMUSG00000024122	Pdpk1	583.70902	7.367710198	2.936944	0.002378	0.020028
703	ENSMUSG00000085590	NA	101.42351	7.364862828	3.40721	0.005067	0.026813
704	ENSMUSG00000023025	Larp4	164.61705	7.364409429	3.263805	0.004181	0.024709
705	ENSMUSG00000028890	Mtf1	105.36231	7.363740383	3.434996	0.00525	0.027351
706	ENSMUSG00000028811	Yars	151.55509	7.359156656	3.219773	0.003942	0.02416
707	ENSMUSG00000027285	Haus2	100.73994	7.357615335	3.406345	0.005102	0.026852
708	ENSMUSG00000020775	Mrpl38	102.61771	7.356195228	3.420315	0.005199	0.027217
709	ENSMUSG00000056763	Cspp1	89.451551	7.352938418	2.773354	0.001687	0.018377
710	ENSMUSG00000089844	A530032D1	154.28709	7.352069415	3.222579	0.003991	0.024196
711	ENSMUSG00000037337	Map4k1	101.34665	7.348462097	3.41565	0.005212	0.027252
712	ENSMUSG00000028684	Urod	99.162519	7.340858344	3.404249	0.005182	0.0272
713	ENSMUSG00000009863	Sdhb	424.01816	7.340282036	3.216725	0.004011	0.024274
714	ENSMUSG00000039473	Ubn1	288.45044	7.336377556	3.266204	0.004331	0.02509
715	ENSMUSG00000024006	Stk38	102.69811	7.335057254	2.906633	0.002331	0.01987
716	ENSMUSG00000028458	Tesk1	98.718175	7.331228623	3.406328	0.005249	0.027351
717	ENSMUSG00000028842	Ago3	105.0538	7.328094279	3.452181	0.005569	0.028289
718	ENSMUSG00000024454	Hdac3	98.051884	7.327180018	3.403709	0.005255	0.027351
719	ENSMUSG00000000766	Oprm1	104.3741	7.32507067	3.449243	0.005567	0.028289
720	ENSMUSG00000069919	Hba-a1	99.285577	7.318037528	3.417645	0.005399	0.027932
721	ENSMUSG00000061589	Dot1l	103.06591	7.313363986	3.446664	0.00562	0.028368
722	ENSMUSG00000096221	NA	143.78908	7.30797232	3.21014	0.004122	0.024601
723	ENSMUSG00000027778	Ift80	103.35782	7.306216947	3.45251	0.005702	0.028572
724	ENSMUSG00000086090	NA	96.220549	7.303633197	3.403178	0.005387	0.027905
725	ENSMUSG00000047146	Tet1	97.110735	7.303056576	3.410068	0.005437	0.028086
726	ENSMUSG00000029530	Ccr9	97.873105	7.294808108	3.420147	0.005553	0.028289
727	ENSMUSG00000034880	Mrpl34	93.64412	7.279176698	3.397249	0.005491	0.028235
728	ENSMUSG00000006014	Prg4	96.533179	7.277926767	3.419579	0.00565	0.028477
729	ENSMUSG00000008683	Rps15a	1057.3345	7.275217151	2.570706	0.001089	0.016863
730	ENSMUSG00000050379	Septin6	104.69391	7.2736407	2.963779	0.002826	0.021022
731	ENSMUSG00000055065	Ddx17	92.955158	7.272138942	3.395852	0.005523	0.028286
732	ENSMUSG00000057409	Zfp53	92.850873	7.270472725	3.395969	0.005534	0.028289
733	ENSMUSG00000085741	5430405H(	172.11135	7.270381497	2.894422	0.002471	0.02039
734	ENSMUSG00000028329	Xpa	100.19997	7.269014368	3.450731	0.005919	0.028999
735	ENSMUSG00000090054	NA	94.618506	7.262799483	3.413628	0.005701	0.028572
736	ENSMUSG00000035042	Ccl5	94.313446	7.260418555	3.412631	0.005709	0.028572
737	ENSMUSG00000061315	Naca	92.063023	7.25994453	3.395691	0.005595	0.028345
738	ENSMUSG00000005540	Fcer2a	92.942643	7.257955209	3.403567	0.005661	0.028496
739	ENSMUSG00000027860	Vangl1	95.284797	7.251897581	3.424561	0.005845	0.028789
740	ENSMUSG00000022635	Zcrb1	91.877986	7.246980402	3.401404	0.005713	0.028572
741	ENSMUSG00000036551	Akap14	150.81574	7.246134423	3.267182	0.004801	0.02636
742	ENSMUSG00000030002	Dusp11	58.499096	7.245349015	3.061786	0.003496	0.023375
743	ENSMUSG00000090137	Uba52	95.399011	7.244582886	3.429392	0.005924	0.028999
744	ENSMUSG00000038398	Upf3a	91.285103	7.243174272	3.398887	0.005719	0.028572
745	ENSMUSG00000024948	Map4k2	92.463113	7.239774246	3.409874	0.005817	0.028763
746	ENSMUSG00000030272	Camk1	91.021262	7.235473314	3.401068	0.005782	0.028661

747	ENSMUSG00000027828	Ssr3	142.67255	7.23420538	3.232613	0.004634	0.026183
748	ENSMUSG00000001280	Sp1	89.995277	7.23372503	3.393946	0.005743	0.028611
749	ENSMUSG00000059456	Ptk2b	97.875503	7.232506461	3.45394	0.006175	0.029806
750	ENSMUSG00000017418	Arl5b	89.639892	7.230033476	3.393162	0.00576	0.028625
751	ENSMUSG00000000339	Rtca	89.213685	7.228177245	3.390788	0.005755	0.028625
752	ENSMUSG00000021108	Prkch	143.56477	7.224908884	3.258057	0.004854	0.026439
753	ENSMUSG00000043702	Pde12	58.38256	7.223090242	3.074272	0.003668	0.023656
754	ENSMUSG00000033688	Inhca	88.69702	7.220842125	3.390697	0.0058	0.028712
755	ENSMUSG00000021236	Entpd5	89.324452	7.220525529	3.395899	0.005839	0.028789
756	ENSMUSG00000031931	Ankrd49	91.204801	7.216904274	3.412694	0.005981	0.029205
757	ENSMUSG00000034931	Dhx8	87.882827	7.212216784	3.388889	0.005841	0.028789
758	ENSMUSG00000086507	NA	88.125721	7.210893292	3.391592	0.005868	0.028833
759	ENSMUSG00000038914	Dido1	94.483344	7.210673187	3.441008	0.006225	0.029882
760	ENSMUSG00000021645	Smn1	131.70729	7.206572956	3.203895	0.004586	0.025988
761	ENSMUSG00000037013	Ss18	90.539784	7.201935214	3.41572	0.006099	0.029732
762	ENSMUSG00000019802	Sec63	87.798402	7.201262931	3.394264	0.005948	0.029081
763	ENSMUSG00000050088	1600012H(	87.296202	7.200827674	3.390416	0.005923	0.028999
764	ENSMUSG00000048668	Rhno1	89.718921	7.196399096	3.412299	0.00611	0.029732
765	ENSMUSG00000030271	Ogg1	89.391152	7.18894613	3.413803	0.00617	0.029806
766	ENSMUSG00000000708	Kat2b	59.358395	7.188521992	3.109166	0.004047	0.024384
767	ENSMUSG00000040354	Mars1	92.967262	7.186369251	3.442809	0.0064	0.030342
768	ENSMUSG00000048327	Ckap2l	92.274628	7.185491102	3.438042	0.006371	0.030314
769	ENSMUSG00000031575	Ash2l	491.57622	7.185252482	3.408624	0.006156	0.029806
770	ENSMUSG00000037531	Mrpl47	88.424748	7.180389015	3.410805	0.006204	0.029882
771	ENSMUSG00000029254	Stap1	88.520826	7.17106498	3.416682	0.006309	0.030094
772	ENSMUSG00000093880	NA	87.152833	7.170661576	3.40589	0.006232	0.029882
773	ENSMUSG00000032892	Rangrf	84.884949	7.162842405	3.39148	0.006177	0.029806
774	ENSMUSG00000037470	Uggt1	84.251896	7.161218363	3.387055	0.006154	0.029806
775	ENSMUSG00000028759	Hp1bp3	129.8979	7.160518298	3.205996	0.004846	0.026439
776	ENSMUSG00000020079	Supv3l1	83.777762	7.155994541	3.385931	0.00618	0.029806
777	ENSMUSG00000026469	Xpr1	90.620124	7.154711055	3.442044	0.006611	0.030953
778	ENSMUSG00000037148	Arhgap10	86.870856	7.150321251	3.414763	0.006435	0.030433
779	ENSMUSG00000036199	Ndufa13	82.83058	7.145343829	3.383737	0.006234	0.029882
780	ENSMUSG00000024491	Rbm27	233.68168	7.142753413	3.221905	0.005058	0.026813
781	ENSMUSG00000048271	Rbm33	90.127853	7.142541341	3.444822	0.006717	0.031165
782	ENSMUSG00000027030	Stk39	82.428767	7.137947386	3.384384	0.006289	0.030069
783	ENSMUSG00000055204	Ankrd17	81.58025	7.131083563	3.380825	0.006308	0.030094
784	ENSMUSG00000023027	Atf1	124.64766	7.129209928	3.193456	0.00493	0.026595
785	ENSMUSG00000002728	Naa20	318.3231	7.128781823	3.204113	0.00501	0.026813
786	ENSMUSG00000022774	Ncbp2	87.099286	7.12828595	3.428647	0.006693	0.031128
787	ENSMUSG00000036792	Mbd5	86.93973	7.127941583	3.427548	0.006687	0.031128
788	ENSMUSG00000041203	Trir	84.906308	7.127924155	3.410907	0.006559	0.030835
789	ENSMUSG00000010051	Hyal1	81.832212	7.126014971	3.385837	0.00638	0.03032
790	ENSMUSG00000033306	Lpp	82.776808	7.123735458	3.39525	0.006468	0.030552
791	ENSMUSG00000042831	Alkbh6	86.451993	7.123705952	3.425909	0.006704	0.031143
792	ENSMUSG00000033209	Ttc28	81.531236	7.12351134	3.384605	0.006388	0.03032
793	ENSMUSG00000035125	Gcfc2	85.184613	7.121039497	3.416987	0.006654	0.031057

794	ENSMUSG00000027133	Nop10	82.199701	7.117607899	3.393669	0.006498	0.03062
795	ENSMUSG00000027455	Nsfl1c	87.593955	7.116061051	3.439243	0.006862	0.03165
796	ENSMUSG00000030168	Adipor2	81.443589	7.114433274	3.388869	0.006483	0.030585
797	ENSMUSG00000027242	Wdr76	86.260698	7.111858305	3.43081	0.006826	0.031523
798	ENSMUSG00000045252	Zfp574	82.216526	7.109679878	3.398202	0.006588	0.030932
799	ENSMUSG00000030007	Cct7	369.07793	7.10756332	3.248536	0.005459	0.028168
800	ENSMUSG00000042507	Mideas	84.346026	7.10653831	3.417965	0.006764	0.031272
801	ENSMUSG00000055932	Fto	157.23818	7.106031146	2.995984	0.003687	0.02371
802	ENSMUSG00000058706	0610030E2	83.71687	7.105482808	3.413276	0.006735	0.03121
803	ENSMUSG00000044783	A730008H1	125.39339	7.074465933	3.230632	0.005526	0.028286
804	ENSMUSG00000031168	Ebp	82.312192	7.074243941	3.418484	0.007001	0.032254
805	ENSMUSG00000028822	Tmem50a	301.10203	7.072431953	3.195711	0.005273	0.027351
806	ENSMUSG00000020850	Prpf8	54.299029	7.058187888	3.120549	0.004794	0.02636
807	ENSMUSG00000062376	Borcs7	209.21478	7.0378638	3.201055	0.005523	0.028286
808	ENSMUSG00000030869	Ndufab1	117.70645	7.03678752	3.204997	0.00556	0.028289
809	ENSMUSG00000028309	Rnf20	115.6202	7.036572357	3.207069	0.005578	0.028293
810	ENSMUSG00000019769	NA	115.15287	7.031359376	3.192217	0.005494	0.028235
811	ENSMUSG00000026709	Dars2	117.15781	7.029034697	3.206658	0.005621	0.028368
812	ENSMUSG00000019738	Polr2i	113.39517	7.025386554	3.184144	0.005468	0.028173
813	ENSMUSG00000023050	Map3k12	545.76871	7.009183068	2.938155	0.003736	0.023794
814	ENSMUSG00000030315	Vgll4	108.56949	6.999217356	3.181876	0.005612	0.028368
815	ENSMUSG00000058013	Septin11	667.11473	6.993018481	3.391105	0.007386	0.033908
816	ENSMUSG00000019897	Ccdc59	108.79697	6.979279074	3.1806	0.005728	0.028573
817	ENSMUSG00000040557	Mettl27	107.41062	6.93885964	3.195329	0.006115	0.029732
818	ENSMUSG00000032409	Atr	80.214843	6.926432106	3.480448	0.00872	0.03907
819	ENSMUSG00000087260	Lamtor5	80.052701	6.924110233	3.480303	0.008739	0.039074
820	ENSMUSG00000039968	Rsbn1l	189.7854	6.923128646	3.196487	0.006232	0.029882
821	ENSMUSG00000091133	NA	79.767052	6.920019179	3.480049	0.008773	0.039129
822	ENSMUSG00000039849	Pcif1	79.594755	6.91754535	3.479895	0.008794	0.039176
823	ENSMUSG00000093006	NA	79.379928	6.914449358	3.479703	0.008819	0.039237
824	ENSMUSG00000028832	Stmn1	79.248663	6.912548314	3.479585	0.008835	0.039237
825	ENSMUSG00000028826	Maco1	79.231163	6.912304731	3.47957	0.008837	0.039237
826	ENSMUSG00000028494	Plin2	102.09194	6.911015317	3.17371	0.00612	0.029732
827	ENSMUSG00000035310	NA	79.049387	6.909660375	3.479406	0.008859	0.03929
828	ENSMUSG00000024414	Mrpl27	78.7797	6.905763501	3.479165	0.008892	0.039349
829	ENSMUSG00000052033	Pfdn4	78.735221	6.90511937	3.479125	0.008898	0.039349
830	ENSMUSG00000041881	Ndufa7	78.692915	6.90449355	3.479086	0.008903	0.039349
831	ENSMUSG00000049044	Rapgef4	78.469656	6.901250392	3.478885	0.00893	0.039426
832	ENSMUSG00000021374	Nup153	78.306878	6.898860252	3.478738	0.00895	0.03947
833	ENSMUSG00000048191	NA	78.191576	6.897179957	3.478634	0.008965	0.039489
834	ENSMUSG00000005886	Ncoa2	106.62816	6.893474012	3.217084	0.00662	0.030953
835	ENSMUSG00000003402	Prkcsh	103.30387	6.891889829	3.193625	0.006425	0.030422
836	ENSMUSG00000038127	Ccdc50	77.52225	6.887354841	3.478028	0.009048	0.039707
837	ENSMUSG00000080058	NA	77.489649	6.886872403	3.477998	0.009052	0.039707
838	ENSMUSG00000040209	Zfp704	77.471513	6.88660486	3.477982	0.009055	0.039707
839	ENSMUSG00000029390	Tmed2	76.827031	6.877048572	3.477394	0.009137	0.039916
840	ENSMUSG00000028433	Ubap2	76.621272	6.873981245	3.477205	0.009163	0.039916

841	ENSMUSG00000020611	Gna13	96.872661	6.872848249	3.172024	0.006368	0.030314
842	ENSMUSG00000025869	Nop16	76.543105	6.872821612	3.477134	0.009173	0.039916
843	ENSMUSG00000001127	Araf	76.539882	6.872758982	3.47713	0.009174	0.039916
844	ENSMUSG00000018378	2210416O1	76.417236	6.870935003	3.477019	0.00919	0.039941
845	ENSMUSG00000076532	Igkv4-91	76.204132	6.867745047	3.476823	0.009217	0.039972
846	ENSMUSG00000040189	Odad1	100.28396	6.865296584	3.187293	0.006558	0.030835
847	ENSMUSG00000015749	Anp32e	75.846162	6.862359044	3.476493	0.009264	0.040087
848	ENSMUSG00000013698	Pea15a	75.759377	6.861041686	3.476412	0.009276	0.040093
849	ENSMUSG00000010554	Mettl16	75.524239	6.857501949	3.476196	0.009307	0.040182
850	ENSMUSG00000011179	Odc1	75.410024	6.855772267	3.47609	0.009322	0.040203
851	ENSMUSG00000026177	Slc11a1	75.12741	6.85147388	3.475828	0.00936	0.040322
852	ENSMUSG00000026011	Ctla4	74.56087	6.842825429	3.475301	0.009436	0.040529
853	ENSMUSG00000039936	Pik3cd	74.490685	6.841764193	3.475236	0.009446	0.040529
854	ENSMUSG00000030888	Rrp8	74.46629	6.841387002	3.475213	0.009449	0.040529
855	ENSMUSG00000028517	Plpp3	74.318388	6.839121726	3.475075	0.009469	0.040571
856	ENSMUSG00000028519	Dab1	43.602065	6.837047485	3.087662	0.005867	0.028833
857	ENSMUSG00000048376	F2r	74.187537	6.837026759	3.474948	0.009488	0.040607
858	ENSMUSG00000018923	Med11	174.41188	6.836777436	3.184896	0.006744	0.031218
859	ENSMUSG00000022031	Elp3	73.744953	6.830261996	3.474537	0.009549	0.04072
860	ENSMUSG00000095351	Igkv3-2	73.691181	6.829440375	3.474487	0.009556	0.04072
861	ENSMUSG00000097546	NA	73.183809	6.821542918	3.474008	0.009627	0.040937
862	ENSMUSG00000035258	Abi3bp	73.179275	6.821472258	3.474004	0.009628	0.040937
863	ENSMUSG00000015536	Mocs2	72.945861	6.817843512	3.473784	0.009661	0.041033
864	ENSMUSG00000001741	Il16	72.829286	6.816003869	3.473673	0.009678	0.041059
865	ENSMUSG00000097379	NA	181.97216	6.80818775	2.8885	0.004355	0.025192
866	ENSMUSG00000025533	Asl	72.231007	6.806591457	3.473105	0.009764	0.041334
867	ENSMUSG00000029923	Rab19	72.081381	6.804228031	3.472962	0.009785	0.041377
868	ENSMUSG00000039428	Tmem135	71.956149	6.802249961	3.472843	0.009804	0.041377
869	ENSMUSG00000018736	Ndel1	71.943184	6.802055569	3.472831	0.009805	0.041377
870	ENSMUSG00000018239	Zcchc10	71.531028	6.795490555	3.472436	0.009866	0.041572
871	ENSMUSG00000027774	Gfm1	71.483739	6.794742679	3.472391	0.009873	0.041572
872	ENSMUSG00000019761	Krt10	71.356783	6.792717615	3.472269	0.009892	0.041607
873	ENSMUSG00000097605	NA	71.007019	6.787109059	3.471932	0.009944	0.041781
874	ENSMUSG00000020166	Cnot2	70.684235	6.781918125	3.471621	0.009992	0.04194
875	ENSMUSG00000022946	Dop1b	70.396	6.777259548	3.471342	0.010036	0.042079
876	ENSMUSG00000096422	Igkv12-44	70.235582	6.774658162	3.471186	0.010061	0.042093
877	ENSMUSG00000036333	Kidins220	69.68759	6.765753468	3.470653	0.010145	0.042363
878	ENSMUSG00000055835	Zfp1	69.6578	6.765260994	3.470624	0.01015	0.042363
879	ENSMUSG00000020706	Ftsj3	69.604252	6.764387719	3.470572	0.010158	0.042363
880	ENSMUSG00000036241	Ube2r2	67.304524	6.761695401	2.937305	0.005024	0.026813
881	ENSMUSG00000018572	Phf23	69.354239	6.760280998	3.470327	0.010197	0.042367
882	ENSMUSG00000009563	Tor2a	69.3225	6.759760108	3.470296	0.010202	0.042367
883	ENSMUSG00000038766	Gabpb2	144.99258	6.75910225	3.013787	0.00572	0.028572
884	ENSMUSG00000039414	Heatr5b	69.268952	6.758882765	3.470243	0.010211	0.042367
885	ENSMUSG00000020175	Rab36	69.232904	6.75828059	3.470208	0.010216	0.042367
886	ENSMUSG00000063882	Uqcrh	69.115654	6.756363827	3.470093	0.010235	0.042367
887	ENSMUSG00000046516	Cox17	90.019797	6.75356588	2.756247	0.003592	0.023485

888	ENSMUSG00000048285	Frmd6	68.666776	6.748951356	3.469652	0.010306	0.042572
889	ENSMUSG00000029050	Ski	88.99978	6.746821153	3.184643	0.007435	0.034094
890	ENSMUSG00000021133	Susd6	68.303409	6.742904771	3.469293	0.010364	0.042729
891	ENSMUSG00000019843	Fyn	68.239932	6.741847597	3.46923	0.010375	0.042729
892	ENSMUSG00000024759	Atl3	68.226329	6.741620992	3.469216	0.010377	0.042729
893	ENSMUSG00000027651	Rprd1b	68.009778	6.737997519	3.469001	0.010412	0.04283
894	ENSMUSG00000071644	Eef1g	86.176784	6.736955213	3.166221	0.007332	0.033701
895	ENSMUSG00000025427	Rnf165	67.581622	6.730823624	3.468576	0.010482	0.043012
896	ENSMUSG00000068921	Dap3	67.418619	6.728068275	3.468413	0.010509	0.043012
897	ENSMUSG00000049878	Rlf	67.384932	6.727506404	3.46838	0.010515	0.043012
898	ENSMUSG00000037286	Stag1	67.33959	6.726741358	3.468335	0.010522	0.043012
899	ENSMUSG00000033099	Nol12	211.85492	6.726633315	2.541396	0.002232	0.019646
900	ENSMUSG00000040111	Gramd1b	67.133194	6.723235802	3.468127	0.010557	0.043063
901	ENSMUSG00000024855	Pacs1	67.030858	6.721495883	3.468024	0.010574	0.043086
902	ENSMUSG00000034252	Senp6	66.96738	6.720419118	3.467961	0.010585	0.043086
903	ENSMUSG00000035027	Map2k2	86.670915	6.720100381	3.165937	0.007465	0.03415
904	ENSMUSG00000038013	Wipf2	66.785154	6.71732071	3.467778	0.010615	0.043149
905	ENSMUSG00000017478	Zc3h18	66.680007	6.715528298	3.467672	0.010633	0.043149
906	ENSMUSG00000051278	Zgrf1	168.8939	6.711688351	3.236196	0.00825	0.037178
907	ENSMUSG00000091271	NA	84.957692	6.710740765	3.156493	0.007446	0.034102
908	ENSMUSG00000014030	Pax5	66.264367	6.708439741	3.467254	0.010704	0.043378
909	ENSMUSG00000044709	Gemin7	66.152962	6.706522086	3.467141	0.010723	0.043379
910	ENSMUSG00000016831	Tox4	66.078693	6.705241905	3.467066	0.010736	0.043386
911	ENSMUSG00000094951	NA	65.785699	6.700199488	3.466769	0.010786	0.043444
912	ENSMUSG00000037210	Fam193a	65.763028	6.699808117	3.466746	0.01079	0.043444
913	ENSMUSG00000058818	Pirb	65.73972	6.699392493	3.466722	0.010794	0.043444
914	ENSMUSG00000031453	Rasa3	226.80136	6.699194111	3.207449	0.008062	0.036583
915	ENSMUSG00000038332	Sesn1	152.92772	6.690442167	3.174611	0.007798	0.03559
916	ENSMUSG00000003721	Insig2	65.20016	6.690035824	3.466172	0.010889	0.043735
917	ENSMUSG00000032000	Birc3	65.061326	6.68762031	3.46603	0.010914	0.043754
918	ENSMUSG00000031982	Arv1	65.004331	6.686618865	3.465972	0.010924	0.043754
919	ENSMUSG00000039834	Zfp335	64.983384	6.686257524	3.465951	0.010927	0.043754
920	ENSMUSG00000038344	Txlng	64.471478	6.677259809	3.465424	0.011019	0.044078
921	ENSMUSG00000068739	Sars	64.300044	6.674240977	3.465247	0.01105	0.044157
922	ENSMUSG00000004383	Large1	64.078734	6.670331851	3.465019	0.011091	0.044272
923	ENSMUSG00000070327	Rnf213	80.04759	6.668449678	3.152183	0.00775	0.035414
924	ENSMUSG00000020078	Vps26a	63.747106	6.664432339	3.464675	0.011152	0.04438
925	ENSMUSG00000058318	Phf21a	82.139231	6.663379164	3.159972	0.007875	0.035815
926	ENSMUSG00000021666	Gfm2	364.43496	6.663329451	2.825832	0.00462	0.026142
927	ENSMUSG00000023845	Lnpep	83.408789	6.662998888	3.171076	0.007995	0.036319
928	ENSMUSG00000020170	Frs2	63.591222	6.66165111	3.464512	0.011181	0.04445
929	ENSMUSG00000073468	Sft2d1	79.489032	6.658626164	3.152917	0.007841	0.035703
930	ENSMUSG00000027677	Ttc14	63.383514	6.657943133	3.464296	0.011219	0.044559
931	ENSMUSG00000097099	Gm9917	63.290883	6.656291834	3.4642	0.011237	0.044582
932	ENSMUSG00000032555	Topbp1	63.222235	6.655047215	3.464128	0.01125	0.044588
933	ENSMUSG00000024241	Sos1	62.947602	6.650104512	3.46384	0.011301	0.044703
934	ENSMUSG00000008668	Rps18	83.545857	6.649970069	3.181053	0.008214	0.037117

935	ENSMUSG00000020064	Herc4	62.551185	6.642950712	3.463425	0.011377	0.044865
936	ENSMUSG00000055553	Kxd1	207.52592	6.64158658	3.175413	0.008227	0.037117
937	ENSMUSG00000024683	Mrpl16	62.346512	6.639221981	3.463208	0.011416	0.044976
938	ENSMUSG00000024548	Setbp1	62.088929	6.634533114	3.462936	0.011466	0.045127
939	ENSMUSG00000038619	Ensa	61.705478	6.627507461	3.46253	0.011541	0.045376
940	ENSMUSG00000019961	Tmpo	78.606964	6.622742898	3.151579	0.008136	0.036876
941	ENSMUSG00000037149	Ddx1	61.022549	6.614908264	3.461802	0.011677	0.045818
942	ENSMUSG00000020783	Ncbp3	79.535596	6.609344297	3.168749	0.008443	0.037914
943	ENSMUSG00000030138	Bms1	60.66263	6.608205899	3.461415	0.01175	0.046024
944	ENSMUSG00000039457	Ppl	60.646442	6.607896118	3.461398	0.011753	0.046024
945	ENSMUSG00000029464	Gpn3	60.504161	6.605232201	3.461244	0.011782	0.046067
946	ENSMUSG00000028126	Pip5k1a	60.478043	6.604733207	3.461215	0.011787	0.046067
947	ENSMUSG00000037339	Fam53a	60.286748	6.601144871	3.461009	0.011827	0.046102
948	ENSMUSG00000035469	Rcbtb1	60.262991	6.60070913	3.460984	0.011831	0.046102
949	ENSMUSG00000066442	Mthfs	75.158347	6.600155412	3.14634	0.008279	0.037264
950	ENSMUSG00000023106	Denr	75.396707	6.597792364	3.150235	0.008343	0.037509
951	ENSMUSG00000034422	Parp14	60.087247	6.597393974	3.460793	0.011868	0.046197
952	ENSMUSG00000044820	AY074887	80.571966	6.595006671	3.18649	0.008771	0.039129
953	ENSMUSG00000060149	BC002059	59.717172	6.590408576	3.460392	0.011945	0.046451
954	ENSMUSG00000034636	Zyg11b	79.105355	6.575410795	3.200297	0.009112	0.039913
955	ENSMUSG00000042272	Sestd1	58.51608	6.567403774	3.459074	0.012202	0.047169
956	ENSMUSG00000097346	Rps18-ps4	58.338163	6.563972553	3.458879	0.01224	0.047225
957	ENSMUSG00000032959	Pebp1	57.953212	6.556462314	3.45845	0.012326	0.047507
958	ENSMUSG00000021557	Agtpbp1	78.035252	6.556240174	3.186869	0.009143	0.039916
959	ENSMUSG00000030104	Edem1	729.62275	6.55279207	3.351784	0.011108	0.044297
960	ENSMUSG00000097114	NA	57.612067	6.549802414	3.458071	0.012402	0.047753
961	ENSMUSG00000021907	Msmb	57.330951	6.54427665	3.457756	0.012465	0.04795
962	ENSMUSG00000046836	Brox	71.347138	6.543748476	3.141601	0.008741	0.039074
963	ENSMUSG00000026812	Tsc1	57.246114	6.542578191	3.45766	0.012485	0.047978
964	ENSMUSG00000037519	Ppfia1	56.965636	6.537048128	3.457345	0.012549	0.048129
965	ENSMUSG00000026577	Blzf1	56.804581	6.533827719	3.457163	0.012586	0.048166
966	ENSMUSG00000070780	Rbm47	214.7505	6.533431981	3.188899	0.00939	0.040407
967	ENSMUSG00000058586	Serhl	30.210181	6.533411063	2.991061	0.007136	0.032837
968	ENSMUSG00000001062	Vps9d1	56.764411	6.533046352	3.457118	0.012595	0.048166
969	ENSMUSG00000045095	Magi1	56.706329	6.531893329	3.457053	0.012608	0.04817
970	ENSMUSG00000052085	Dock8	56.51807	6.528115309	3.456839	0.012652	0.048241
971	ENSMUSG00000036315	Polr1h	56.414422	6.526060663	3.456722	0.012676	0.048241
972	ENSMUSG00000020400	Tnip1	55.956925	6.516840028	3.4562	0.012784	0.048441
973	ENSMUSG00000025958	Creb1	55.910722	6.515904809	3.456147	0.012795	0.048441
974	ENSMUSG00000074899	Sptbn5	55.784629	6.513360293	3.456003	0.012825	0.048441
975	ENSMUSG00000041733	Coq5	55.729133	6.512248735	3.45594	0.012839	0.048441
976	ENSMUSG00000098066	NA	55.64838	6.510615564	3.455848	0.012858	0.048441
977	ENSMUSG00000001785	Pwp1	55.637813	6.51038011	3.455835	0.012861	0.048441
978	ENSMUSG00000026972	Arrdc1	467.79593	6.509941668	3.15678	0.009242	0.040036
979	ENSMUSG00000025461	Scart1	55.555337	6.508703297	3.45574	0.012881	0.048441
980	ENSMUSG00000041303	Gtf3c3	55.530493	6.508222933	3.455713	0.012886	0.048441
981	ENSMUSG00000043940	Wdfy3	55.529631	6.508202429	3.455712	0.012886	0.048441

982	ENSMUSG00000022807	Osbpl11	55.426433	6.506091508	3.455593	0.012911	0.048441
983	ENSMUSG00000038803	Ost4	55.336613	6.504264894	3.455489	0.012933	0.048441
984	ENSMUSG00000025786	Zdhhc3	55.159782	6.500654783	3.455286	0.012976	0.048488
985	ENSMUSG00000028343	Erp44	54.928542	6.495916323	3.455019	0.013033	0.048534
986	ENSMUSG00000050229	Pigm	54.903511	6.495377532	3.454988	0.013039	0.048534
987	ENSMUSG00000095285	Ighv5-9	54.737247	6.491979034	3.454797	0.01308	0.048534
988	ENSMUSG00000029191	Rfc1	54.710042	6.491418473	3.454765	0.013087	0.048534
989	ENSMUSG00000053063	Clec12a	54.685648	6.490909382	3.454737	0.013093	0.048534
990	ENSMUSG00000033439	Trmt13	54.675718	6.490701421	3.454725	0.013096	0.048534
991	ENSMUSG00000009772	Nuak2	54.476442	6.486568721	3.454492	0.013145	0.048646
992	ENSMUSG00000021763	Cspg4b	54.436271	6.485756001	3.454447	0.013155	0.048646
993	ENSMUSG00000092397	C130080G1	54.324867	6.483437006	3.454316	0.013183	0.048703
994	ENSMUSG00000074024	4632427E1	54.133797	6.479446171	3.454092	0.013232	0.048746
995	ENSMUSG00000019846	Lama4	54.130762	6.479405714	3.45409	0.013232	0.048746
996	ENSMUSG00000031537	Ikbkb	68.821221	6.478285405	3.154816	0.009533	0.040712
997	ENSMUSG00000010205	Raver1	54.076352	6.478271806	3.454026	0.013246	0.048751
998	ENSMUSG00000026107	Nabp1	298.9902	6.477557955	3.170721	0.009735	0.041257
999	ENSMUSG00000022770	Dlg1	173.17452	6.472359294	2.543659	0.00319	0.022339
1000	ENSMUSG00000042548	Asxl1	53.705004	6.470473767	3.453588	0.013341	0.049055
1001	ENSMUSG00000026915	Strbp	129.24919	6.460478422	2.794681	0.00557	0.028289
1002	ENSMUSG00000074646	6430550D2	53.18077	6.45942866	3.452969	0.013477	0.049416
1003	ENSMUSG00000018707	Dync1h1	53.179908	6.459407099	3.452968	0.013477	0.049416
1004	ENSMUSG00000020444	Guk1	53.097432	6.457655514	3.45287	0.013499	0.049421
1005	ENSMUSG00000004455	Ppp1cc	53.078209	6.457261324	3.452848	0.013504	0.049421
1006	ENSMUSG00000085208	Brip1os	52.98213	6.455217095	3.452734	0.013529	0.049468
1007	ENSMUSG00000033685	Ucp2	304.00867	6.446384514	3.184693	0.010234	0.042367
1008	ENSMUSG00000027346	Gpcpd1	1157.3545	6.443740604	3.335301	0.012178	0.047123
1009	ENSMUSG00000063884	Ptcd3	397.55787	6.434215428	3.160645	0.010061	0.042093
1010	ENSMUSG00000032301	Psma4	396.36431	6.425725262	3.163634	0.010189	0.042367
1011	ENSMUSG00000042608	Stk40	25.516498	6.391390434	2.947398	0.007826	0.035675
1012	ENSMUSG00000030577	Cd22	62.24315	6.384916781	3.136309	0.010276	0.042493
1013	ENSMUSG00000042541	Sem1	64.511396	6.379483378	3.151952	0.01054	0.04304
1014	ENSMUSG00000039521	Foxp3	63.142766	6.370433754	3.141245	0.010499	0.043012
1015	ENSMUSG00000021775	Nr1d2	113.38933	6.3585225	3.152435	0.01078	0.043444
1016	ENSMUSG00000032050	Rdx	62.062647	6.331464339	3.167409	0.011294	0.044703
1017	ENSMUSG00000020034	Tcp11l2	517.55117	6.330598782	3.156172	0.01115	0.04438
1018	ENSMUSG00000003153	Slc2a3	183.73555	6.324239234	2.925982	0.008162	0.03695
1019	ENSMUSG00000016409	Nkap	25.31083	6.312308968	2.994379	0.009165	0.039916
1020	ENSMUSG00000019854	Reps1	59.136228	6.289185317	3.157062	0.011652	0.045766
1021	ENSMUSG00000051978	Erich1	22.98145	6.285360886	2.932473	0.00861	0.038624
1022	ENSMUSG00000042742	Bmt2	25.994962	6.249163174	3.057767	0.010712	0.043378
1023	ENSMUSG00000052298	Cdc42se2	270.75791	6.245895157	3.219532	0.0131	0.048534
1024	ENSMUSG00000055319	Sec23ip	57.285395	6.23528605	3.151412	0.012235	0.047225
1025	ENSMUSG00000024660	Incenp	54.281672	6.219235923	3.120332	0.011977	0.046529
1026	ENSMUSG00000031362	Xlr4c	1806.2321	6.211128113	3.010285	0.01046	0.04298
1027	ENSMUSG00000017776	Crk	70.849894	6.205853245	2.939889	0.009508	0.04065
1028	ENSMUSG00000006411	Nectin4	54.701259	6.205678541	3.149902	0.01259	0.048166

1029	ENSMUSG00000024927	Rela	55.386685	6.19838163	3.148842	0.012669	0.048241
1030	ENSMUSG00000049606	Zfp644	54.046576	6.192536203	3.132623	0.012499	0.047986
1031	ENSMUSG00000009905	Kdsr	140.5346	6.189918821	3.159266	0.012938	0.048441
1032	ENSMUSG00000020739	Nup85	52.387538	6.158183514	3.13033	0.012913	0.048441
1033	ENSMUSG00000067288	Rps28	51.538341	6.157085799	3.119723	0.012763	0.048441
1034	ENSMUSG00000017307	Acot8	106.28078	6.147778701	3.129818	0.013044	0.048534
1035	ENSMUSG00000031666	Rbl2	49.86384	6.142552527	3.118709	0.012939	0.048441
1036	ENSMUSG00000035842	Ddx11	50.028067	6.133071965	3.112009	0.01296	0.048473
1037	ENSMUSG00000031386	Hcfc1	22.471567	6.10372218	3.03722	0.012157	0.047089
1038	ENSMUSG00000031320	Rps4x	47.776009	6.098859181	3.113447	0.013447	0.049399
1039	ENSMUSG00000035992	Fnip1	602.43855	6.045124496	2.908333	0.01082	0.043503
1040	ENSMUSG00000057329	Bcl2	55.921995	6.035409723	2.78329	0.00898	0.03951
1041	ENSMUSG00000058355	Abce1	20.759261	6.016857606	3.031148	0.01321	0.048746
1042	ENSMUSG00000048865	Arhgap30	272.17809	5.996588971	2.944444	0.012011	0.046616
1043	ENSMUSG00000026987	Baz2b	213.20555	5.988194346	2.765941	0.009203	0.039954
1044	ENSMUSG00000016534	Lamp2	588.95524	5.986669889	2.975359	0.012667	0.048241
1045	ENSMUSG00000041697	Cox6a1	212.6409	5.960941767	2.878192	0.011342	0.044779
1046	ENSMUSG00000041966	Dcaf17	30.1175	5.951984489	2.829531	0.010633	0.043149
1047	ENSMUSG00000040687	Madd	55.075203	5.944295249	2.907925	0.012062	0.046769
1048	ENSMUSG00000027309	Dnaaf9	283.2453	5.934534808	2.955224	0.013024	0.048534
1049	ENSMUSG00000022799	Arhgap31	54.279146	5.907805418	2.921327	0.012785	0.048441
1050	ENSMUSG00000026743	Mllt10	524.03857	5.894539464	2.53484	0.006622	0.030953
1051	ENSMUSG00000020982	Nemf	698.35237	5.781409511	2.470007	0.006624	0.030953
1052	ENSMUSG00000035202	Lars2	763.93937	5.686644021	2.515744	0.008223	0.037117
1053	ENSMUSG00000049421	Zfp260	212.63775	5.440384804	2.529178	0.011343	0.044779
1054	ENSMUSG00000064370	CYTB	1470460.3	3.276300941	1.236273	0.004682	0.02623

Downregulated Transcripts = 17

	Ensembl	Symbol	baseMean	log2FoldChange	lfcSE	pvalue	padj
1	ENSMUSG00000040274	Cdk6	52681.078	-2.538354673	1.108905	0.011804	0.046087
2	ENSMUSG00000021877	Arf4	18746.517	-3.787732352	1.220737	0.001216	0.016881
3	ENSMUSG00000053044	Cd8b1	13055.041	-4.37534852	1.772473	0.006687	0.031128
4	ENSMUSG00000078771	Evi2a	7132.338	-6.372374412	3.320284	0.012872	0.048441
5	ENSMUSG00000042901	Aida	692.0926	-6.382612601	2.716382	0.005265	0.027351
6	ENSMUSG00000040463	Mybbp1a	407.80583	-6.67063908	2.957053	0.005779	0.028661
7	ENSMUSG00000022673	Mcm4	19178.714	-6.945870637	2.224362	0.00053	0.016863
8	ENSMUSG00000039671	Zmynd8	1774.2464	-7.061028491	2.802555	0.002668	0.020848
9	ENSMUSG00000042156	Dzip1	566.17507	-7.11007654	2.931117	0.003256	0.02241
10	ENSMUSG00000034028	Cd226	7368.7698	-7.111633528	3.357733	0.006262	0.029976
11	ENSMUSG00000094796	BC147527	230.83145	-7.390498033	3.239428	0.003916	0.024156
12	ENSMUSG00000070501	Ifi214	10608.897	-7.993430376	2.582243	0.000359	0.014436
13	ENSMUSG00000039220	Ppp1r10	7680.4581	-8.015729251	2.097036	2.87E-05	0.002131
14	ENSMUSG00000036390	Gadd45a	9750.7944	-8.178815424	2.561404	0.000242	0.011829
15	ENSMUSG00000043263	Ifi209	2064.4452	-8.362328476	2.419779	9.23E-05	0.005393
16	ENSMUSG00000003812	Dnase2a	814.50982	-9.01155852	3.30224	0.000567	0.016863
17	ENSMUSG00000087231	E230016M	3379.0132	-10.44117964	3.827139	0.000241	0.011829
