# Supplementary material for: Reactivation of low avidity tumor-specific CD8+ T cells associates with immunotherapeutic efficacy of anti-PD-1
Source: J Immunother Cancer. 2023 Aug 16;11(8):e007114. doi: 10.1136/jitc-2023-007114 (PMC10432680; doi:10.1136/jitc-2023-007114)
Supplement: Supplementary data [file jitc-2023-007114supp003.pdf]

Reactome Pathway Analysis

TetLowReg vs TetHighProg

ID	Description	enrichmentScore	NES	pvalue	p.adjust	qvalues
R-HSA-69620	Cell Cycle Checkpoints	0.667810526	3.060889	1.00E-10	4.20E-09	3.12E-09
R-HSA-453279	Mitotic G1 phase and G1/S transition	0.701604043	2.977724	1.00E-10	4.20E-09	3.12E-09
R-HSA-69278	Cell Cycle, Mitotic	0.607764161	2.956059	1.00E-10	4.20E-09	3.12E-09
R-HSA-69306	DNA Replication	0.696851078	2.945683	1.00E-10	4.20E-09	3.12E-09
R-HSA-69206	G1/S Transition	0.704686584	2.916725	1.00E-10	4.20E-09	3.12E-09
R-HSA-2555396	Mitotic Metaphase and Anaphase	0.649970842	2.908547	1.00E-10	4.20E-09	3.12E-09
R-HSA-69239	Synthesis of DNA	0.712418645	2.902836	1.00E-10	4.20E-09	3.12E-09
R-HSA-68882	Mitotic Anaphase	0.648162882	2.899854	1.00E-10	4.20E-09	3.12E-09
R-HSA-69481	G2/M Checkpoints	0.687690509	2.895866	1.00E-10	4.20E-09	3.12E-09
R-HSA-69242	S Phase	0.656346085	2.782889	1.00E-10	4.20E-09	3.12E-09
R-HSA-69002	DNA Replication Pre-Initiation	0.68178804	2.78006	1.00E-10	4.20E-09	3.12E-09
R-HSA-69618	Mitotic Spindle Checkpoint	0.701925518	2.769385	1.00E-10	4.20E-09	3.12E-09
R-HSA-2467813	Separation of Sister Chromatids	0.642306064	2.764014	1.00E-10	4.20E-09	3.12E-09
R-HSA-141424	Amplification of signal from the kinetochores	0.711166744	2.735057	1.00E-10	4.20E-09	3.12E-09
R-HSA-141444	Amplification of signal from unattached kinetochores via a MAD2 inhibitory signal	0.711166744	2.735057	1.00E-10	4.20E-09	3.12E-09
R-HSA-2500257	Resolution of Sister Chromatid Cohesion	0.669933533	2.709248	1.00E-10	4.20E-09	3.12E-09
R-HSA-69052	Switching of origins to a post-replicative state	0.696782777	2.696243	1.00E-10	4.20E-09	3.12E-09
R-HSA-176814	Activation of APC/C and APC/C:Cdc20 mediated degradation of mitotic proteins	0.722151072	2.694526	1.00E-10	4.20E-09	3.12E-09
R-HSA-68962	Activation of the pre-replicative complex	0.842361082	2.689138	1.00E-10	4.20E-09	3.12E-09
R-HSA-176409	APC/C:Cdc20 mediated degradation of mitotic proteins	0.716866842	2.65005	1.00E-10	4.20E-09	3.12E-09
R-HSA-174143	APC/C-mediated degradation of cell cycle proteins	0.694024097	2.631884	1.00E-10	4.20E-09	3.12E-09
R-HSA-453276	Regulation of mitotic cell cycle	0.694024097	2.631884	1.00E-10	4.20E-09	3.12E-09
R-HSA-68949	Orc1 removal from chromatin	0.704924368	2.605902	1.00E-10	4.20E-09	3.12E-09
R-HSA-68867	Assembly of the pre-replicative complex	0.652233063	2.601606	1.00E-10	4.20E-09	3.12E-09
R-HSA-179419	APC:Cdc20 mediated degradation of cell cycle proteins prior to satisfation of the cell cycle checkpoint	0.703757258	2.596975	1.00E-10	4.20E-09	3.12E-09
R-HSA-9648025	EML4 and NUDC in mitotic spindle formation	0.648826469	2.595882	1.00E-10	4.20E-09	3.12E-09
R-HSA-68886	M Phase	0.548567319	2.585749	1.00E-10	4.20E-09	3.12E-09

R-HSA-176408	Regulation of APC/C activators between G1/S and early anaphase	0.678680472	2.54635	1.00E-10	4.20E-09	3.12E-09
R-HSA-174184	Cdc20:Phospho-APC/C mediated degradation of Cyclin A	0.694196425	2.53527	1.00E-10	4.20E-09	3.12E-09
R-HSA-174178	APC/C:Cdh1 mediated degradation of Cdc20 and other APC/C:Cdh1 targeted proteins in late mitosis/early G1	0.686814463	2.534454	1.00E-10	4.20E-09	3.12E-09
R-HSA-5663220	RHO GTPases Activate Formins	0.604366777	2.48191	1.00E-10	4.20E-09	3.12E-09
R-HSA-8852276	The role of GTSE1 in G2/M progression after G2 checkpoint	0.661789186	2.462604	1.00E-10	4.20E-09	3.12E-09
R-HSA-68877	Mitotic Prometaphase	0.557460989	2.437994	1.00E-10	4.20E-09	3.12E-09
R-HSA-162909	Host Interactions of HIV factors	0.586018736	2.404696	1.00E-10	4.20E-09	3.12E-09
R-HSA-195258	RHO GTPase Effectors	0.477118561	2.19199	1.00E-10	4.20E-09	3.12E-09
R-HSA-453274	Mitotic G2-G2/M phases	0.506046769	2.210552	1.36E-10	5.56E-09	4.13E-09
R-HSA-176187	Activation of ATR in response to replication stress	0.794124334	2.607497	2.08E-10	8.27E-09	6.15E-09
R-HSA-69017	CDK-mediated phosphorylation and removal of Cdc6	0.672431703	2.455783	2.44E-10	9.44E-09	7.01E-09
R-HSA-69205	G1/S-Specific Transcription	0.848709047	2.594319	4.15E-10	1.57E-08	1.16E-08
R-HSA-69190	DNA strand elongation	0.799258704	2.551539	6.98E-10	2.57E-08	1.91E-08
R-HSA-174154	APC/C:Cdc20 mediated degradation of Securin	0.676780797	2.433779	8.30E-10	2.98E-08	2.21E-08
R-HSA-162906	HIV Infection	0.464526216	2.092425	8.90E-10	3.12E-08	2.32E-08
R-HSA-5693538	Homology Directed Repair	0.562871631	2.267544	9.62E-10	3.29E-08	2.45E-08
R-HSA-69275	G2/M Transition	0.498949178	2.180049	1.05E-09	3.52E-08	2.62E-08
R-HSA-9759194	Nuclear events mediated by NFE2L2	0.619366941	2.341625	1.19E-09	3.88E-08	2.88E-08
R-HSA-1474244	Extracellular matrix organization	-0.459038783	-1.94501	3.33E-09	1.07E-07	7.91E-08
R-HSA-69601	Ubiquitin Mediated Degradation of Phosphorylated Cdc25A	0.701413015	2.434975	3.70E-09	1.11E-07	8.26E-08
R-HSA-69610	p53-Independent DNA Damage Response	0.701413015	2.434975	3.70E-09	1.11E-07	8.26E-08
R-HSA-69613	p53-Independent G1/S DNA damage checkpoint	0.701413015	2.434975	3.70E-09	1.11E-07	8.26E-08
R-HSA-1538133	G0 and Early G1	0.830096886	2.508574	4.53E-09	1.33E-07	9.91E-08
R-HSA-69615	G1/S DNA Damage Checkpoints	0.626658492	2.293745	7.27E-09	2.10E-07	1.56E-07
R-HSA-5693567	HDR through Homologous Recombination (HRR) or Single Strand Annealing (SSA)	0.559319328	2.238108	9.06E-09	2.56E-07	1.90E-07
R-HSA-9755511	KEAP1-NFE2L2 pathway	0.561089393	2.238055	9.57E-09	2.66E-07	1.97E-07
R-HSA-3000178	ECM proteoglycans	-0.626250405	-2.24313	1.09E-08	2.98E-07	2.21E-07
R-HSA-69273	Cyclin A/B1/B2 associated events during G2/M transition	0.815960338	2.494214	1.45E-08	3.88E-07	2.89E-07

R-HSA-5689603	UCH proteinases	0.586555396	2.269711	1.50E-08	3.93E-07	2.92E-07
R-HSA-180585	Vif-mediated degradation of APOBEC3G	0.678381486	2.38316	1.76E-08	4.53E-07	3.37E-07
R-HSA-174084	Autodegradation of Cdh1 by Cdh1:APC/C	0.651428764	2.355271	1.90E-08	4.82E-07	3.58E-07
R-HSA-1236975	Antigen processing-Cross presentation	0.550517965	2.212684	2.15E-08	5.37E-07	3.99E-07
R-HSA-8941858	Regulation of RUNX3 expression and activity	0.667352391	2.368649	2.27E-08	5.56E-07	4.13E-07
R-HSA-69656	Cyclin A:Cdk2-associated events at S phase entry	0.585164102	2.237301	2.63E-08	6.35E-07	4.72E-07
R-HSA-5693532	DNA Double-Strand Break Repair	0.498910079	2.092496	2.79E-08	6.63E-07	4.92E-07
R-HSA-9604323	Negative regulation of NOTCH4 signaling	0.653129759	2.32085	3.01E-08	7.03E-07	5.22E-07
R-HSA-351202	Metabolism of polyamines	0.651728767	2.32622	3.56E-08	8.19E-07	6.08E-07
R-HSA-69202	Cyclin E associated events during G1/S transition	0.577374828	2.201874	5.00E-08	1.12E-06	8.34E-07
R-HSA-73886	Chromosome Maintenance	0.5477522	2.200203	5.04E-08	1.12E-06	8.34E-07
R-HSA-6798695	Neutrophil degranulation	0.353318458	1.706598	5.36E-08	1.18E-06	8.73E-07
R-HSA-174113	SCF-beta-TrCP mediated degradation of Emi1	0.660752217	2.355526	6.95E-08	1.48E-06	1.10E-06
R-HSA-5668541	TNFR2 non-canonical NF-kB pathway	0.549799377	2.169185	6.91E-08	1.48E-06	1.10E-06
R-HSA-1236974	ER-Phagosome pathway	0.568898141	2.238316	8.85E-08	1.86E-06	1.38E-06
R-HSA-176974	Unwinding of DNA	0.921018904	2.264702	9.55E-08	1.98E-06	1.47E-06
R-HSA-169911	Regulation of Apoptosis	0.668033186	2.319096	1.02E-07	2.08E-06	1.54E-06
R-HSA-179409	APC-Cdc20 mediated degradation of Nek2A	0.825668213	2.356239	1.25E-07	2.52E-06	1.88E-06
R-HSA-202403	TCR signaling	0.520653801	2.110036	1.28E-07	2.55E-06	1.89E-06
R-HSA-211733	Regulation of activated PAK-2p34 by proteasome mediated degradation	0.676778359	2.329882	1.41E-07	2.76E-06	2.05E-06
R-HSA-156711	Polo-like kinase mediated events	0.902137473	2.30174	1.45E-07	2.81E-06	2.09E-06
R-HSA-450408	AUF1 (hnRNP D0) binds and destabilizes mRNA	0.662256542	2.306878	1.49E-07	2.85E-06	2.11E-06
R-HSA-1234176	Oxygen-dependent proline hydroxylation of Hypoxia-inducible Factor Alpha	0.61948996	2.242887	1.67E-07	3.14E-06	2.33E-06
R-HSA-73894	DNA Repair	0.394553423	1.819252	2.06E-07	3.83E-06	2.84E-06
R-HSA-983189	Kinesins	0.670376787	2.27989	2.30E-07	4.23E-06	3.14E-06
R-HSA-75815	Ubiquitin-dependent degradation of Cyclin D	0.655442569	2.275388	2.36E-07	4.28E-06	3.18E-06
R-HSA-5678895	Defective CFTR causes cystic fibrosis	0.629848557	2.248123	2.43E-07	4.36E-06	3.24E-06
R-HSA-3700989	Transcriptional Regulation by TP53	0.372879268	1.747103	2.93E-07	5.19E-06	3.85E-06
R-HSA-2129379	Molecules associated with elastic fibres	-0.714568266	-2.2077	3.15E-07	5.51E-06	4.10E-06
R-HSA-8854050	FBXL7 down-regulates AURKA during mitotic entry and in early mitosis	0.640758075	2.250989	3.70E-07	6.40E-06	4.75E-06
R-HSA-1566948	Elastic fibre formation	-0.69067058	-2.20225	3.88E-07	6.56E-06	4.87E-06

R-HSA-5693607	Processing of DNA double-strand break ends	0.580659786	2.170593	3.83E-07	6.56E-06	4.87E-06
R-HSA-69563	p53-Dependent G1 DNA Damage Response	0.596321388	2.173974	4.43E-07	7.33E-06	5.44E-06
R-HSA-69580	p53-Dependent G1/S DNA damage checkpoint	0.596321388	2.173974	4.43E-07	7.33E-06	5.44E-06
R-HSA-1236978	Cross-presentation of soluble exogenous antigens (endosomes)	0.650895473	2.227868	4.57E-07	7.47E-06	5.55E-06
R-HSA-187577	SCF(Skp2)-mediated degradation of p27/p21	0.61248473	2.202563	4.74E-07	7.62E-06	5.66E-06
R-HSA-69473	G2/M DNA damage checkpoint	0.589815804	2.176513	4.76E-07	7.62E-06	5.66E-06
R-HSA-2995410	Nuclear Envelope (NE) Reassembly	0.595512145	2.174866	5.15E-07	8.15E-06	6.06E-06
R-HSA-68875	Mitotic Prophase	0.516618237	2.06724	5.68E-07	8.88E-06	6.60E-06
R-HSA-5610783	Degradation of GLI2 by the proteasome	0.608007022	2.190168	6.20E-07	9.60E-06	7.13E-06
R-HSA-5676590	NIK-->noncanonical NF-kB signaling	0.605484056	2.18108	7.59E-07	1.16E-05	8.64E-06
R-HSA-9762114	GSK3B and BTRC:CUL1-mediated-degradation of NFE2L2	0.632786265	2.222984	8.01E-07	1.21E-05	9.02E-06
R-HSA-349425	Autodegradation of the E3 ubiquitin ligase COP1	0.639578561	2.213681	9.09E-07	1.36E-05	1.01E-05
R-HSA-8948216	Collagen chain trimerization	-0.711138941	-2.16908	9.62E-07	1.43E-05	1.06E-05
R-HSA-5658442	Regulation of RAS by GAPs	0.595782181	2.146089	1.01E-06	1.49E-05	1.11E-05
R-HSA-202424	Downstream TCR signaling	0.521454937	2.048592	1.04E-06	1.51E-05	1.12E-05
R-HSA-350562	Regulation of ornithine decarboxylase (ODC)	0.633084918	2.191206	1.39E-06	2.00E-05	1.49E-05
R-HSA-5607761	Dectin-1 mediated noncanonical NF-kB signaling	0.597765843	2.16125	1.58E-06	2.26E-05	1.68E-05
R-HSA-5610780	Degradation of GLI1 by the proteasome	0.59605239	2.147105	1.64E-06	2.32E-05	1.72E-05
R-HSA-1234174	Cellular response to hypoxia	0.562655717	2.103291	1.69E-06	2.37E-05	1.76E-05
R-HSA-69541	Stabilization of p53	0.603119736	2.143143	1.98E-06	2.75E-05	2.05E-05
R-HSA-2132295	MHC class II antigen presentation	0.495681823	1.999579	2.08E-06	2.86E-05	2.12E-05
R-HSA-4641257	Degradation of AXIN	0.618006674	2.171063	2.21E-06	3.01E-05	2.24E-05
R-HSA-180534	Vpu mediated degradation of CD4	0.6210507	2.155995	2.33E-06	3.14E-05	2.33E-05
R-HSA-4615885	SUMOylation of DNA replication proteins	0.63179861	2.159379	2.52E-06	3.34E-05	2.48E-05
R-HSA-6811434	COPI-dependent Golgi-to-ER retrograde traffic	0.535607	2.024956	2.51E-06	3.34E-05	2.48E-05
R-HSA-113510	E2F mediated regulation of DNA replication	0.779273653	2.25339	2.68E-06	3.53E-05	2.62E-05
R-HSA-70171	Glycolysis	0.564234318	2.105299	3.36E-06	4.37E-05	3.24E-05
R-HSA-174048	APC/C:Cdc20 mediated degradation of Cyclin B	0.808113478	2.235693	3.94E-06	5.08E-05	3.78E-05
R-HSA-9645723	Diseases of programmed cell death	0.568157411	2.071298	4.26E-06	5.40E-05	4.01E-05
R-HSA-450531	Regulation of mRNA stability by proteins that bind AU-rich elements	0.533171322	2.021896	4.23E-06	5.40E-05	4.01E-05
R-HSA-6783783	Interleukin-10 signaling	0.6333692	2.154031	4.52E-06	5.68E-05	4.22E-05
R-HSA-5362768	Hh mutants are degraded by ERAD	0.608562	2.137884	4.72E-06	5.87E-05	4.36E-05

R-HSA-157118	Signaling by NOTCH	0.407030873	1.789421	4.75E-06	5.87E-05	4.36E-05
R-HSA-9711123	Cellular response to chemical stress	0.411271641	1.796956	6.99E-06	8.50E-05	6.31E-05
R-HSA-449147	Signaling by Interleukins	0.32073699	1.548114	6.98E-06	8.50E-05	6.31E-05
R-HSA-3000157	Laminin interactions	-0.704265176	-2.10781	7.87E-06	9.48E-05	7.04E-05
R-HSA-5685942	HDR through Homologous Recombination (HRR)	0.572933363	2.063826	8.61E-06	0.000103	7.65E-05
R-HSA-5610785	GLI3 is processed to GLI3R by the proteasome	0.568343683	2.047293	1.09E-05	0.000129	9.58E-05
R-HSA-1169091	Activation of NF-kappaB in B cells	0.540399222	1.978012	1.15E-05	0.000135	0.0001
R-HSA-5687128	MAPK6/MAPK4 signaling	0.514728294	1.967999	1.25E-05	0.000146	0.000109
R-HSA-380108	Chemokine receptors bind chemokines	0.592561043	2.11243	1.32E-05	0.000152	0.000113
R-HSA-8856688	Golgi-to-ER retrograde transport	0.462266761	1.872746	1.62E-05	0.000186	0.000139
R-HSA-4641258	Degradation of DVL	0.580828495	2.061548	1.78E-05	0.000203	0.000151
R-HSA-5387390	Hh mutants abrogate ligand secretion	0.56762516	2.017015	1.86E-05	0.00021	0.000156
R-HSA-5688426	Deubiquitination	0.37140513	1.705344	1.87E-05	0.00021	0.000156
R-HSA-1168372	Downstream signaling events of B Cell Receptor (BCR)	0.507163627	1.916933	2.01E-05	0.000224	0.000166
R-HSA-2995383	Initiation of Nuclear Envelope (NE) Reformation	0.782867577	2.165849	2.05E-05	0.000227	0.000168
R-HSA-8948751	Regulation of PTEN stability and activity	0.537750445	1.984384	2.18E-05	0.000237	0.000176
R-HSA-5357801	Programmed Cell Death	0.390067153	1.732392	2.17E-05	0.000237	0.000176
R-HSA-3000171	Non-integrin membrane-ECM interactions	-0.58633518	-2.00378	2.62E-05	0.000283	0.00021
R-HSA-2980766	Nuclear Envelope Breakdown	0.602789514	2.075167	2.78E-05	0.000298	0.000222
R-HSA-141405	Inhibition of the proteolytic activity of APC/C required for the onset of anaphase by mitotic spindle checkpoint components	0.814870822	2.136996	2.90E-05	0.000307	0.000228
R-HSA-141430	Inactivation of APC/C via direct inhibition of the APC/C complex	0.814870822	2.136996	2.90E-05	0.000307	0.000228
R-HSA-606279	Deposition of new CENPA-containing nucleosomes at the centromere	0.628728272	2.090861	3.33E-05	0.000345	0.000256
R-HSA-774815	Nucleosome assembly	0.628728272	2.090861	3.33E-05	0.000345	0.000256
R-HSA-1650814	Collagen biosynthesis and modifying enzymes	-0.59155134	-2.00971	3.30E-05	0.000345	0.000256
R-HSA-109581	Apoptosis	0.40164233	1.753831	3.63E-05	0.000373	0.000277
R-HSA-5693579	Homologous DNA Pairing and Strand Exchange	0.618324	2.044663	4.20E-05	0.000426	0.000316
R-HSA-913531	Interferon Signaling	0.384861758	1.690656	4.18E-05	0.000426	0.000316
R-HSA-1362277	Transcription of E2F targets under negative control by DREAM complex	0.785398697	2.099291	4.63E-05	0.000466	0.000346
R-HSA-162658	Golgi Cisternae Pericentriolar Stack Reorganization	0.804876449	2.109995	5.31E-05	0.000528	0.000392

R-HSA-9675135	Diseases of DNA repair	0.577310393	1.982104	5.30E-05	0.000528	0.000392
R-HSA-8874081	MET activates PTK2 signaling	-0.677135417	-2.00071	5.93E-05	0.000586	0.000435
R-HSA-2871837	FCERI mediated NF-kB activation	0.49407898	1.857156	6.29E-05	0.000616	0.000458
R-HSA-157579	Telomere Maintenance	0.488033119	1.865933	7.34E-05	0.000715	0.000531
R-HSA-3560782	Diseases associated with glycosaminoglycan metabolism	-0.629869088	-1.94602	7.82E-05	0.000756	0.000562
R-HSA-2559583	Cellular Senescence	0.412072172	1.752052	8.12E-05	0.000781	0.00058
R-HSA-176412	Phosphorylation of the APC/C	0.798536591	2.093375	8.19E-05	0.000782	0.000581
R-HSA-216083	Integrin cell surface interactions	-0.512310292	-1.84981	8.83E-05	0.000838	0.000622
R-HSA-2514853	Condensation of Prometaphase Chromosomes	0.855450178	2.039858	9.98E-05	0.000935	0.000694
R-HSA-6803157	Antimicrobial peptides	0.595424933	1.995686	9.94E-05	0.000935	0.000694
R-HSA-5619084	ABC transporter disorders	0.502721853	1.870694	0.000102	0.000952	0.000707
R-HSA-5689880	Ub-specific processing proteases	0.384491518	1.680683	0.000105	0.000972	0.000722
R-HSA-9710421	Defective pyroptosis	0.622286104	2.024696	0.000112	0.001031	0.000766
R-HSA-8939902	Regulation of RUNX2 expression and activity	0.504549788	1.886082	0.000113	0.001031	0.000766
R-HSA-9675136	Diseases of DNA Double-Strand Break Repair	0.593956442	1.941688	0.00012	0.001079	0.000802
R-HSA-9701190	Defective homologous recombination repair (HRR) due to BRCA2 loss of function	0.593956442	1.941688	0.00012	0.001079	0.000802
R-HSA-9020702	Interleukin-1 signaling	0.438256333	1.765528	0.000121	0.001083	0.000804
R-HSA-4608870	Asymmetric localization of PCP proteins	0.522791751	1.880017	0.000129	0.001153	0.000857
R-HSA-177243	Interactions of Rev with host cellular proteins	0.612810642	2.009889	0.000139	0.001236	0.000918
R-HSA-2022090	Assembly of collagen fibrils and other multimeric structures	-0.560062064	-1.91137	0.000146	0.001286	0.000955
R-HSA-2299718	Condensation of Prophase Chromosomes	0.617185401	2.02652	0.000156	0.001362	0.001012
R-HSA-909733	Interferon alpha/beta signaling	0.482810434	1.8054	0.000156	0.001362	0.001012
R-HSA-5693616	Presynaptic phase of homologous DNA pairing and strand exchange	0.596286668	1.945318	0.000169	0.00146	0.001084
R-HSA-6784531	tRNA processing in the nucleus	0.55436821	1.924505	0.000187	0.001606	0.001193
R-HSA-1474290	Collagen formation	-0.509748339	-1.82584	0.000192	0.001643	0.00122
R-HSA-8939236	RUNX1 regulates transcription of genes involved in differentiation of HSCs	0.460749888	1.812809	0.000195	0.001654	0.001229
R-HSA-165054	Rev-mediated nuclear export of HIV RNA	0.607151136	1.975453	0.000206	0.001745	0.001296
R-HSA-2559586	DNA Damage/Telomere Stress Induced Senescence	0.522110778	1.855283	0.000219	0.001843	0.001369
R-HSA-2022857	Keratan sulfate degradation	-0.772728596	-1.8666	0.000223	0.001853	0.001376
R-HSA-6804756	Regulation of TP53 Activity through Phosphorylation	0.456030834	1.753837	0.000222	0.001853	0.001376

R-HSA-1362300	Transcription of E2F targets under negative control by p107 (RBL1) and p130 (RBL2) in complex with HDAC1	0.787333244	2.008825	0.000229	0.00189	0.001404
R-HSA-75035	Chk1/Chk2(Cds1) mediated inactivation of Cyclin B:Cdk1 complex	0.805670817	2.009422	0.000231	0.001901	0.001412
R-HSA-983231	Factors involved in megakaryocyte development and platelet production	0.400944387	1.67343	0.000236	0.001928	0.001432
R-HSA-15869	Metabolism of nucleotides	0.472075891	1.78675	0.000256	0.002079	0.001544
R-HSA-9609690	HCMV Early Events	0.468050577	1.832593	0.000276	0.002231	0.001657
R-HSA-6791312	TP53 Regulates Transcription of Cell Cycle Genes	0.565527382	1.923062	0.000279	0.002246	0.001669
R-HSA-9609646	HCMV Infection	0.428302601	1.721467	0.000304	0.00243	0.001805
R-HSA-5633007	Regulation of TP53 Activity	0.396447012	1.681843	0.000319	0.002536	0.001883
R-HSA-9709570	Impaired BRCA2 binding to RAD51	0.605662454	1.933505	0.000358	0.002835	0.002106
R-HSA-195253	Degradation of beta-catenin by the destruction complex	0.452680466	1.72634	0.000386	0.003034	0.002254
R-HSA-1169410	Antiviral mechanism by IFN-stimulated genes	0.464181455	1.75687	0.000391	0.003061	0.002274
R-HSA-8875878	MET promotes cell motility	-0.596703671	-1.88996	0.000398	0.003099	0.002302
R-HSA-73884	Base Excision Repair	0.496415414	1.809753	0.000402	0.003114	0.002313
R-HSA-70326	Glucose metabolism	0.447216567	1.759909	0.000423	0.003256	0.002418
R-HSA-180910	Vpr-mediated nuclear import of PICs	0.594471869	1.902617	0.000445	0.003406	0.00253
R-HSA-6804114	TP53 Regulates Transcription of Genes Involved in G2 Cell Cycle Arrest	0.715701194	1.935062	0.000452	0.003447	0.002561
R-HSA-180746	Nuclear import of Rev protein	0.593828928	1.900559	0.000456	0.003454	0.002566
R-HSA-5578749	Transcriptional regulation by small RNAs	0.480330328	1.758143	0.000465	0.003505	0.002604
R-HSA-180786	Extension of Telomeres	0.527071532	1.804046	0.000504	0.003783	0.00281
R-HSA-191859	snRNP Assembly	0.543180657	1.869958	0.000514	0.00382	0.002837
R-HSA-194441	Metabolism of non-coding RNA	0.543180657	1.869958	0.000514	0.00382	0.002837
R-HSA-9610379	HCMV Late Events	0.48928594	1.783761	0.000558	0.004125	0.003064
R-HSA-9013694	Signaling by NOTCH4	0.444643169	1.695689	0.000564	0.00415	0.003082
R-HSA-446652	Interleukin-1 family signaling	0.374906093	1.579419	0.000574	0.004204	0.003123
R-HSA-9006934	Signaling by Receptor Tyrosine Kinases	-0.316394136	-1.41196	0.000666	0.00485	0.003603
R-HSA-176033	Interactions of Vpr with host cellular proteins	0.583171404	1.912678	0.000686	0.00497	0.003692
R-HSA-69186	Lagging Strand Synthesis	0.67000191	1.912008	0.000691	0.004985	0.003703
R-HSA-2559582	Senescence-Associated Secretory Phenotype (SASP)	0.464948911	1.714409	0.000698	0.005011	0.003722
R-HSA-983705	Signaling by the B Cell Receptor (BCR)	0.412964664	1.65879	0.000727	0.005195	0.003859

R-HSA-5607764	CLEC7A (Dectin-1) signaling	0.426774707	1.684992	0.000743	0.00528	0.003922
R-HSA-72306	tRNA processing	0.440168677	1.731833	0.00076	0.005351	0.003975
R-HSA-211000	Gene Silencing by RNA	0.43861089	1.697646	0.00076	0.005351	0.003975
R-HSA-163685	Integration of energy metabolism	-0.448268032	-1.66682	0.000798	0.005591	0.004153
R-HSA-5693537	Resolution of D-Loop Structures	0.598345447	1.85287	0.000851	0.005934	0.004407
R-HSA-388841	Costimulation by the CD28 family	0.478215845	1.743404	0.000858	0.005956	0.004424
R-HSA-162587	HIV Life Cycle	0.37692623	1.587237	0.000875	0.00604	0.004486
R-HSA-9020591	Interleukin-12 signaling	0.549655755	1.827902	0.001045	0.007154	0.005314
R-HSA-381426	Regulation of Insulin-like Growth Factor (IGF) transport and uptake by Insulin-like Growth Factor Binding Proteins (IGFBPs)	-0.432341971	-1.64346	0.001046	0.007154	0.005314
R-HSA-4090294	SUMOylation of intracellular receptors	-0.618569621	-1.85133	0.001058	0.007204	0.005351
R-HSA-1428517	The citric acid (TCA) cycle and respiratory electron transport	0.36177708	1.537578	0.001112	0.007538	0.005599
R-HSA-8950505	Gene and protein expression by JAK-STAT signaling after Interleukin-12 stimulation	0.575925775	1.838575	0.001143	0.007715	0.00573
R-HSA-5676594	TNF receptor superfamily (TNFSF) members mediating non-canonical NF-kB pathway	0.73284607	1.921167	0.001272	0.008535	0.006339
R-HSA-3301854	Nuclear Pore Complex (NPC) Disassembly	0.565021134	1.838377	0.001276	0.008535	0.006339
R-HSA-5693568	Resolution of D-loop Structures through Holliday Junction Intermediates	0.602846512	1.860117	0.001283	0.00854	0.006344
R-HSA-1169408	ISG15 antiviral mechanism	0.456192483	1.702168	0.001294	0.008571	0.006367
R-HSA-8957275	Post-translational protein phosphorylation	-0.446707015	-1.65378	0.001389	0.009164	0.006807
R-HSA-3781865	Diseases of glycosylation	-0.428844914	-1.62837	0.001397	0.009176	0.006815
R-HSA-389960	Formation of tubulin folding intermediates by CCT/TriC	0.650060927	1.879751	0.00148	0.009575	0.007112
R-HSA-8953750	Transcriptional Regulation by E2F6	0.580093883	1.827854	0.001484	0.009575	0.007112
R-HSA-5685938	HDR through Single Strand Annealing (SSA)	0.56063387	1.824102	0.001476	0.009575	0.007112
R-HSA-983169	Class I MHC mediated antigen processing & presentation	0.303000214	1.420302	0.001467	0.009575	0.007112
R-HSA-168274	Export of Viral Ribonucleoproteins from Nucleus	0.579015795	1.824457	0.001528	0.00973	0.007227
R-HSA-168333	NEP/NS2 Interacts with the Cellular Export Machinery	0.579015795	1.824457	0.001528	0.00973	0.007227
R-HSA-9675126	Diseases of mitotic cell cycle	0.551425896	1.764848	0.001528	0.00973	0.007227
R-HSA-446107	Type I hemidesmosome assembly	-0.775442663	-1.77403	0.001539	0.009759	0.007249

R-HSA-110314	Recognition of DNA damage by PCNA-containing replication complex	0.578718739	1.792093	0.001644	0.010376	0.007707
R-HSA-5693554	Resolution of D-loop Structures through Synthesis-Dependent Strand Annealing (SDSA)	0.609763911	1.881154	0.00168	0.010559	0.007843
R-HSA-176407	Conversion from APC/C:Cdc20 to APC/C:Cdh1 in late anaphase	0.722499018	1.894042	0.00172	0.010769	0.007999
R-HSA-174417	Telomere C-strand (Lagging Strand) Synthesis	0.567102593	1.810408	0.001731	0.010791	0.008015
R-HSA-73893	DNA Damage Bypass	0.521323209	1.772747	0.001762	0.010938	0.008124
R-HSA-113501	Inhibition of replication initiation of damaged DNA by RB1/E2F1	0.734389041	1.873742	0.001815	0.011216	0.008331
R-HSA-186797	Signaling by PDGF	-0.501708193	-1.71614	0.001854	0.011412	0.008477
R-HSA-4419969	Depolymerisation of the Nuclear Lamina	0.760705715	1.897275	0.001878	0.011461	0.008513
R-HSA-168276	NS1 Mediated Effects on Host Pathways	0.543800901	1.779266	0.001874	0.011461	0.008513
R-HSA-159230	Transport of the SLBP Dependant Mature mRNA	0.553517677	1.800948	0.00192	0.011672	0.00867
R-HSA-6804116	TP53 Regulates Transcription of Genes Involved in G1 Cell Cycle Arrest	0.754967836	1.882964	0.002087	0.012585	0.009348
R-HSA-1268020	Mitochondrial protein import	0.49287194	1.731464	0.00208	0.012585	0.009348
R-HSA-9648895	Response of EIF2AK1 (HRI) to heme deficiency	0.725542589	1.902733	0.002135	0.012702	0.009435
R-HSA-9701192	Defective HDR through Homologous Recombination (HRR) due to BRCA1 loss-of-function	0.607544627	1.836016	0.00215	0.012702	0.009435
R-HSA-9701193	Defective HDR through Homologous Recombination (HRR) due to PALB2 loss of function	0.607544627	1.836016	0.00215	0.012702	0.009435
R-HSA-9704331	Defective HDR through Homologous Recombination Repair (HRR) due to PALB2 loss of BRCA1 binding function	0.607544627	1.836016	0.00215	0.012702	0.009435
R-HSA-9704646	Defective HDR through Homologous Recombination Repair (HRR) due to PALB2 loss of BRCA2/RAD51/RAD51C binding function	0.607544627	1.836016	0.00215	0.012702	0.009435
R-HSA-159227	Transport of the SLBP independent Mature mRNA	0.540682546	1.730463	0.002264	0.013322	0.009895
R-HSA-447115	Interleukin-12 family signaling	0.476421381	1.648969	0.002385	0.013975	0.010381
R-HSA-422356	Regulation of insulin secretion	-0.47009326	-1.66636	0.002425	0.014157	0.010516
R-HSA-110373	Resolution of AP sites via the multiple-nucleotide patch replacement pathway	0.602818845	1.821734	0.002567	0.014923	0.011084
R-HSA-8878159	Transcriptional regulation by RUNX3	0.40279104	1.589176	0.002648	0.015273	0.011345

R-HSA-5621481	C-type lectin receptors (CLRs)	0.363333709	1.503239	0.002645	0.015273	0.011345
R-HSA-69166	Removal of the Flap Intermediate	0.704938931	1.848008	0.002727	0.015672	0.011641
R-HSA-9687139	Aberrant regulation of mitotic cell cycle due to RB1 defects	0.566636171	1.785449	0.002762	0.015808	0.011742
R-HSA-170822	Regulation of Glucokinase by Glucokinase Regulatory Protein	0.565455606	1.781729	0.002806	0.015937	0.011837
R-HSA-5619107	Defective TPR may confer susceptibility towards thyroid papillary carcinoma (TPC)	0.565455606	1.781729	0.002806	0.015937	0.011837
R-HSA-8852135	Protein ubiquitination	0.445320928	1.612301	0.002821	0.01596	0.011855
R-HSA-211859	Biological oxidations	-0.373331661	-1.49549	0.002871	0.016182	0.01202
R-HSA-381042	PERK regulates gene expression	0.564574484	1.778953	0.002939	0.016498	0.012255
R-HSA-375276	Peptide ligand-binding receptors	0.336116618	1.466851	0.003051	0.017064	0.012675
R-HSA-1474228	Degradation of the extracellular matrix	-0.414525683	-1.60256	0.003174	0.017684	0.013135
R-HSA-9615017	FOXO-mediated transcription of oxidative stress, metabolic and neuronal genes	-0.603283401	-1.70229	0.003214	0.017838	0.01325
R-HSA-6806834	Signaling by MET	-0.460734915	-1.63804	0.003232	0.017872	0.013275
R-HSA-6811442	Intra-Golgi and retrograde Golgi-to-ER traffic	0.338404495	1.463423	0.003381	0.018629	0.013837
R-HSA-5619115	Disorders of transmembrane transporters	0.348947891	1.50521	0.003398	0.018649	0.013852
R-HSA-3232142	SUMOylation of ubiquitinylation proteins	0.513556831	1.678856	0.00369	0.020178	0.014988
R-HSA-168271	Transport of Ribonucleoproteins into the Host Nucleus	0.556092331	1.752226	0.003822	0.020824	0.015468
R-HSA-5578775	Ion homeostasis	-0.513200623	-1.70457	0.003857	0.020934	0.015549
R-HSA-5632684	Hedgehog 'on' state	0.424920353	1.594493	0.003905	0.021118	0.015686
R-HSA-181430	Norepinephrine Neurotransmitter Release Cycle	-0.657041649	-1.70807	0.004133	0.022269	0.016541
R-HSA-196757	Metabolism of folate and pterines	0.73096234	1.823092	0.004153	0.022298	0.016562
R-HSA-5358346	Hedgehog ligand biogenesis	0.446042442	1.612687	0.004227	0.02261	0.016795
R-HSA-9615933	Postmitotic nuclear pore complex (NPC) reformation	0.573048827	1.767886	0.004291	0.022869	0.016987
R-HSA-159234	Transport of Mature mRNAs Derived from Intronless Transcripts	0.506263116	1.651626	0.004345	0.023074	0.017139
R-HSA-212300	PRC2 methylates histones and DNA	0.539377142	1.721897	0.00453	0.023972	0.017806
R-HSA-9709603	Impaired BRCA2 binding to PALB2	0.598778974	1.772195	0.004887	0.025619	0.01903
R-HSA-5334118	DNA methylation	0.576211508	1.741326	0.004894	0.025619	0.01903
R-HSA-912446	Meiotic recombination	0.477116531	1.630702	0.004863	0.025619	0.01903
R-HSA-159236	Transport of Mature mRNA derived from an Intron-Containing Transcript	0.425264393	1.556586	0.005056	0.026372	0.019589

R-HSA-198933	Immunoregulatory interactions between a Lymphoid and a non-Lymphoid cell	0.372760072	1.503713	0.005199	0.027025	0.020074
R-HSA-9616222	Transcriptional regulation of granulopoiesis	0.480961855	1.669673	0.005227	0.027073	0.020109
R-HSA-69091	Polymerase switching	0.681182744	1.78573	0.005309	0.027307	0.020283
R-HSA-69109	Leading Strand Synthesis	0.681182744	1.78573	0.005309	0.027307	0.020283
R-HSA-400042	Adrenaline,noradrenaline inhibits insulin secretion	-0.629016263	-1.75275	0.005428	0.027821	0.020665
R-HSA-68616	Assembly of the ORC complex at the origin of replication	0.535404313	1.679972	0.005465	0.027913	0.020733
R-HSA-8854518	AURKA Activation by TPX2	0.429896164	1.586385	0.005489	0.027937	0.020751
R-HSA-72202	Transport of Mature Transcript to Cytoplasm	0.418639921	1.56205	0.005631	0.028565	0.021217
R-HSA-3000170	Syndecan interactions	-0.592070626	-1.70552	0.005725	0.028939	0.021495
R-HSA-5576891	Cardiac conduction	-0.402853443	-1.5283	0.005747	0.028953	0.021506
R-HSA-73933	Resolution of Abasic Sites (AP sites)	0.528037089	1.731849	0.005931	0.029777	0.022118
R-HSA-69231	Cyclin D associated events in G1	0.490441682	1.688398	0.006007	0.029954	0.022249
R-HSA-69236	G1 Phase	0.490441682	1.688398	0.006007	0.029954	0.022249
R-HSA-397014	Muscle contraction	-0.35232688	-1.42977	0.006031	0.029973	0.022263
R-HSA-68884	Mitotic Telophase/Cytokinesis	0.687636662	1.754456	0.00606	0.030012	0.022292
R-HSA-110313	Translesion synthesis by Y family DNA polymerases bypasses lesions on DNA template	0.514621877	1.689754	0.006202	0.030616	0.022741
R-HSA-389958	Cooperation of Prefoldin and TriC/CCT in actin and tubulin folding	0.541449608	1.676683	0.006459	0.031775	0.023602
R-HSA-5651801	PCNA-Dependent Long Patch Base Excision Repair	0.604361761	1.724689	0.006585	0.032193	0.023913
R-HSA-4086400	PCP/CE pathway	0.392224446	1.543197	0.006588	0.032193	0.023913
R-HSA-112310	Neurotransmitter release cycle	-0.500293573	-1.6617	0.006686	0.03246	0.024111
R-HSA-2980736	Peptide hormone metabolism	-0.444701914	-1.59009	0.006677	0.03246	0.024111
R-HSA-5389840	Mitochondrial translation elongation	0.420863917	1.523754	0.006814	0.032972	0.024491
R-HSA-390918	Peroxisomal lipid metabolism	-0.580059548	-1.63676	0.006843	0.033001	0.024513
R-HSA-380994	ATF4 activates genes in response to endoplasmic reticulum stress	0.577886395	1.766473	0.007223	0.034723	0.025792
R-HSA-201681	TCF dependent signaling in response to WNT	0.320087147	1.397536	0.007639	0.036604	0.027189
R-HSA-210500	Glutamate Neurotransmitter Release Cycle	-0.584669268	-1.64047	0.007899	0.037724	0.02802
R-HSA-389513	CTLA4 inhibitory signaling	0.596689492	1.725419	0.00793	0.03775	0.02804
R-HSA-211935	Fatty acids	-0.68274361	-1.64924	0.008102	0.038447	0.028558

R-HSA-164952	The role of Nef in HIV-1 replication and disease pathogenesis	0.547689727	1.655133	0.008597	0.040665	0.030205
R-HSA-5686938	Regulation of TLR by endogenous ligand	0.605772286	1.696166	0.008748	0.041245	0.030636
R-HSA-5656169	Termination of translesion DNA synthesis	0.520125882	1.632032	0.008807	0.041391	0.030744
R-HSA-379716	Cytosolic tRNA aminoacylation	0.581348282	1.720606	0.009102	0.04264	0.031672
R-HSA-69183	Processive synthesis on the lagging strand	0.670958224	1.759586	0.009168	0.042709	0.031723
R-HSA-381340	Transcriptional regulation of white adipocyte differentiation	-0.433878732	-1.55515	0.009175	0.042709	0.031723
R-HSA-8862803	Deregulated CDK5 triggers multiple neurodegenerative pathways in Alzheimer's disease models	0.589137107	1.681241	0.009682	0.044507	0.033059
R-HSA-8863678	Neurodegenerative Diseases	0.589137107	1.681241	0.009682	0.044507	0.033059
R-HSA-4085377	SUMOylation of SUMOylation proteins	0.500298594	1.627793	0.009643	0.044507	0.033059
R-HSA-2565942	Regulation of PLK1 Activity at G2/M Transition	0.395031674	1.498042	0.009682	0.044507	0.033059
R-HSA-5368287	Mitochondrial translation	0.418239177	1.53302	0.009786	0.044843	0.033309
R-HSA-168325	Viral Messenger RNA Synthesis	0.469903895	1.597897	0.009819	0.044856	0.033318
R-HSA-373076	Class A/1 (Rhodopsin-like receptors)	0.285189694	1.314364	0.010037	0.04571	0.033952
R-HSA-383280	Nuclear Receptor transcription pathway	-0.480532074	-1.61814	0.010423	0.047248	0.035095
R-HSA-382556	ABC-family proteins mediated transport	0.371779549	1.46786	0.010439	0.047248	0.035095
R-HSA-6805567	Keratinization	0.364150724	1.400477	0.010471	0.047248	0.035095
R-HSA-445355	Smooth Muscle Contraction	-0.512437642	-1.64367	0.010573	0.047557	0.035324
R-HSA-5610787	Hedgehog 'off' state	0.364682198	1.465759	0.010604	0.047557	0.035324
R-HSA-2243919	Crosslinking of collagen fibrils	-0.651468409	-1.66768	0.011049	0.049252	0.036584
R-HSA-416572	Sema4D induced cell migration and growth-cone collapse	-0.588184783	-1.60219	0.011044	0.049252	0.036584
R-HSA-8956321	Nucleotide salvage	0.600499691	1.681403	0.011157	0.049581	0.036828
R-HSA-1442490	Collagen degradation	-0.452620326	-1.55353	0.011259	0.049884	0.037053
