# Supplementary figures and images for: Reactivation of low avidity tumor-specific CD8+ T cells associates with immunotherapeutic efficacy of anti-PD-1
Source: J Immunother Cancer. 2023 Aug 16;11(8):e007114. doi: 10.1136/jitc-2023-007114 (PMC10432680; doi:10.1136/jitc-2023-007114)

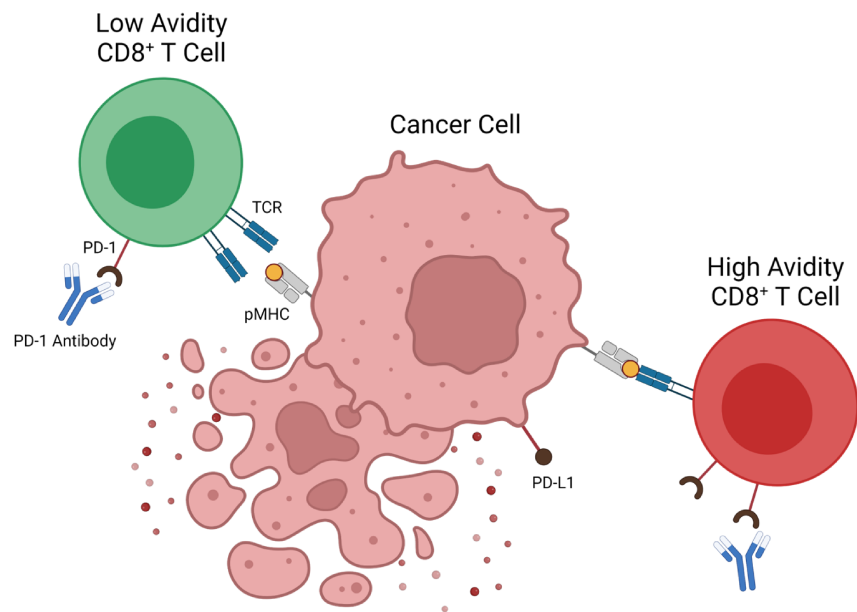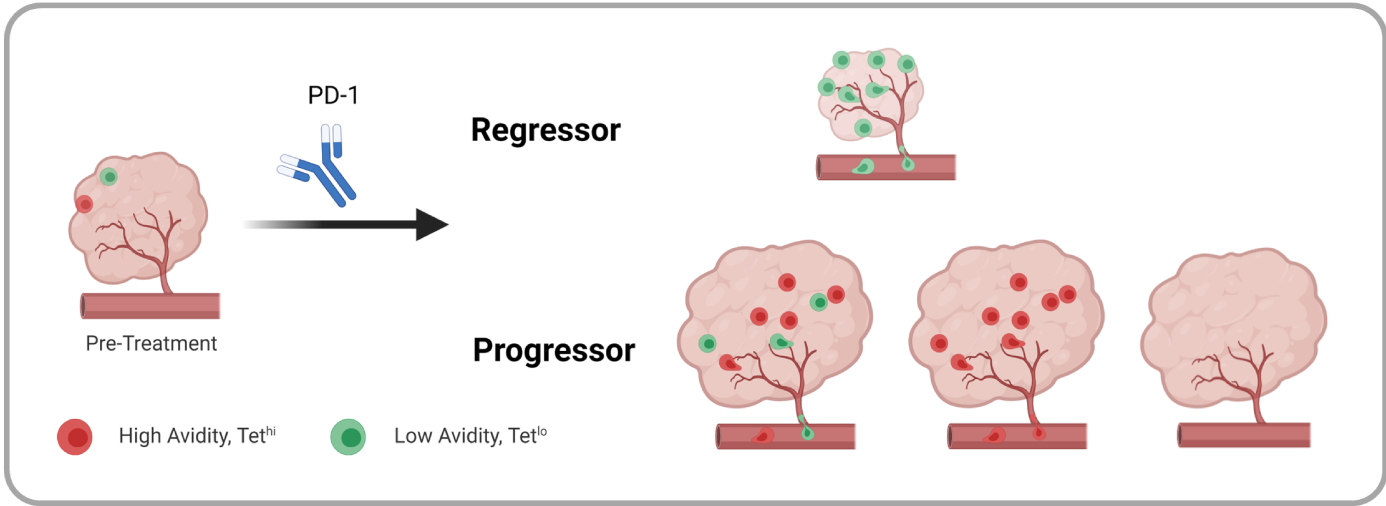

Supplement: Supplementary data [file jitc-2023-007114supp004.pdf]
